# Supplementary material for: Cohesin acts as a transcriptional gatekeeper by restraining pause–release to promote processive elongation
Source: Nat Commun. 2026 Aug 24;17:8487. doi: 10.1038/s41467-026-76738-3 (PMC13503787; doi:10.1038/s41467-026-76738-3)

Suppelementary Information

Cohesin acts as a transcriptional gatekeeper by restraining pause–release to promote processive elongation

Supplementary Figures

Supplementary Figure 1: Cohesin is a major determinant of 3D chromatin structure.

Supplementary Figure 2: Cohesin loss has limited effects on steady-state gene expression.

Supplementary Figure 3: Cohesin promotes Pol II pausing at highly paused genes.

Supplementary Figure 4: Cohesin constrains early Pol II elongation and stabilizes transcription elongation to prevent premature termination.

Supplementary Figure 5: Cohesin and its loader coordinately colocalize with RNA Polymerase II at promoters.

Supplementary Figure 6: Cohesin and its loader engage the transcription machinery at the pause–release transition.

Supplementary Figure 7: Cohesin maintains the H3K27ac mark at promoters.

Supplementary Figure 8: Cohesin regulates H3K27ac distribution via promoter–enhancer contacts.

Supplementary Figure 9: Genes with modest expression changes upon cohesin loss show reduced Pol II recruitment and enhanced pause release.

Supplementary Figure 10: Simulations suggest compensatory effects of reduced Pol II recruitment and enhanced pause release.

Supplementary Figure 11: Cohesin depletion impairs rapid gene induction.

Supplementary Figure 12: Schematic model showing how cohesin regulates transcription.

Supplementary Methods

References

Uncropped blot and gel scans for Supplementary Figures

Supplementary Figure 1a

Supplementary Figure 2d

Supplementary Figure 3l

Supplementary Figure 4f

Supplementary Figure 6d

Supplementary Figure 6e

Supplementary Figure 6f

Supplementary Figure 6g

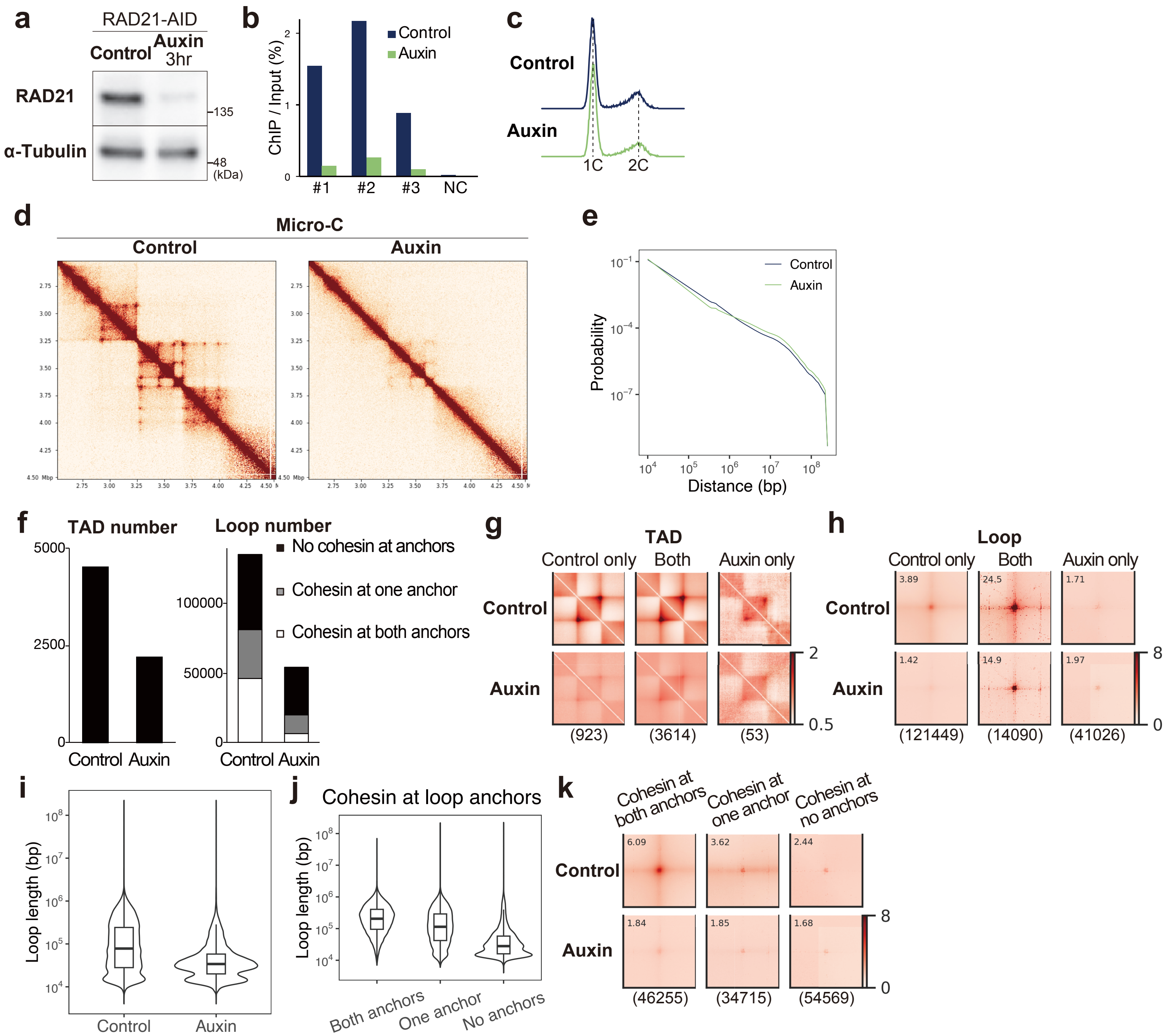

Supplementary Figure 1: Cohesin is a major determinant of 3D chromatin structure.

a Western blots of RAD21 and α-tubulin in whole-cell extracts. HCT-116 cells expressing AID-tagged RAD21 were treated with 0.5 mM indole-3-acetic acid (auxin) for 3 hours. RAD21 protein levels, normalized to α-tubulin, were reduced to ~6% of control levels upon auxin treatment.

b ChIP–qPCR of RAD21 at three RAD21 ChIP–seq peaks and a negative control (NC) site. An anti-GFP antibody was used to detect RAD21 fused at its C-terminus with a mini auxin-inducible degron (mAID) and mClover. Source data are provided as a Source Data file.

c Histograms of DNA content in control and auxin-treated cells. Fixed cells were stained with propidium iodide, and DNA content was measured by flow cytometry after 3-h auxin treatment. Source data are provided as a Source Data file.

d 5-kb resolution Micro-C contact maps in control and auxin-treated cells. Shown is a 2-Mb region on chromosome 18.

e Contact probability curves derived from Micro-C in control and auxin-treated cells.

f Bar plots showing the number of TADs and loops identified by Micro-C in control and auxin-treated cells. TADs were called at 25-kb resolution. Loops were detected at 1-, 2-, and 5-kb resolutions, merged, and categorized by the presence or absence of RAD21 peaks at loop anchors.

g Aggregate plots of TADs detected only in control cells, only in auxin-treated cells, or shared between both conditions. The number of TADs in each category is indicated in parentheses.

h Aggregate plots of loops detected only in control cells, only in auxin-treated cells, or shared between both conditions. The number of loops in each category is indicated in parentheses.

i Violin plots showing the distribution of loop lengths derived from Micro-C in control and auxin-treated cells.

j Violin plots showing the distribution of loop lengths in control cells, categorized by the presence or absence of RAD21 peaks at loop anchors.

k Aggregate plots of loops categorized by the presence or absence of RAD21 peaks at loop anchors. The number of loops in each category is indicated in parentheses.

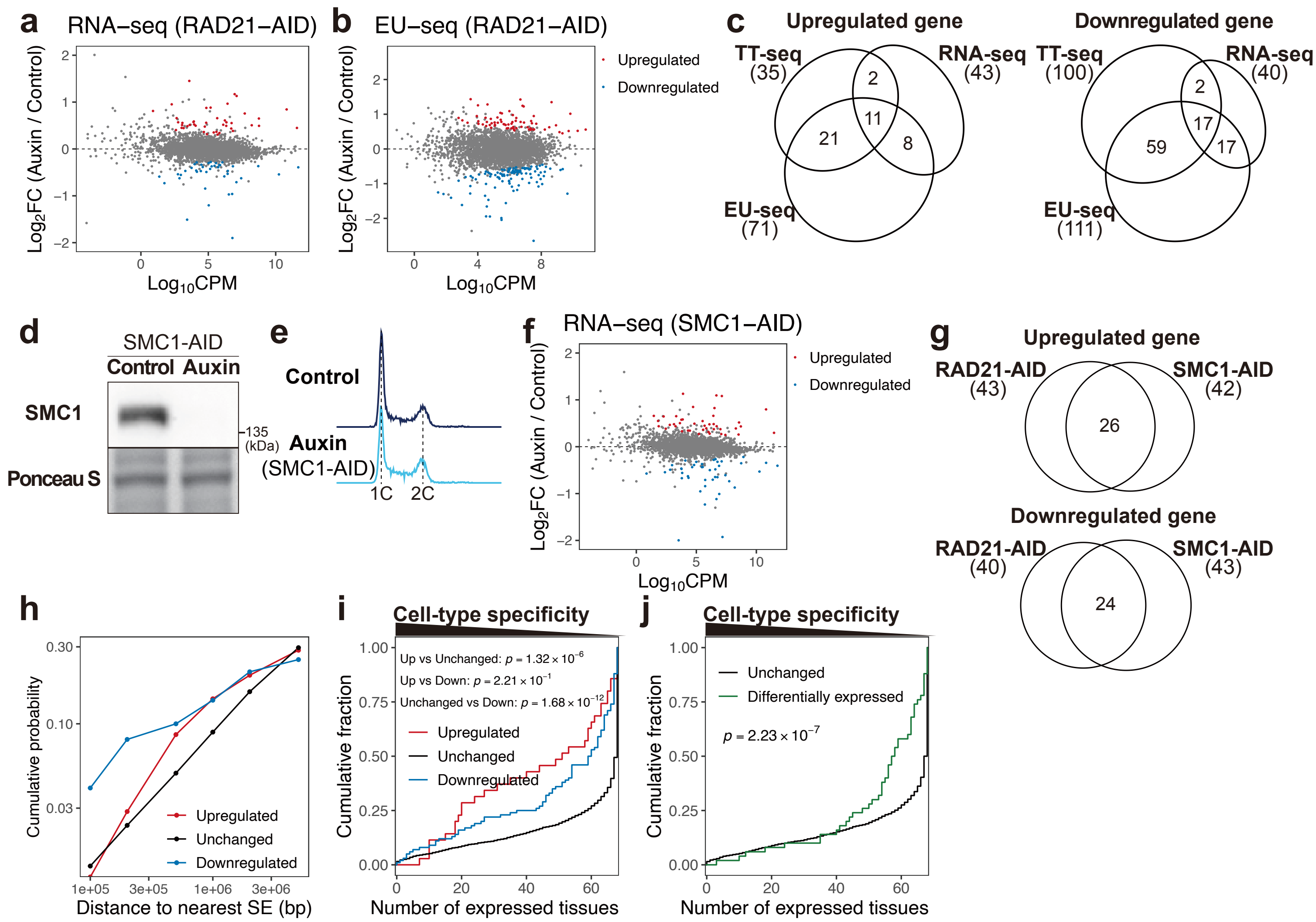

Supplementary Figure 2: Cohesin loss has limited effects on steady-state gene expression.

a MA plot of RNA-seq showing gene expression changes upon auxin-induced cohesin depletion (3,597 genes). Differentially expressed genes were identified using edgeR (FDR < 0.01) based on three biological replicates.

b MA plot of EU-seq showing gene expression changes upon auxin-induced cohesin depletion (3,597 genes). Differentially expressed genes were identified using edgeR (FDR < 0.05) based on two biological replicates.

c Venn diagrams showing the overlap among upregulated and downregulated genes identified from TT-seq, RNA-seq, and EU-seq.

d Western blots of SMC1 and Ponceau staining in whole-cell extracts. HCT-116 cells expressing AID-tagged SMC1 were treated with 0.5 mM indole-3-acetic acid (auxin) for 3 h. SMC1 protein levels, normalized to Ponceau, decreased to ~1% of control levels upon auxin treatment.

e Histograms of DNA content in control and auxin-treated cells expressing SMC1-AID. Fixed cells were stained with propidium iodide, and DNA content was measured by flow cytometry after 3-hour auxin treatment. Source data are provided as a Source Data file.

f MA plot of RNA-seq showing gene expression changes upon auxin-induced cohesin (SMC1) depletion (3,597 genes). Differentially expressed genes were identified using edgeR (FDR < 0.01) based on three biological replicates.

g Venn diagrams showing the overlap between upregulated and downregulated genes identified from RNA-seq in RAD21-depleted and SMC1-depleted cells.

h Cumulative probability distributions of distances to the nearest super enhancers for genes upregulated, unchanged, and downregulated upon cohesin depletion identified by TT-seq; n = 35, 3,462 and 100 genes, respectively. Statistical significance was calculated using a two-sided Kolmogorov–Smirnov test.

i Cumulative distributions of cell-type specificity for genes upregulated, unchanged, and downregulated upon cohesin depletion identified by TT-seq. Statistical significance was calculated using a two-sided Kolmogorov–Smirnov test.

j Cumulative distributions of cell-type specificity for differentially expressed genes shared between RAD21-depleted and SMC1-depleted cells and other genes; n = 50 and 3,547 genes, respectively. Statistical significance was calculated using a two-sided Kolmogorov–Smirnov test.

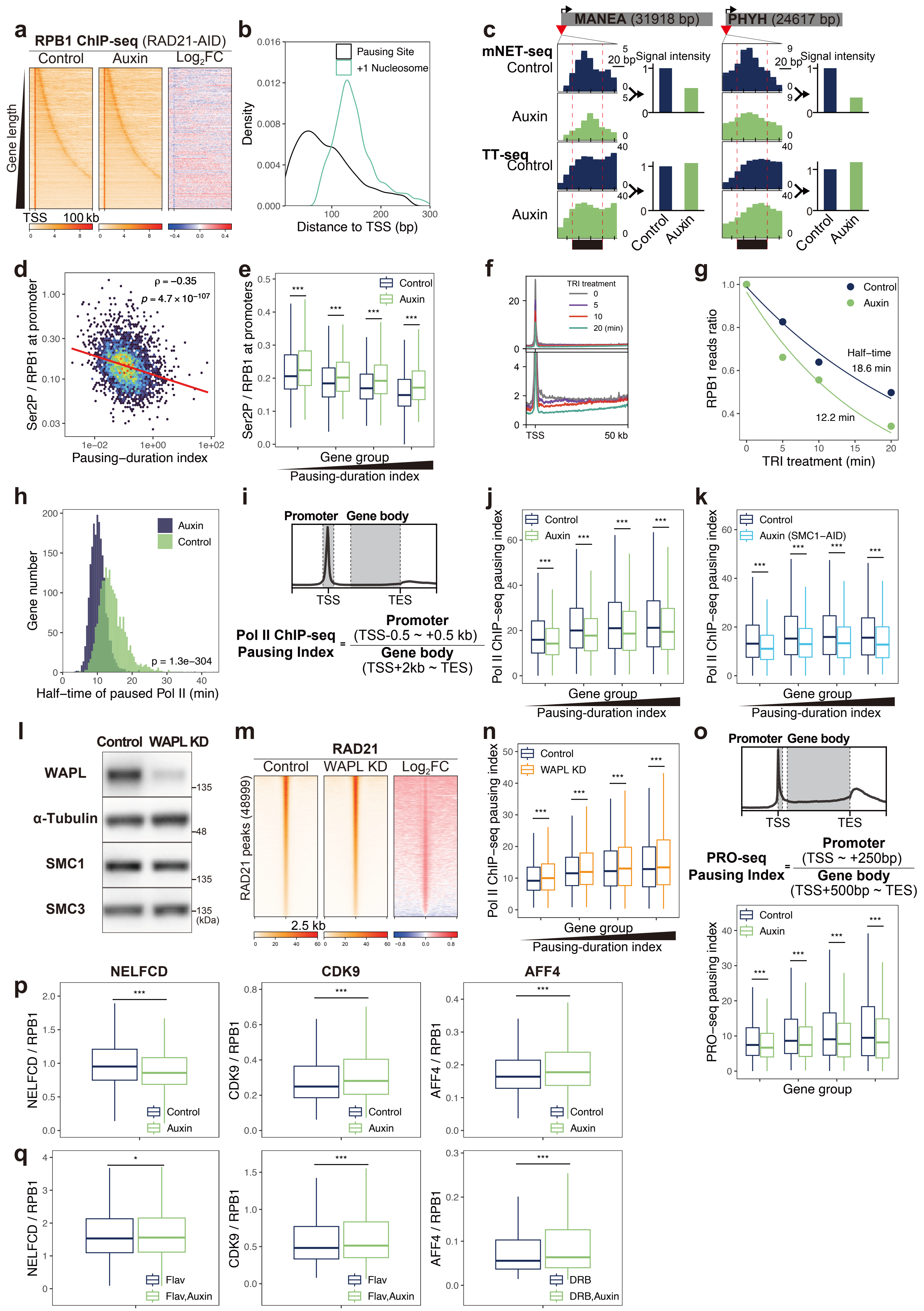
a
RPB1 ChIP-seq (RAD21-AID)
Control
Auxin
Log_2FC
Gene length
TSS
100 kb
b
Pausing Site
+1 Nucleosome
Density
Distance to TSS (bp)
c
MANEA (31918 bp)
PHYH (24617 bp)
mNET-seq
Control
Auxin
TT-seq
Control
Auxin
20 bp
Signal intensity
Control
Auxin
d
ρ = −0.35
$p = 4.7 \times 10^{-107}$
Ser2P / RPB1 at promoter
Pausing−duration index
e
Control
Auxin
Ser2P / RPB1 at promoters
Gene group
Pausing-duration index
f
TRI treatment
0
5
10
20 (min)
TSS
50 kb
g
Control
Auxin
Half−time
18.6 min
12.2 min
RPB1 reads ratio
TRI treatment (min)
h
Auxin
Control
Gene number
p = 1.3e−304
Half−time of paused Pol II (min)
i
Promoter
Gene body
TSS
TES
Pol II ChIP-seq Pausing Index = Promoter (TSS-0.5 ~ +0.5 kb) / Gene body (TSS+2kb ~ TES)
j
Control
Auxin
Pol II ChIP−seq pausing index
Gene group
Pausing-duration index
k
Control
Auxin (SMC1−AID)
Pol II ChIP−seq pausing index
Gene group
Pausing-duration index
l
Control
WAPL KD
WAPL
α-Tubulin
SMC1
SMC3
135
48
135
135
(kDa)
m
RAD21
Control
WAPL KD
Log_2FC
RAD21 peaks (48999)
2.5 kb
n
Control
WAPL KD
Pol II ChIP−seq pausing index
Gene group
Pausing-duration index
o
Promoter
Gene body
TSS
TES
PRO-seq Pausing Index = Promoter (TSS ~ +250bp) / Gene body (TSS+500bp ~ TES)
Control
Auxin
PRO−seq pausing index
Gene group
p
NELFCD
CDK9
AFF4
NELFCD / RPB1
CDK9 / RPB1
AFF4 / RPB1
Control
Auxin
q
NELFCD / RPB1
CDK9 / RPB1
AFF4 / RPB1
Flav
Flav,Auxin
DRB
DRB,Auxin

Supplementary Figure 3: Cohesin promotes Pol II pausing at highly paused genes.
a Heatmaps showing RPB1 ChIP-seq signals across genes (TSS −10 kb to +100 kb) for 3,597 genes in control and auxin-treated cells. Genes are aligned at their transcription start site (TSS) and sorted by gene length.

b Histograms of distances from the TSSs to pausing sites and nucleosomes for 3,597 genes. Pausing sites were defined as the highest mNET-seq peak between the TSS and the +1 nucleosome. +1 nucleosome positions were determined by MNase–ChIP–seq (H3K4me3).

c Representative gene showing reduced mNET-seq signal at the pausing site upon cohesin depletion, while TT-seq signal at the same site remained largely unchanged. The red arrowhead indicates the position of the pausing site. Box plots showing quantified mNET-seq and TT-seq signal intensities within ±25 bp of the pausing site of the representative gene, used to calculate the pausing-duration index.

d Comparison of the pausing-duration index and Ser2P/RPB1 levels at promoters (TSS -1 kb to +1 kb) for 3,597 genes in control cells. P-value (*p*) and Spearman's correlation coefficient (ρ) were calculated using two-sided Spearman's rank correlation test. The red line indicates the linear regression fit

e Box plots showing Ser2P/RPB1 levels at promoters (TSS -1 kb to +1 kb) in control and auxin-treated cells. Gene groups are the same as those shown in Fig. 1d. Statistical significance was calculated using a two-sided Wilcoxon signed-rank test (***$p < 0.001$). Exact P-values from left to right: 3.98×10^{-19}, 2.76×10^{-33}, 6.72×10^{-34}, and 1.49×10^{-39}.

f Average ChIP-seq profiles of RNA polymerase II (Pol II) for 850 genes longer than 50 kb (TSS -5 to + 50 kb) after triptolide (TRI) treatment. RPB1 ChIP-seq was performed at 0, 5, 10, and 20 min after TRI treatment.

g Exponential decay model estimating the half-life of paused Pol II at a representative gene (SLC25A33). RPB1 promoter occupancy (TSS −1 kb to +1 kb) was quantified at multiple time points after TRI treatment, and an exponential decay curve was fitted.

h Histograms of the half-life of paused Pol II in control and auxin-treated cells. Half-lives were estimated by fitting an exponential decay model to RPB1 promoter occupancy (TSS −1 kb to +1 kb) over time following triptolide (TRI) treatment. A total of 2,004 genes exhibiting a continual reduction in promoter RPB1 occupancy throughout the time-course experiment were analyzed. Mean half-lives: control, 14.4 min; auxin, 10.3 min. Statistical significance was calculated using a two-sided Wilcoxon signed-rank test.

i Pol II ChIP-seq pausing index is calculated as the ratio of Pol II occupancy at the promoter (TSS -0.5 kb to + 0.5 kb) to that in the gene body (TSS + 2kb to TES).

j Box plots showing Pol II ChIP-seq pausing index in control and auxin-treated cells. Gene groups are the same as those shown in Fig. 1d. Statistical significance was calculated using a two-sided Wilcoxon signed-rank test (***$p < 0.001$). Exact P-values from left to right: 3.80×10^{-34}, 1.36×10^{-41}, 4.32×10^{-54}, and 2.98×10^{-27}.

k Box plot showing Pol II ChIP-seq pausing index in control and auxin-treated cells expressing AID-tagged SMC1. Gene groups are the same as those shown in Fig. 1d. Statistical significance was calculated using a two-sided Wilcoxon signed-rank test (***$p < 0.001$). Exact P-values from left to right: 2.97×10^{-75}, 4.74×10^{-72}, 1.26×10^{-64}, and 8.63×10^{-69}.

l Western blots of WAPL, α-tubulin, SMC1, and SMC3 in whole-cell extracts. Cells were treated with WAPL-targeting siRNA for two days. WAPL protein levels, normalized to α-tubulin, decreased to ~22% of control levels, upon WAPL knockdown.

m Heatmaps showing RAD21 ChIP-seq siganls in control and WAPL-knockdown cells at 48,999 RAD21 ChIP-seq peaks. Peaks are sorted by RAD21 intensity in control cells.

n Box plots showing Pol II ChIP-seq pausing index in control and WAPL-knockdown cells. Gene groups are the same as those shown in Fig. 1d. Statistical significance was calculated using a two-sided Wilcoxon signed-rank test (***$p < 0.001$). Exact P-values from left to right: 1.37×10^{-13}, 1.27×10^{-13}, 1.39×10^{-13}, and 1.74×10^{-16}.

o PRO-seq pausing index is calculated as the ratio of PRO-seq signal density at the promoter (TSS to +250 bp) to that in the gene body (TSS + 500 bp to TES). Box plots of PRO-seq pausing index in control and auxin-treated cells. PRO-seq data were from Rao et al. (2017). Gene groups are the same as those shown in Fig. 1d. Statistical significance was calculated using a two-sided Wilcoxon signed-rank test (***$p < 0.001$). Exact P-values from left to right: 4.58×10^{-42}, 1.94×10^{-50}, 4.54×10^{-46}, and 2.19×10^{-42}.

p Box plots showing the relative levels of NELFCD, CDK9, and AFF4 to RPB1 promoters (TSS -1 kb to +1 kb) for 3,597 genes in control and auxin-treated cells. Statistical significance was calculated using a two-sided Wilcoxon signed-rank test (***$p < 0.001$). Exact P-values: 1.89×10^{-55} (NELFCD), 2.07×10^{-58} (CDK9), and 1.30×10^{-35} (AFF4).

q Box plots showing the relative levels of NELFCD, CDK9, and AFF4 to RPB1 at promoters (TSS -1 kb to +1 kb) for 3,597 genes in control and auxin-treated cells, in the presence of flavopiridol or DRB. Statistical significance was calculated using a two-sided Wilcoxon signed-rank test (*$p < 0.05$, ***$p < 0.001$). Exact P-values: 4.00×10^{-2} (NELFCD), 1.26×10^{-15} (CDK9), and 2.05×10^{-77} (AFF4).

All box plots show the median (centre), 25th and 75th percentiles (box bounds), and whiskers extending to the smallest and largest values within 1.5× the interquartile range from the quartiles.

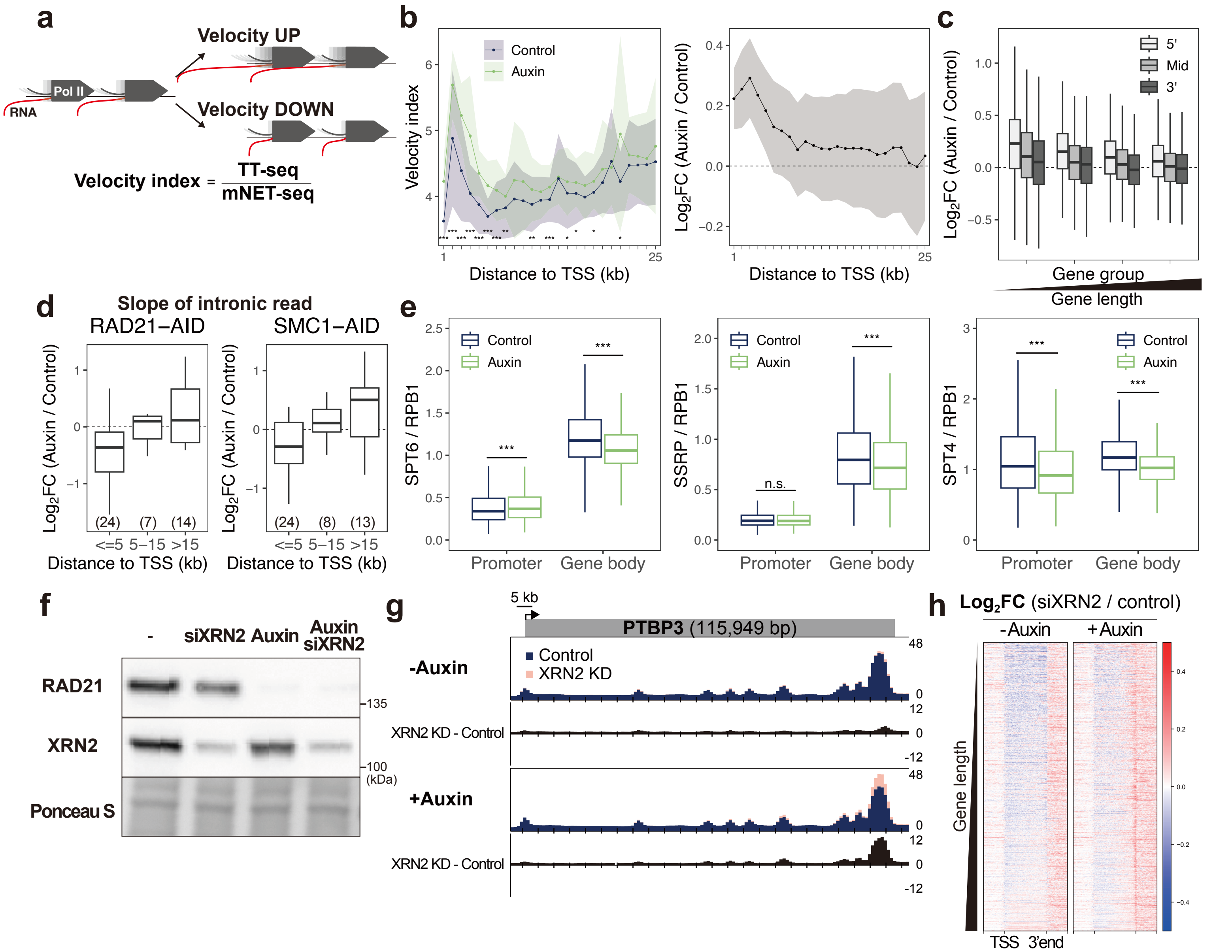

Supplementary Figure 4: Cohesin constrains early Pol II elongation and stabilizes transcription elongation to prevent premature termination.

a Velocity index was calculated as the ratio of TT-seq to mNET-seq across gene bodies.

b Line plots with error shading showing the velocity index in control and auxin-treated cells (left), and their $\log_2$ fold changes (auxin/control) (right). Values are shown from the TSS to +25 kb in 1-kb bins, where available, using data from 3,597 genes. Data represent mean ± 95% confidence intervals calculated across genes. Statistical significance was calculated using a two-sided Wilcoxon signed-rank test (*$p < 0.05$, **$p < 0.01$, ***$p < 0.001$).

c Box plots of $\log_2$ fold changes (auxin/control) in the velocity index across gene bodies. Gene bodies were divided into three equal regions (5', Mid, and 3') from the TSS to 3'gene end. A total of 3,597 genes were evenly divided into four groups based on their length.

d Box plots showing $\log_2$ fold changes (auxin/control) in the slope of RNA-seq intronic reads in RAD21-depleted (left) and SMC1-depleted cells (right). The slope was estimated by fitting a linear model to intronic read coverage. Introns longer than 1 kb with a negative slope and an adjusted $R^2 > 0.5$ were included in the analysis. The number of introns analyzed is indicated in parentheses.

e Box plots of the relative levels of SPT6 (left), SSRP (FACT complex) (mid), and SPT4 (DSIF complex) (right) to RPB1 in control and auxin-treated cells at promoters (TSS -1 kb to +1 kb) and gene bodies (TSS +1 kb to 3'gene end) for 3,597 genes. Statistical significance was calculated using a two-sided Wilcoxon signed-rank test (***$p < 0.001$). Exact P-values: 1.78×10^{-26} (SPT6 promoter), 8.75×10^{-249} (SPT6 gene body), 0.428 (SSRP promoter), 3.91×10^{-145} (SSRP gene body), 4.48×10^{-69} (SPT4 promoter), $<1\times10^{-300}$ (SPT4 gene body).

f Western blots of RAD21 and XRN2, with Ponceau staining in whole-cell extract. XRN2 protein levels, normalized to Ponceau, decreased to ~22% of control levels upon siXRN2 treatment.

g Representative gene showing increased RNA-seq signal upon XRN2 knockdown, which was further enhanced upon auxin-induced cohesin depletion. Difference tracks (XRN2 KD – Control) are shown below each condition.

h Heatmaps of $\log_2$ fold changes (siXRN2/control and auxin + siXRN2/auxin) in RNA-seq across genes (TSS to 3' gene end) for 3,597 genes. Genes are aligned at their 3'gene end and sorted by gene length.

All box plots show the median (centre), 25th and 75th percentiles (box bounds), and whiskers extending to the smallest and largest values within 1.5× the interquartile range (IQR) from the quartiles.

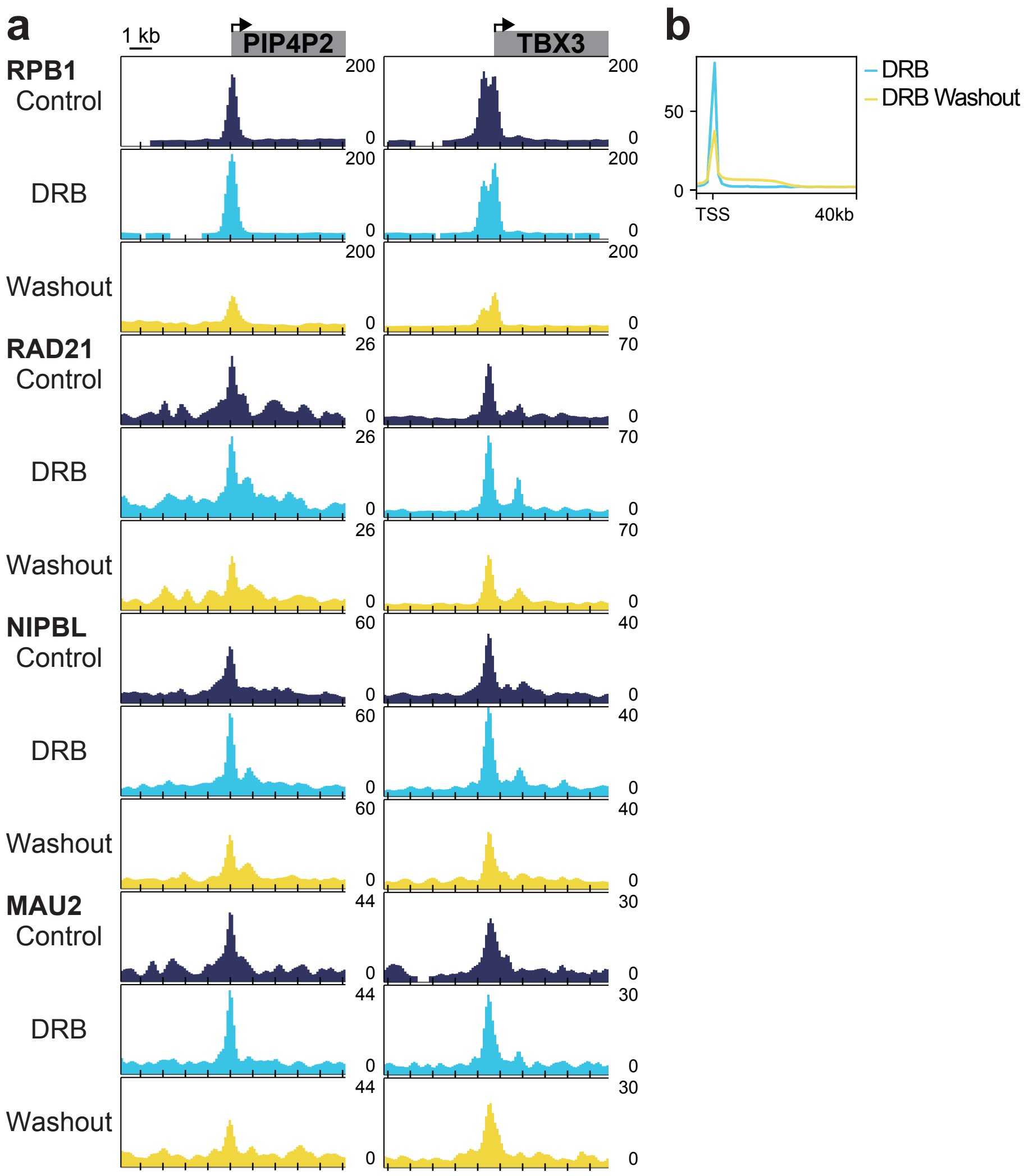

Supplementary Figure 5: Cohesin and its loader coordinately colocalize with RNA Polymerase II at promoters.
a Representative genes showing increased promoter binding of RPB1, RAD21, NIPBL, and MAU2 upon DRB treatment, which was reduced upon DRB washout (TSS –5 kb to +5 kb).

b Average ChIP-seq profiles of RPB1 under DRB treatment and DRB washout conditions at 1,766 genes longer than 40 kb (TSS -5 kb to +40 kb).

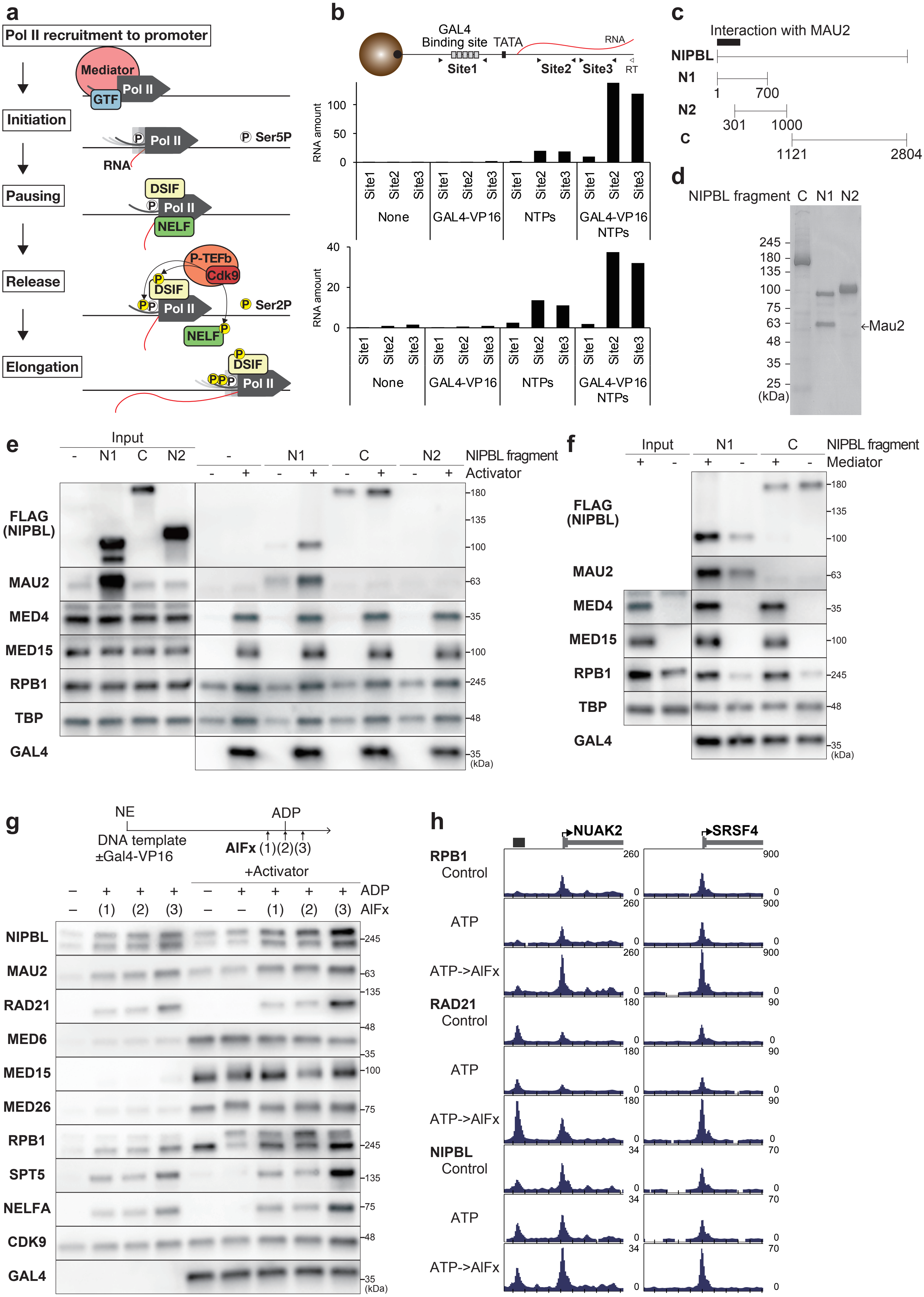

a
Pol II recruitment to promoter
Mediator
Pol II
GTF
Initiation
Pol II
Ser5P
RNA
Pausing
DSIF
Pol II
NELF
Release
P-TEFb
Cdk9
DSIF
Pol II
Ser2P
NELF
Elongation
DSIF
Pol II
b
GAL4
Binding site
TATA
RNA
Site1
Site2
Site3
RT
RNA amount
100
50
0
Site1
Site2
Site3
None
GAL4-VP16
NTPs
GAL4-VP16
NTPs
RNA amount
40
20
0
c
Interaction with MAU2
NIPBL
N1
1
700
N2
301
1000
C
1121
2804
d
NIPBL fragment
C
N1
N2
245
180
135
100
75
63
48
35
25
(kDa)
←Mau2
e
Input
-
N1
C
N2
NIPBL fragment
Activator
FLAG (NIPBL)
MAU2
MED4
MED15
RPB1
TBP
GAL4
180
135
100
63
35
100
245
48
35
(kDa)
f
Input
N1
C
NIPBL fragment
Mediator
FLAG (NIPBL)
MAU2
MED4
MED15
RPB1
TBP
GAL4
180
135
100
63
35
100
245
48
35
(kDa)
g
NE
ADP
DNA template
±Gal4-VP16
AlFx (1)(2)(3)
+Activator
ADP
AlFx
NIPBL
MAU2
RAD21
MED6
MED15
MED26
RPB1
SPT5
NELFA
CDK9
GAL4
245
63
135
48
35
100
75
245
135
75
48
35
(kDa)
h
NUAK2
SRSF4
RPB1
Control
ATP
ATP->AlFx
RAD21
Control
ATP
ATP->AlFx
NIPBL
Control
ATP
ATP->AlFx
260
900
180
90
34
70
0

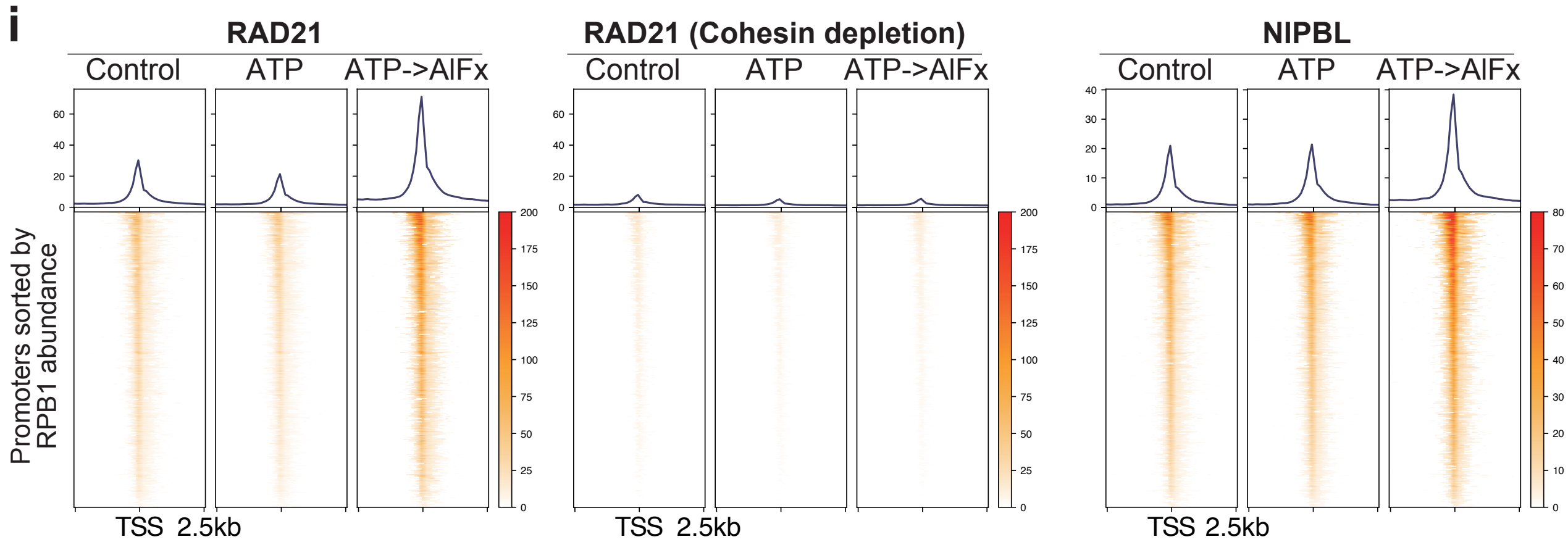

Supplementary Figure 6: Cohesin and its loader engage the transcription machinery at the pause–release transition.
a Schematic illustration of transcriptional stages. First, RNA polymerase II (Pol II) is recruited to promoters bound by general transcription factors (GTFs) and the Mediator complex, and it initiates transcription. Shortly after initiation, Pol II pauses with the help of NELF and DSIF. CDK9, a component of P-TEFb, phosphorylates the Pol II C-terminal domain (CTD), NELF, and DSIF to promote pause release. Pol II then enters productive elongation.

b Schematic of primer design to detect RNA production (top). For qPCR, Site1 targets the GAL4-binding sites, whereas Site2 and Site3 target the gene body downstream of the TATA box. RT indicates the position of the oligonucleotide used for reverse transcription. Box plots show RNA amounts quantified by qPCR under the following conditions: none, GAL4-VP16, NTPs, and GAL4-VP16 + NTPs, from two independent replicates (bottom). A value of 1 corresponds to the signal equivalent to 1 μg of template DNA as determined by a standard curve of the template DNA. Source data are provided as a Source Data file.

c Schematic representation of the three recombinant NIPBL fragments used in the *in vitro* transcription assay. The black box indicates the N-terminal region of NIPBL required for interaction with MAU2.

d Coomassie blue staining of purified recmbinant NIPBL fragments (C, N1, and N2). The fragments were expressed in insect cells and purified. MAU2 was co-expressed with the N1 fragment and co-purified.

e *In vitro* transcription assay with NIPBL fragments. Nuclear extracts (Input) were mixed with FLAG-tagged recombinant NIPBL fragments, then incubated with template DNA preloaded with GAL4-VP16.

f *In vitro* transcription assay with Mediator depletion on the recruitment of NIPBL mutants (N1 and C). The mediator complex was depleted from nuclear extract using antibodies against MED4, MED15, and MED25. Nuclear extracts (Input) were mixed with FLAG-tagged recombinant NIPBL fragments, then incubated with template DNA preloaded with GAL4-VP16.

g *In vitro* transcription assay with cohesin ATPase inhibition by AlFx. Template DNA, preloaded with GAL4-VP16, was incubated with nuclear extracts (Input). ADP was added 20 min after the start of incubation. AlFx was added either 3 minutes before (1), simultaneously with (2), or 3 minutes after (3) ADP addition.

h Representative gene showing enhanced promoter binding of RPB1, RAD21, and NIPBL upon ATP->AlFx treatment. Black box indicates the position of a cohesin peak.

i Heatmaps of RAD21 and NIPBL CUT&Tag signals at promoters (TSS −2.5 kb to +2.5 kb) for 3,597 genes under control, ATP, and ATP->AlFx conditions, with or without cohesin depletion. Permeabilized cells were treated with ATP for 10 min or with ATP for 5 min followed by AlFx for 5 min.

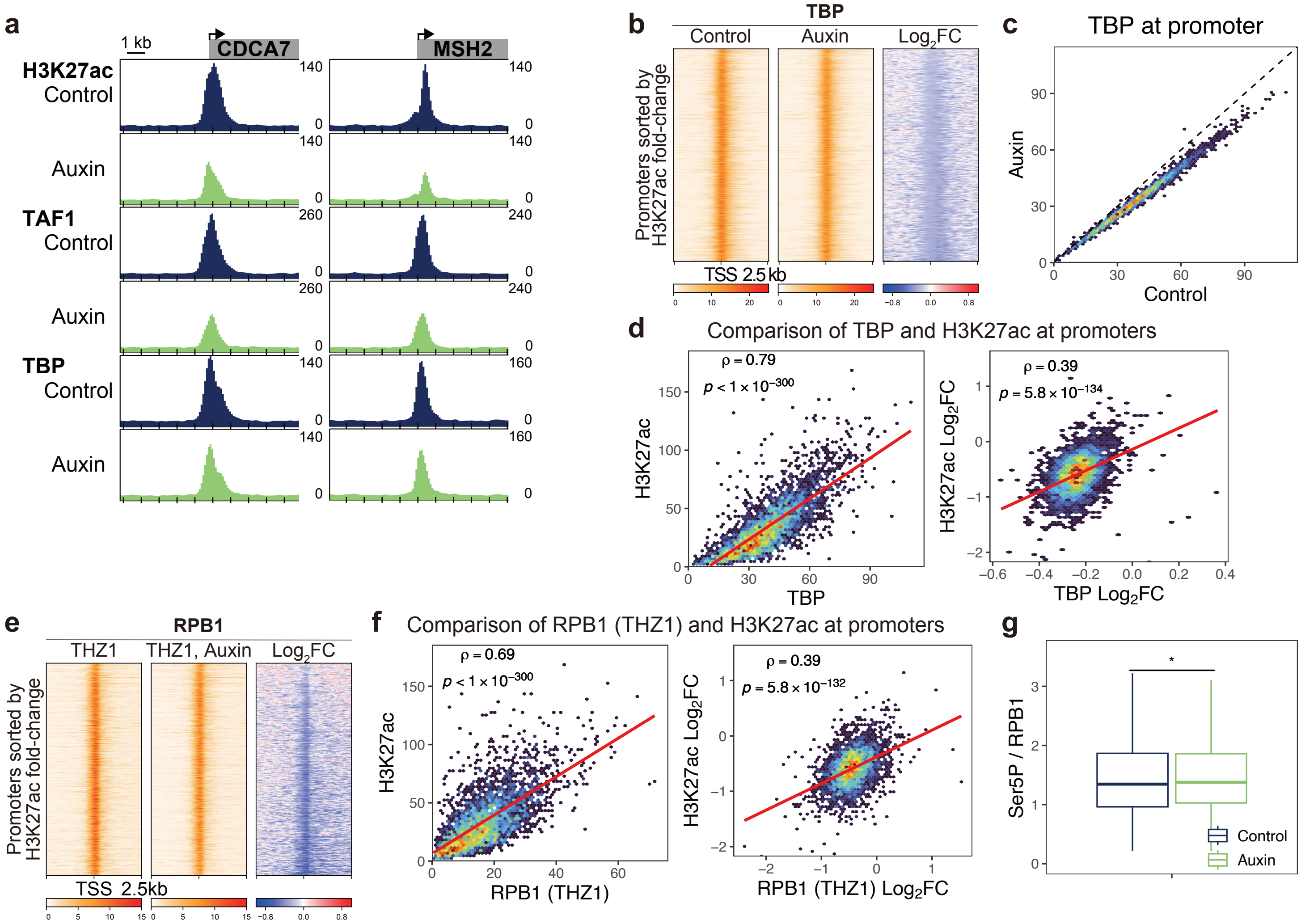

Supplementary Figure 7: Cohesin maintains the H3K27ac mark at promoters.

a Representative genes showing decreased promoter binding of H3K27ac, TAF1, and TBP upon auxin treatment (TSS –5 kb to +5 kb).

b Heatmaps of TBP ChIP-seq signals at promoters (TSS -2.5 kb to +2.5 kb) in control and auxin-treated cells. Promoters are shown in the same order as in Fig. 5a.

c Comparison of promoter TBP (TSS -1 kb to +1 kb) between control and auxin-treated cells for 3,597 genes. Upon cohesin depletion, TBP levels decreased at 92% of promoters and increased at 8% (FDR < 0.05, one-sided upper-tail binomial test).

d Comparison between promoter TBP and H3K27ac (TSS -1 kb to +1 kb) in control cells, and their $\log_2$ fold changes (auxin/control) for 3,597 genes. P-value (*p*) and Spearman's correlation coefficient (ρ) were calculated using two-sided Spearman's rank correlation test. Exact P-values for promoter TBP and H3K27ac: $p < 1.0\times10^{-300}$; for their log2 fold changes: 5.76×10^{-134}.

e Heatmaps of RPB1 ChIP-seq signals in the presence of THZ1 at promoters (TSS -2.5 kb to +2.5 kb) in control and auxin-treated cells. Promoters are shown in the same order as in Fig. 5a.

f Comparison between promoter RPB1 in the presence of THZ1 and H3K27ac (TSS -1 kb to +1 kb) in control cells (left), and their $\log_2$ fold changes (auxin/control) for 3,597 genes (right). P-value (*p*) and Spearman's correlation coefficient (ρ) were calculated using two-sided Spearman's rank correlation test. Exact P-values for promoter RPB1 and H3K27ac: $p < 1.0\times10^{-300}$; for their log2 fold changes: 5.84×10^{-132}.

g Box plots of the relative Ser5P levels to RPB1 at promoters (TSS -1 kb to +1 kb) for 3,597 genes in control and auxin-treated cells. Statistical significance was calculated using a two-sided Wilcoxon signed-rank test. (*$p < 0.05$). Exact p-values: 1.67×10^{-2}. Box plots show the median (centre), 25th and 75th percentiles (box bounds), and whiskers extending to the smallest and largest values within 1.5× the interquartile range from the quartiles.

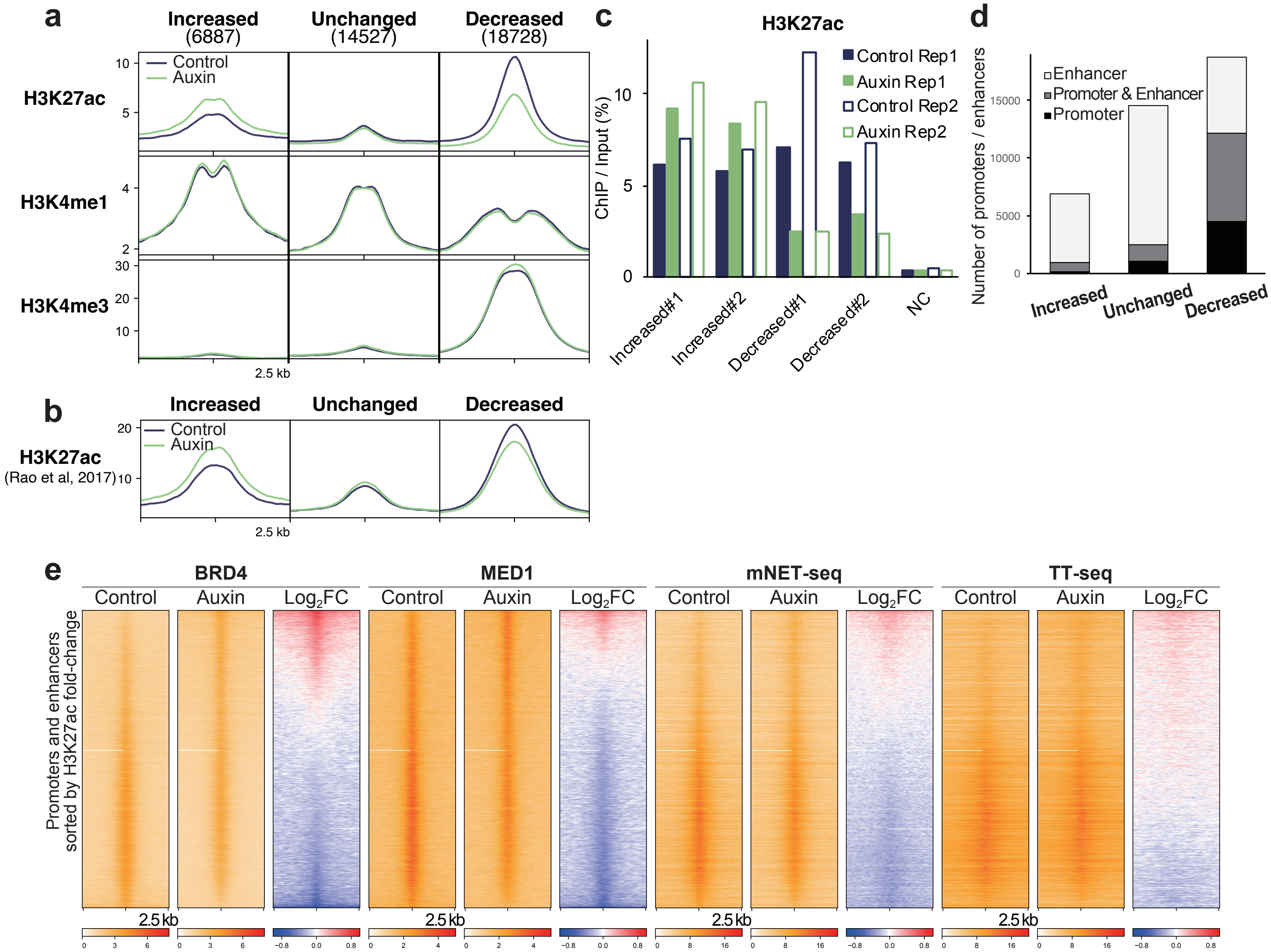

Supplementary Figure 8: Cohesin regulates H3K27ac distribution via promoter–enhancer contacts.

a Average ChIP-seq profiles of H3K27ac, H3K4me1, and H3K4me3 at regulatory elements (promoters and enhancers). Regulatory elements were classified as increased, unchanged, or decreased based on their H3K27ac response to cohesin depletion. The number of elements in each category is shown in parentheses.

b Average ChIP-seq profiles of H3K27ac from Rao et al. (2017).at regulatory elements (promoters and enhancers). Regulatory elements were classified as increased, unchanged, or decreased as in a.

c ChIP–qPCR of H3K27ac at four representative regulatory elements and a negative control (NC) site. Two elements were selected from regions with increased H3K27ac and two from regions with decreased H3K27ac, based on ChIP–seq results. Data are shown for two biological replicates (Rep1 and Rep2). Source data are provided as a Source Data file.

d Bar plots of the number of regulatory elements annotated as promoter or enhancer by ChromHMM. Regulatory elements were categorized as in Fig. 6e (increased, unchanged, or decreased).

e Heatmaps of BRD4 and MED1 ChIP-seq, alongside mNET-seq and TT-seq signals in control and auxin-treated cells at regulatory elements. Regulatory elements are shown in the same order as in Fig. 6a.

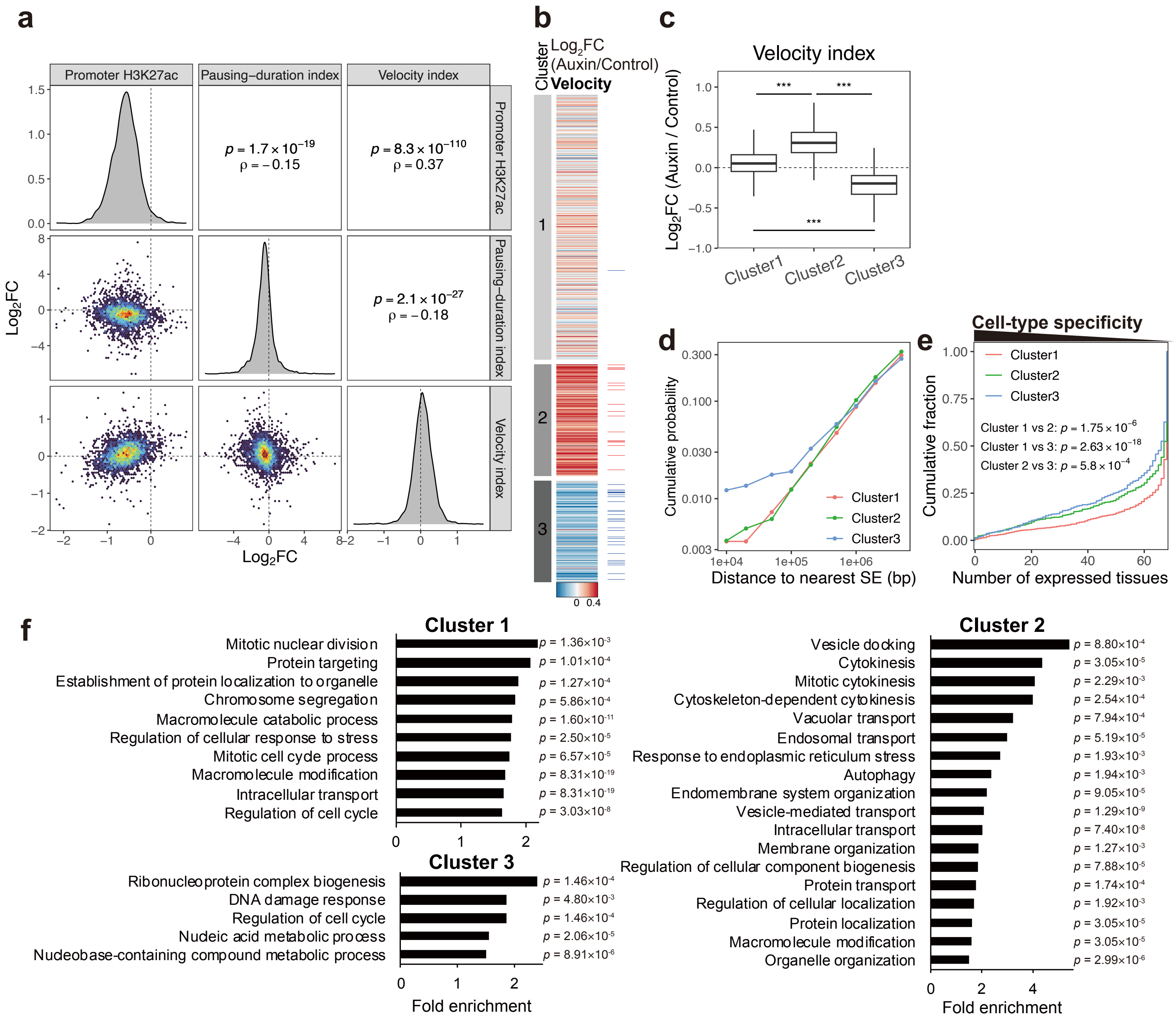

Supplementary Figure 9: Genes with modest expression changes upon cohesin loss show reduced Pol II recruitment and enhanced pause release.

a Pair plots showing the relationships among $\log_2$ fold changes in promoter H3K27ac, pausing-duration index, and velocity index. Velocity index was calculated as the TT-seq/mNET-seq ratio across gene bodies (TSS +1 kb to 3'end –1 kb). Diagonal panels show the distribution of each variable. Lower triangular panels show pairwise comparisons, and upper triangular panels display P-value (p) and Spearman's correlation coefficient (ρ) from two-sided Spearman's rank correlation test. No multiple-comparison adjustment was applied.

b Heatmaps showing $\log_2$ fold changes (auxin/control) in velocity index for each gene. Genes are shown in the same order and cluster assignment as in Fig. 7a. Genes identified as upregulated or downregulated by TT-seq (Fig. 1a) are highlighted with red and blue lines, respectively.

c Box plots showing $\log_2$ fold changes (auxin/control) in the velocity index across Clusters 1, 2, and 3 shown in Fig. 7a. Statistical significance was calculated using two-sided pairwise Welch's t-tests between gene groups (***$p < 0.001$). No multiple-comparison adjustment was applied. Exact P-values for velocity index: 2.13×10^{-190} (Cluster1 vs 2), 2.89×10^{-192} (Cluster1 vs 3), 3.41×10^{-232} (Cluster2 vs 3). Box plots show the median (centre), 25th and 75th percentiles (box bounds), and whiskers extending to the smallest and largest values within 1.5× the interquartile range from the quartiles.

d Cumulative probability distributions of distances to the nearest super enhancers for genes clustered into Cluster 1–3.

e Cumulative distributions of cell-type specificity for genes clustered into Cluster 1–3. Statistical significance was calculated using the Kolmogorov–Smirnov test.

f GO term analysis of genes clustered into Cluster 1–3. Only GO terms with a fold enrichment greater than 1.5 and a Benjamini-adjusted P value below 0.05 were considered significant and are shown.

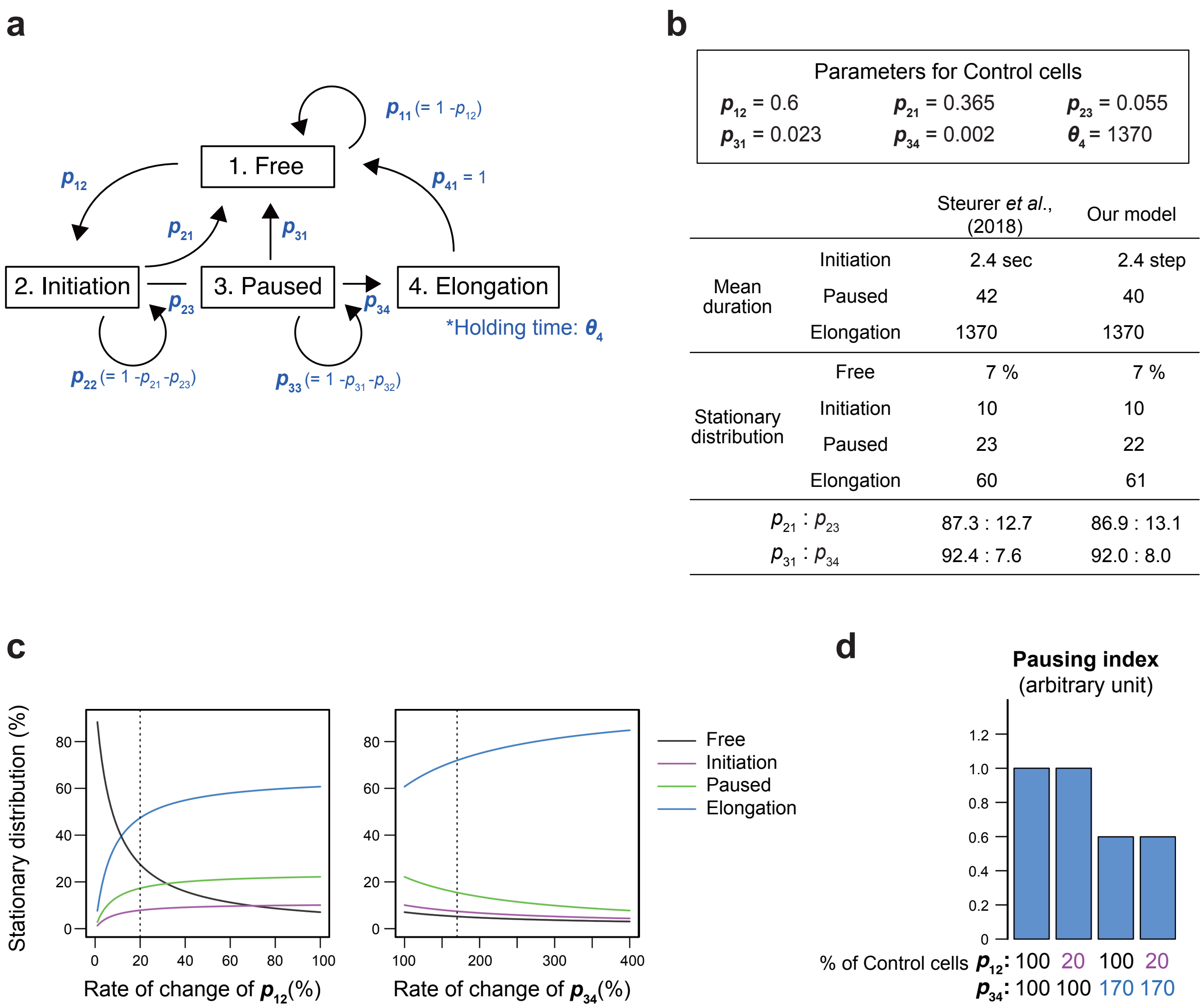

Supplementary Figure 10: Simulations suggest compensatory effects of reduced Pol II recruitment and enhanced pause release.
a Schematic of the transcriptional states and transitions in our model. Pol II moves through four states: Free (1), Initiation (2), Paused (3), and Elongation (4). Transitions between these states are governed by probabilities p_{ij}, where p_{ij} denotes the probability of transition from state i to state j. The holding time for the Elongation state is denoted by θ_4. The model includes six independent parameters.

b Estimated parameters and model validation. (Upper) Optimized parameters that best matched the intracellular Pol II dynamics. (Lower) Comparison of key indicators of Pol II dynamics between experimentally inferred values (Steurer et al., 2018) and our model's predictions using these optimized parameters.

c Line plots showing the impact of varying p_{12} (0-100%) and p_{34} (100-400%) on the stationary distribution. The vertical dotted line indicates the parameter set used in Figure S9D, representing the condition under cohesin depletion.

d Bar plots showing simulated effects of cohesin depletion on the pausing index. Our experimental data revealed that cohesin depletion reduces transcription initiation and enhances pause release. In our model, these changes correspond to decreased p_{12} and increased p_{34}, and each parameter is altered individually or in combination. The pausing index, designed to mimic the Pol II ChIP-seq pausing index, was calculated as ([I] + [P])/[E], where [X] represents the probability of Pol II being in state X. The values were normalized to 1 for the control condition.

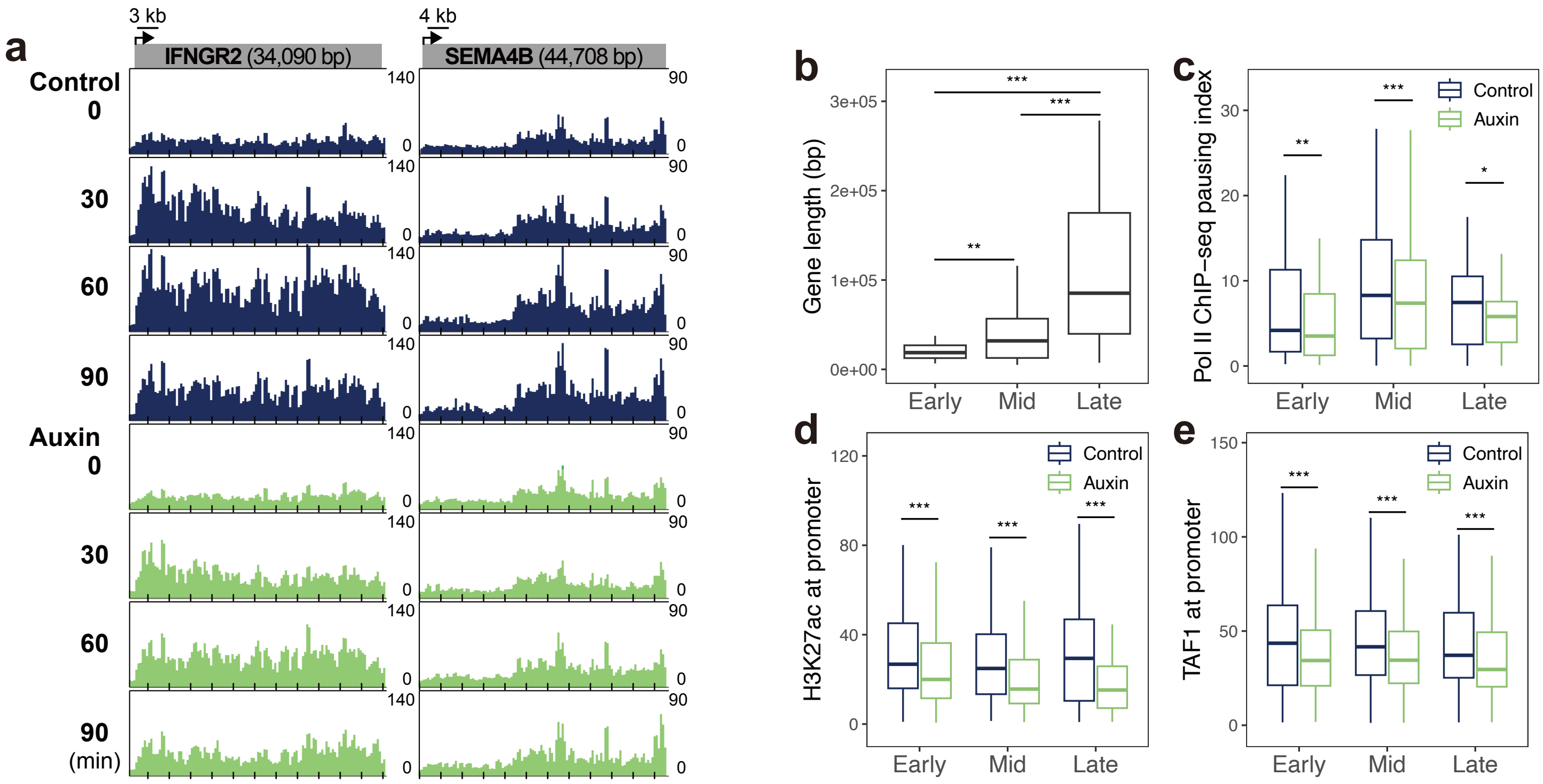

Supplementary Figure 11: Cohesin depletion impairs rapid gene induction.

a Representative genes showing TNFα-induced expression that was reduced by auxin treatment. EU-seq was performed in control and auxin-treated cells at 0, 30, 60, and 90 min after TNFα treatment.

b Box plots showing gene lengths of early-, mid-, and late-induced genes identified in Fig. 7e. Statistical significance was calculated using two-sided pairwise Welch's t-tests between gene groups (****p* < 0.001). No multiple-comparison adjustment was applied. Exact P-values: 7.35×10^{-3} (Early vs Mid), 4.80×10^{-6} (Early vs Late), 4.07×10^{-4} (Mid vs Late).

c Box plots showing Pol II ChIP-seq pausing index for early-, mid-, and late-induced genes in control and auxin-treated cells. Statistical significance was calculated using a two-sided Wilcoxon signed-rank test (**p* < 0.05, ***p* < 0.01, ****p* < 0.001). Exact P-values: 1.73×10^{-3} (Early), 4.21×10^{-5} (Mid), 1.18×10^{-2} (Late).

d Box plots showing promoter H3K27ac (TSS -1 kb to +1 kb) for early-, mid-, and late-induced genes in control and auxin-treated cells. Statistical significance was calculated using a two-sided Wilcoxon signed-rank test (****p* < 0.001). Exact P-values: 2.87×10^{-5} (Early), 7.34×10^{-11} (Mid), 9.31×10^{-10} (Late).

e Box plots showing promoter TAF1 (TSS -1 kb to +1 kb) at early-, mid-, and late-induced genes in control and auxin-treated cells. Statistical significance was calculated using a two-sided Wilcoxon signed-rank test (****p* < 0.001). Exact P-values: 3.37×10^{-5} (Early), 6.91×10^{-10} (Mid), 2.66×10^{-6} (Late).

All box plots show the median (centre), 25th and 75th percentiles (box bounds), and whiskers extending to the smallest and largest values within 1.5× the interquartile range from the quartiles.

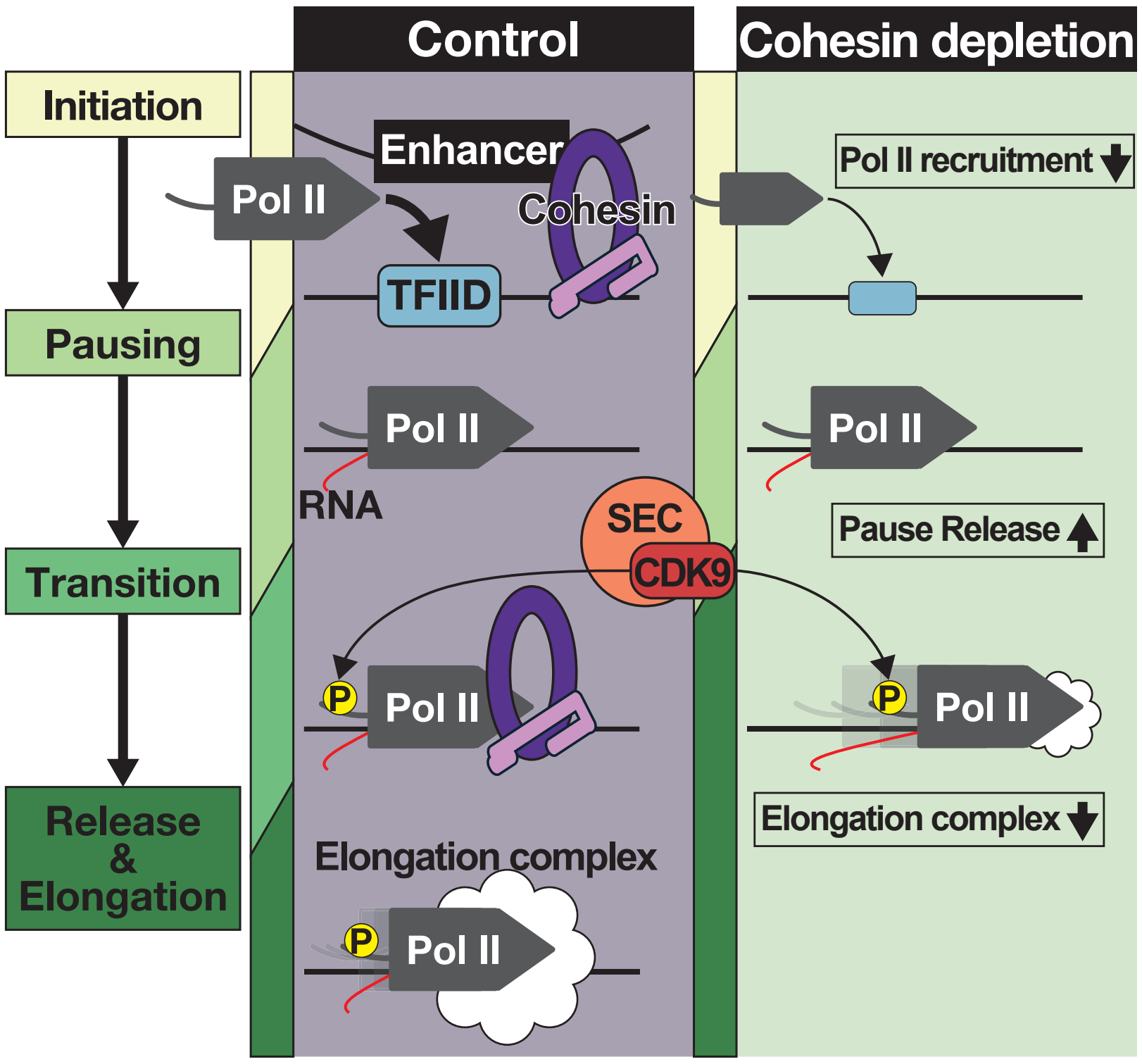

Supplementary Figure 12: Schematic model showing how cohesin regulates transcription.
Cohesin promotes Pol II recruitment by facilitating promoter–enhancer communication. It also restrains pause release by engaging the transcriptional machinery at the pausing-to-release transition. This engagement functions as a quality-control-like step that coordinates elongation factor recruitment and prevents premature transcription termination. Upon cohesin depletion, Pol II recruitment decreases while pause release is enhanced. These compensatory effects contribute to the minimal changes in steady-state gene expression. However, shortened pausing duration leads to immature formation of the elongation complex and premature transcription termination.

Supplementary Methods

Cell culture

HCT116 CMV-OsTIR1 SMC1A-mAID and HCT116 Tet-OsTIR1 Stag-3FLAG-NIPBL cells were generated as previously described[1,2]. Briefly, HCT116 CMV-OsTIR1 cells were co-transfected with SMC1A CRISPR and SMC1A-mAID-Hygro donor vectors, and HCT116 Tet-OsTIR1 cells with NIPBL-N CRISPR and Hygro-P2A-Stag-3FLAG-NIPBL donor vectors. After selection with 100 μg/mL HygroGold (Invivogen), single colonies were isolated, and successful knock-in was confirmed by genomic PCR and western blotting. SMC1 was depleted by treatment with 0.5 mM IAA for 3 h in HCT116 CMV-OsTIR1 SMC1A-mAID cells.

siRNA-mediated knockdown

RNA interference was performed with Lipofectamine RNAiMax (Invitrogen) using siRNA duplexes (Invitrogen) at a final RNA concentration of 50 nM, according to the manufacturer's instructions. Cells were collected two days after transfection. The siRNA sequences (5'→3') were: WAPL, UCUCCUGCUCGUUAGAAGUAAGGG; XRN2, UCGCUAUUACAUAGCUGAUCGUUUA.

Cell cycle profile analysis

Cells were fixed in 70% ethanol, washed with PBS, treated with RNase A (50 μg/mL), and stained with propidium iodide (50 μg/mL). DNA content was measured on BD Accuri C6 plus flow cytometer.

Quantitative PCR

ChIP DNA was quantified using StepOnePlus real-time PCR systems (Applied Biosystems) with KAPA SYBR Fast qPCR kit (KAPA Biosystems). The following primers were purchased from FASMAC and used (5'→3'): Rad21_1 F: GGCCTAGAAAACACGTCTACAG, R: TCCACCAAGGGACAAACAGAG; Rad21_2 F:

TGCGGGTACACTTTGAGAACC, R: GTGCCACCTAGTGGTATTTCCT; Rad21_3 F: CTTTGCCCCTTTGTGGCTCA, R: ATCCTGACACTGCTGGGAATG; negative control F: ATGAGTCTGGGATTTGGCTGG, R: TCTCAGGGGAAGGAGAGAGTC.

Purification of recombinant proteins

NIPBL mutants N1 (1–700) and N2 (701–1000) were N-terminally FLAG-tagged and subcloned into the pFASTBac HTB vector by GenScript. NIPBL mutant C (1121–2804) was C-terminally FLAG-tagged and subcloned into the pAcHLT-B vector, also by GenScript. Wild-type MAU2 was subcloned into the pAcSG2 vector by GenScript. Recombinant baculoviruses were generated using either the flashBAC Baculovirus Expression System (Mirus) or the Bac-to-Bac Baculovirus Expression System (Gibco), according to the manufacturer's protocols. NIPBL fragments (N2 and C), as well as N1 co-expressed with MAU2, were expressed in Sf9 cells following infection with recombinant baculoviruses. Cells were lysed using a Dounce homogenizer in PB500 buffer [25 mM NaH_2PO_4/Na_2HPO_4 (pH 7.5), 500 mM NaCl, 5% glycerol] supplemented with 10 mM imidazole, 0.05% Tween-20, 0.1% NP-40, 1 mM PMSF, 3 mM 2-mercaptoethanol, and 1x cOmplete EDTA-free (Roche). Lysates were clarified by centrifugation at 35,000 × g for 1 hour and incubated with Ni-NTA Agasore (Qiagen) for 2 hours at 4 °C. The resin was washed with PB500 supplemented with 20 mM imidazole, and bound proteins were eluted with PB150 (same composition as PB500 but with 150 mM NaCl) supplemented with 300 mM imidazole. Eluted proteins were further purified by incubation with ANTI-FLAG M2 Affinity Gel (Sigma) for 2 hours at 4 °C, washed with PB150, and eluted with PB150 containing 0.5 mg/ml 3×FLAG peptide. Final protein preparations were concentrated and buffer-exchanged into 20 mM Tris-HCl (pH 7.5), 100 mM NaCl, and 20% glycerol using Amicon Ultra Centrifugal Filters, 50kDa MWCO (Millipore).

SDS-PAGE, CBB staining, and western blotting

Protein samples were resolved by SDS–PAGE using either 4–15% Mini-PROTEAN® TGX™ Precast Protein Gels (Bio-Rad) or Perfect NT precast gels (DRC). For Coomassie Brilliant Blue (CBB) staining, gels were stained with one-step CBB stain solution (BIO CRAFT). For western blotting, proteins were transferred to Amersham Protran 0.2 NC nitrocellulose Western blotting membranes (GE Healthcare). The membranes were blocked with 3% skim milk in PBS-T and incubated with primary antibodies for several hours or overnight. After washing with PBS-T, the membranes were incubated with HRP-conjugated secondary antibodies for 1 hour. Signals were detected using SuperSignal West Pico PLUS Chemiluminescent Substrate (Thermo Scientific). When required, membranes were stripped using Stripping Solution (Wako) and reprobed.
Original uncropped blots are provided in the Source Data File.

Spike-in ChIP-seq and MNase ChIP-seq alignment and data analysis

Reads were aligned to human (hg38) and mouse (mm10) genomes with Bowtie (version 1.2.3)[3]. Normalization factors were calculated from the ratio of mouse spike-in to human reads in ChIP (IP) and input (WCE) samples[4]. Redundant reads were removed, and read numbers were normalized to a spike-in control with parse2wig (version 3.7.2)[5]. Peak calling was performed with drompa_peakcall (version 3.7.2) in "PC_SHARP" mode[5]. Visualization was carried out with drompa+ PC_SHARP (version 1.17.2) and deepTools(version 3.5.1)[6,7].

RNA-seq alignment and data analysis

Reads were aligned to the human genome (hg38) using STAR (version 2.7.10a)[8]. Transcript abundance was quantified using RSEM (version 1.3.3)[9]. Differential expression analysis was performed with edgeR (version 4.2.2); FDR < 0.05)[10].

TT-seq alignment and data analysis

Reads were aligned to human (hg38) and Drosophila (dm6) genomes with STAR (version 2.7.1a). Mapped reads were divided into sense and anti-sense reads using samtools (version 1.15.1)[11]. Scale factors were calculated from a Drosophila gene-level count matrix generated with htseq-count (version 1.99.2) and normalized with DESeq2 (version 1.46.0)'s estimateSizeFactors function[12,13]. A read count table was generated for genes, with TT-seq reads counted using drompa3 (version 3.7.2)[5], and differential expression analysis was performed with edgeR with the likelihood ratio test (FDR < 0.05)[10].

EU-seq alignment and data analysis

Low-quality reads were removed with Trimmomatic (version 0.39)[14]. Reads were aligned to the Drosophila genome (dm6) using Bowtie2 (version 2.4.5) in "--very-sensitive-" mode, and the unmapped reads were aligned to the human genome (hg38)[15]. Scale factors were calculated from a Drosophila gene-level count matrix generated with htseq-count (version 1.99.2) and normalized with DESeq2's estimateSizeFactors function. Redundant reads were removed, and the remaining reads were normalized to a spike-in control with parse2wig. Differential expression analysis was performed with edgeR with the likelihood ratio test (FDR < 0.05)[10].

mNET-seq alignment and data analysis

Reads were aligned to human (hg38) and Drosophila (dm6) genomes with STAR. Post-processing steps, including read deduplication, RNA 3' end identification, removal of PCR internal priming, and exclusion of reads associated with co-transcriptional splicing, were performed as described by Prudêncio et al[16]. Scale factors were calculated from a Drosophila gene-level count matrix generated with htseq-count and normalized with DESeq2's estimateSizeFactors function. Mapped reads were divided into sense and anti-sense reads using samtools[11].

Micro-C alignment and data analysis

Reads were aligned to the human genome (hg38) with BWA-MEM (version 0.75)[17]. Ligation-junction detection, read sorting, PCR-duplicate removal, and pair-file generation were performed using Pairtools (version 0.3.0)[18]. Matrix files were generated from the pairs files using Juicer (version 1.22.01) at 5 kb, 2 kb, and 1 kb resolutions. Loops were identified using Juicer Tools HiCCUPS and Mustache (version 1.2.0), and loops detected by both methods were combined to generate a set of Micro-C loops [19,20]. TADs were identified using Juicer Tools Arrowhead at a 25 kb resolution. Loop intensity was quantified using Chromosight[21] (version 1.6.1). For visualization, contact maps were generated using HiCExplorer (version 3.7.2), and aggregate plots were created using coolpup.py (version 1.0.0)[22,23].

CUT&Tag alignment and data analysis

Reads were aligned to human (hg38) and E. coli genomes with Bowtie2 (version 2.5.3) using Churros (version 1.5.0)[24]. Normalization factors were calculated from the ratio of E. coli spike-in to human reads. Redundant reads were removed, and read counts were normalized by the normalization factors with parse2wig+. Visualization was carried out with drompa+ PC_SHARP and deepTools.

Identification of promoter and enhancer regions

Twelve chromatin states were defined in control and auxin-treated cells using ChromHMM (version 1.25)[25] with ChIP-seq datasets for histone modifications (H3K27ac, H3K4me1, H3K4me3, H3K36me3, H3K79me2, H3K4me2, H2Aub, H2Bub, H3K27me3, H3K9me3), chromatin-associated factors (CTCF, BRD4, MED1), and TT-seq signal, all obtained from both conditions. Chromatin segments enriched for H3K27ac, H3K4me3, BRD4, and MED1 were annotated as active promoters, while those enriched for H3K27ac, H3K4me1, BRD4, and MED1 were classified as active enhancers.

Identification of up- or down-regulated promoters and enhancers

Active promoters and enhancers defined by ChromHMM were merged, and H3K27ac signal intensity was compared between control and auxin-treated cells using a one-sided upper-tail binomial test with drompa_draw CI. FDRs were computed from p-values using p.adjust in R, and peaks with FDR < 0.05 were classified as increased or decreased based on signal changes.

Evaluation of reproducibility

Reproducibility between biological replicates was systematically assessed for TT-seq, RNA-seq, EU-seq, ChIP-seq, and mNET-seq. Reproducibility was evaluated by calculating Spearman correlations of genome-wide read coverage in 10-kb bins after excluding bins containing zero counts. For Micro-C, replicate reproducibility was quantified using QuASAR-Rep scores as implemented in 3DChromatin_ReplicateQC at 100-kb resolution[26]. All assays showed high reproducibility across conditions, with replicate correlations and QuASAR-Rep scores reported in Supplementary Tables 1and 2.

References

1. Natsume, T., Kiyomitsu, T., Saga, Y. & Kanemaki, M. T. Rapid Protein Depletion in Human Cells by Auxin-Inducible Degron Tagging with Short Homology Donors. *Cell Rep.* **15**, (2016).

2. Yesbolatova, A., Natsume, T., Hayashi, K. ichiro & Kanemaki, M. T. Generation of conditional auxin-inducible degron (AID) cells and tight control of degron-fused proteins using the degradation inhibitor auxinole. *Methods* **164–165**, (2019).

3. Langmead, B., Trapnell, C., Pop, M. & Salzberg, S. L. Ultrafast and memory-efficient alignment of short DNA sequences to the human genome. *Genome Biol.* **10**, (2009).

4. Sakata, T. *et al.* A common molecular mechanism underlying Cornelia de Lange and CHOPS

syndromes. *Curr. Biol.* **35**, 1353-1363.e5 (2025).

5. Nakato, R., Itoh, T. & Shirahige, K. DROMPA: Easy-to-handle peak calling and visualization software for the computational analysis and validation of ChIP-seq data. *Genes to Cells* **18**, (2013).

6. Nakato, R. & Sakata, T. Methods for ChIP-seq analysis: A practical workflow and advanced applications. *Methods* **187**, (2021).

7. Ramírez, F. *et al.* deepTools2: a next generation web server for deep-sequencing data analysis. *Nucleic Acids Res.* **44**, (2016).

8. Dobin, A. *et al.* STAR: Ultrafast universal RNA-seq aligner. *Bioinformatics* **29**, (2013).

9. Li, B. & Dewey, C. N. RSEM Li and Dewey, 2011. *BMC Bioinformatics* **12**, (2011).

10. Robinson, M. D., McCarthy, D. J. & Smyth, G. K. edgeR: A Bioconductor package for differential expression analysis of digital gene expression data. *Bioinformatics* **26**, (2009).

11. Li, H. *et al.* The Sequence Alignment/Map format and SAMtools. *Bioinformatics* **25**, (2009).

12. Anders, S., Pyl, P. T. & Huber, W. HTSeq-A Python framework to work with high-throughput sequencing data. *Bioinformatics* **31**, (2015).

13. Love, M. I., Huber, W. & Anders, S. Moderated estimation of fold change and dispersion for RNA-seq data with DESeq2. *Genome Biol.* **15**, (2014).

14. Bolger, A. M., Lohse, M. & Usadel, B. Trimmomatic: A flexible trimmer for Illumina sequence data. *Bioinformatics* **30**, (2014).

15. Langmead, B. & Salzberg, S. L. Fast gapped-read alignment with Bowtie 2. *Nat. Methods* **9**, (2012).

16. Prudêncio, P., Rebelo, K., Grosso, A. R., Martinho, R. G. & Carmo-Fonseca, M. Analysis of Mammalian Native Elongating Transcript sequencing (mNET-seq) high-throughput data. *Methods* **178**, (2020).

17. Li, H. & Durbin, R. Fast and accurate long-read alignment with Burrows-Wheeler transform.

Bioinformatics **26**, (2010).

18. Open2C *et al.* Pairtools: from sequencing data to chromosome contacts. *bioRxiv Prepr. Serv. Biol.* (2023). doi:10.1101/2023.02.13.528389

19. Durand, N. C. *et al.* Juicer Provides a One-Click System for Analyzing Loop-Resolution Hi-C Experiments. *Cell Syst.* **3**, (2016).

20. Roayaei Ardakany, A., Gezer, H. T., Lonardi, S. & Ay, F. Mustache: multi-scale detection of chromatin loops from Hi-C and Micro-C maps using scale-space representation. *Genome Biol.* **21**, 256 (2020).

21. Matthey-Doret, C. *et al.* Computer vision for pattern detection in chromosome contact maps. *Nat. Commun.* **11**, (2020).

22. Ramírez, F. *et al.* High-resolution TADs reveal DNA sequences underlying genome organization in flies. *Nat. Commun.* **9**, (2018).

23. Flyamer, I. M., Illingworth, R. S. & Bickmore, W. A. Coolpup.py: Versatile pile-up analysis of Hi-C data. *Bioinformatics* **36**, (2020).

24. Wang, J. & Nakato, R. Churros: a Docker-based pipeline for large-scale epigenomic analysis. *DNA Res.* **31**, (2024).

25. Ernst, J. & Kellis, M. Chromatin-state discovery and genome annotation with ChromHMM. *Nat. Protoc.* **12**, (2017).

26. Yardımcı, G. G. *et al.* Measuring the reproducibility and quality of Hi-C data. *Genome Biol.* **20**, (2019).

Supplementary Figure 1a

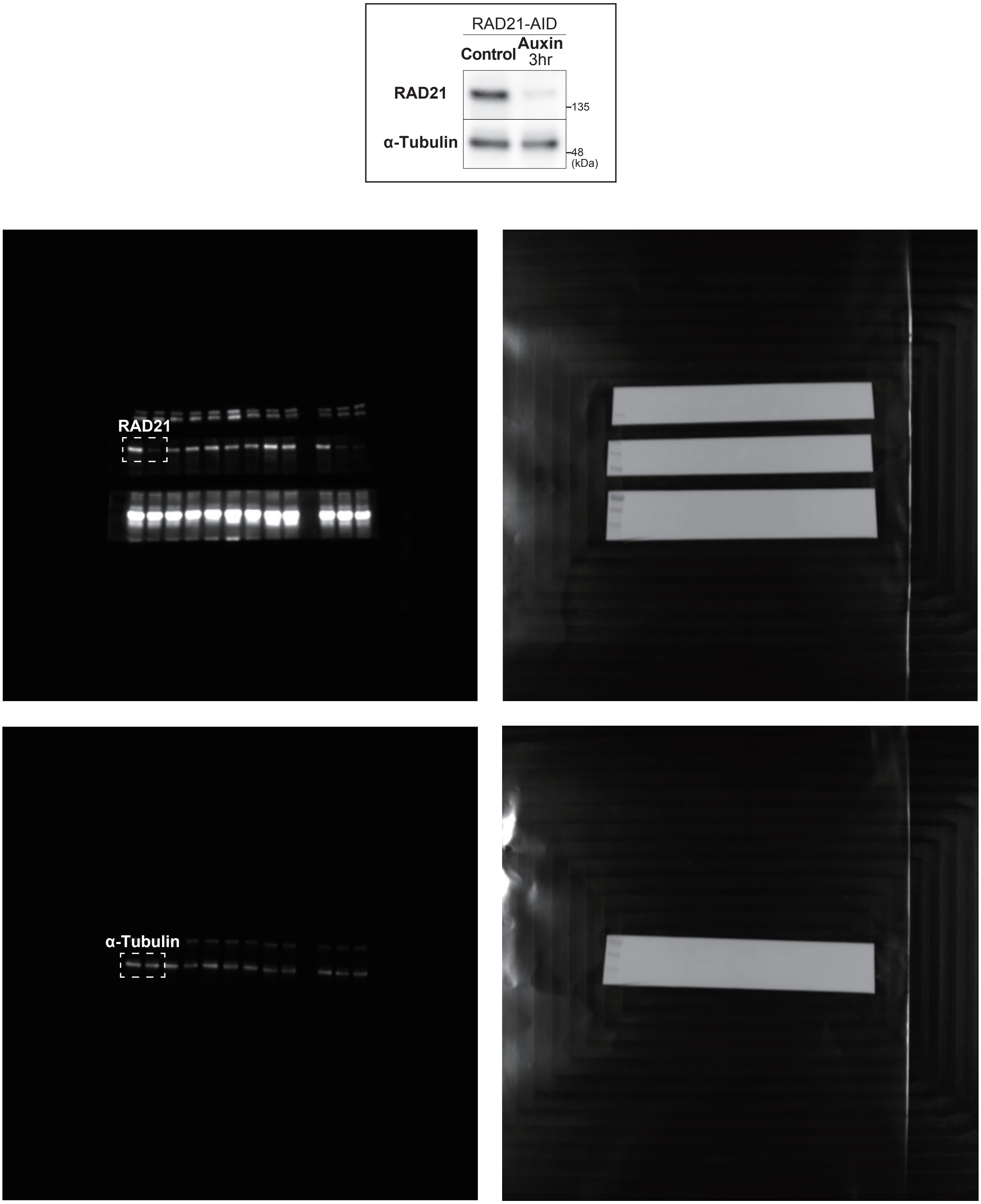

Target	Condition	signal	total	area	mean	median_ background	ROI_x	ROI_y	ROI_w	ROI_h	RAD21 / α-Tubulin	Relative RAD21 level
RAD21	Control	18360030	33516030	1800	18620.017	8420	0	4	45	40	0.992406	1
RAD21	Auxin	836700	13908356	1591	8741.896	8216	45	6	43	37	0.057026	0.057462
α-Tubulin	Control	18500515	33578675	1640	20474.802	9194	3	2	40	41		
α-Tubulin	Auxin	14672249	30426581	1681	18100.286	9372	46	2	41	41		

Supplementary Figure 2d

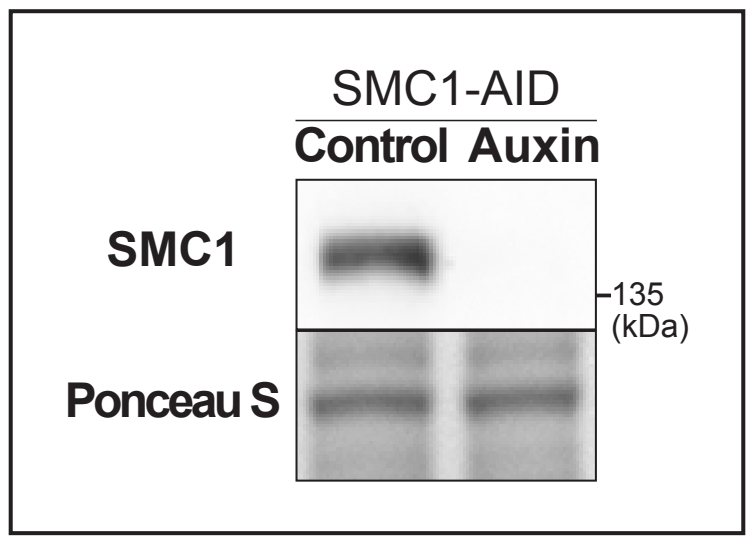

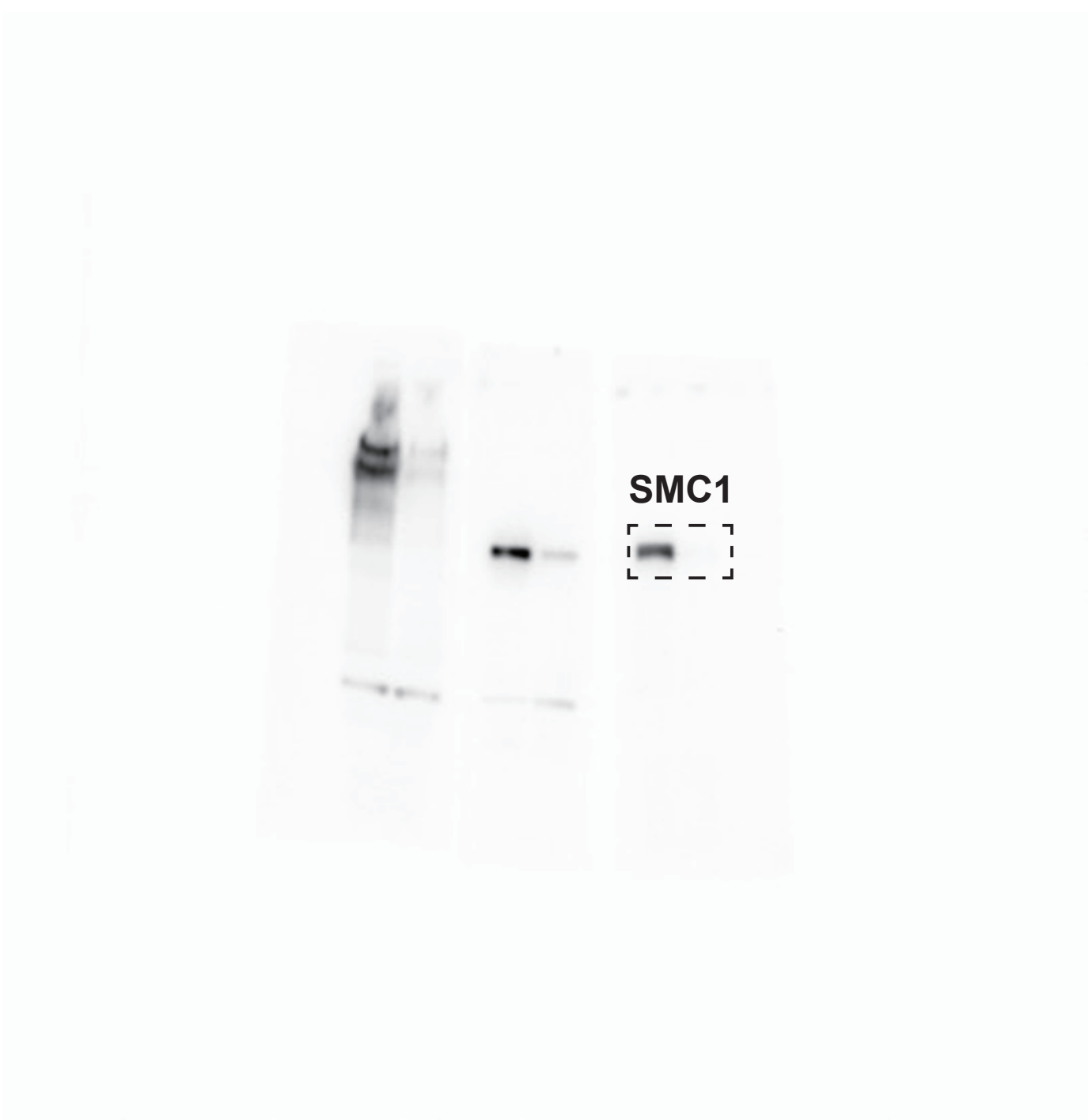

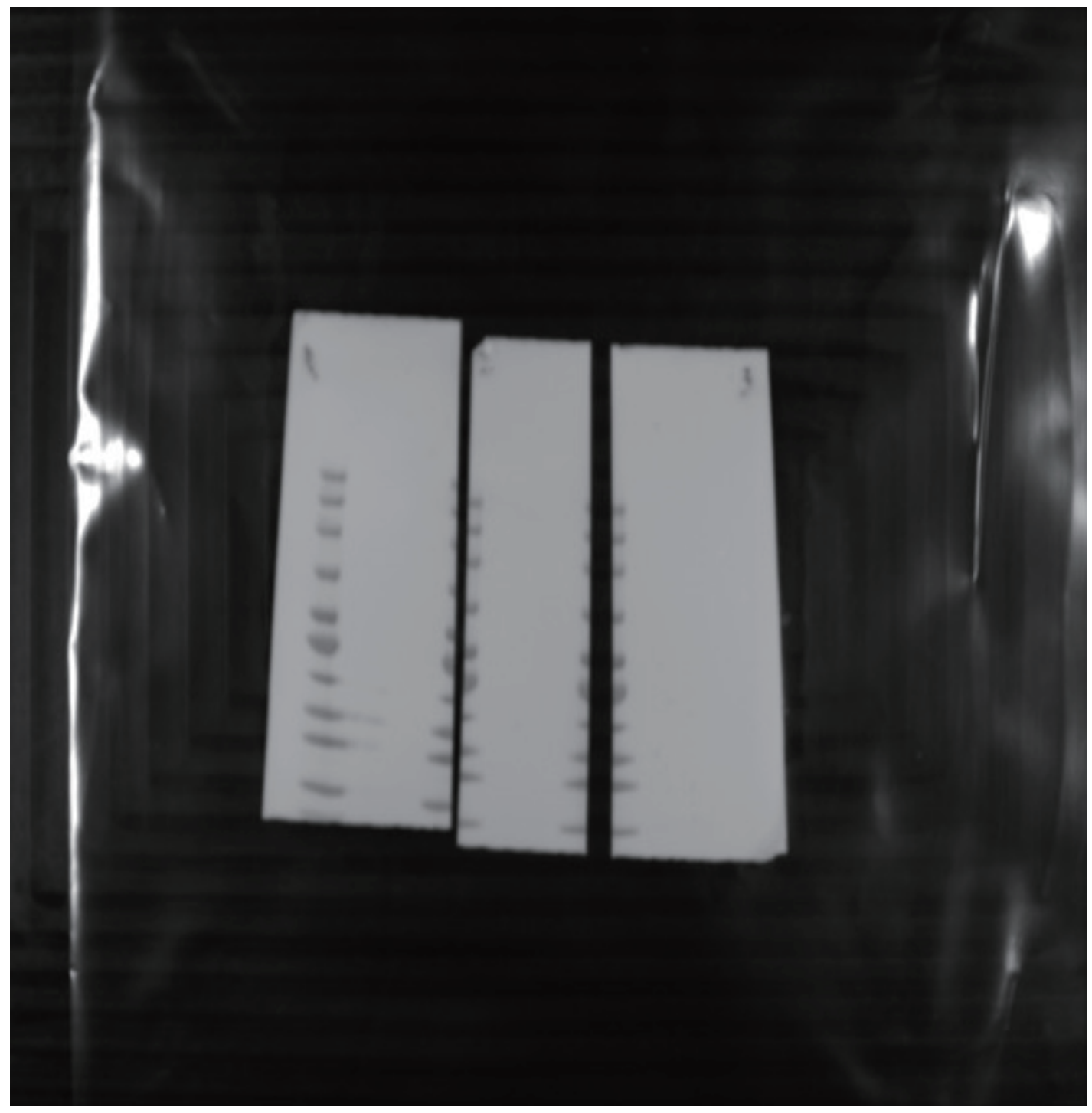

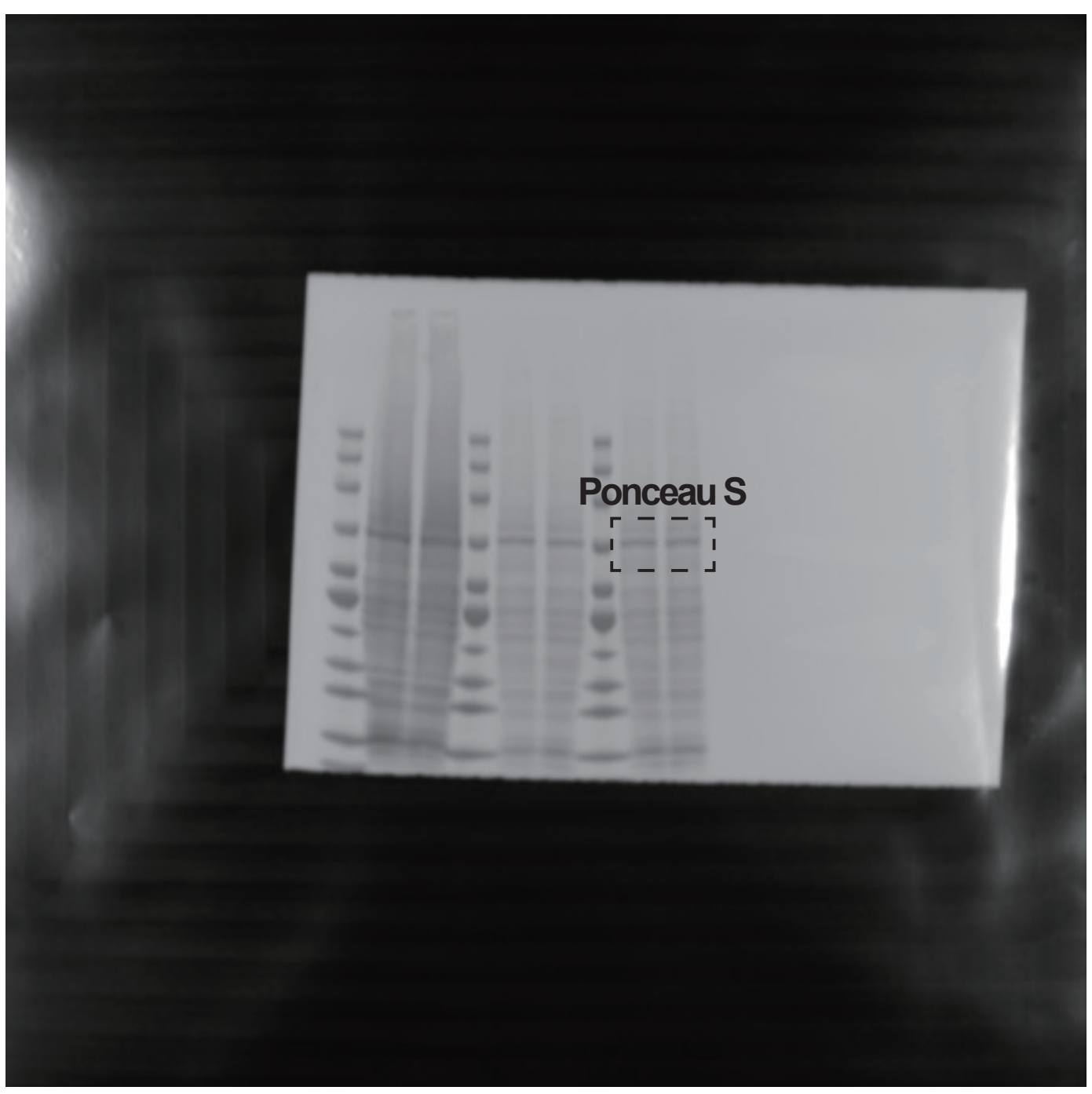

Target	Condition	signal	total	area	mean	median_background	ROI_x	ROI_y	ROI_w	ROI_h	SMC1 / Ponceau S	Relative SMC1 level
SMC1	Control	70438	80266	1638	49.002	6	3	5	42	39	5.313268	1
SMC1	Auxin	938	7658	1680	4.558	4	49	2	40	42	0.053305	0.010032
Ponceau S	Control	13257	90993	984	92.473	79	1	13	41	24		
Ponceau S	Auxin	17597	87461	984	88.883	71	47	10	41	24		

Supplementary Figure 3I

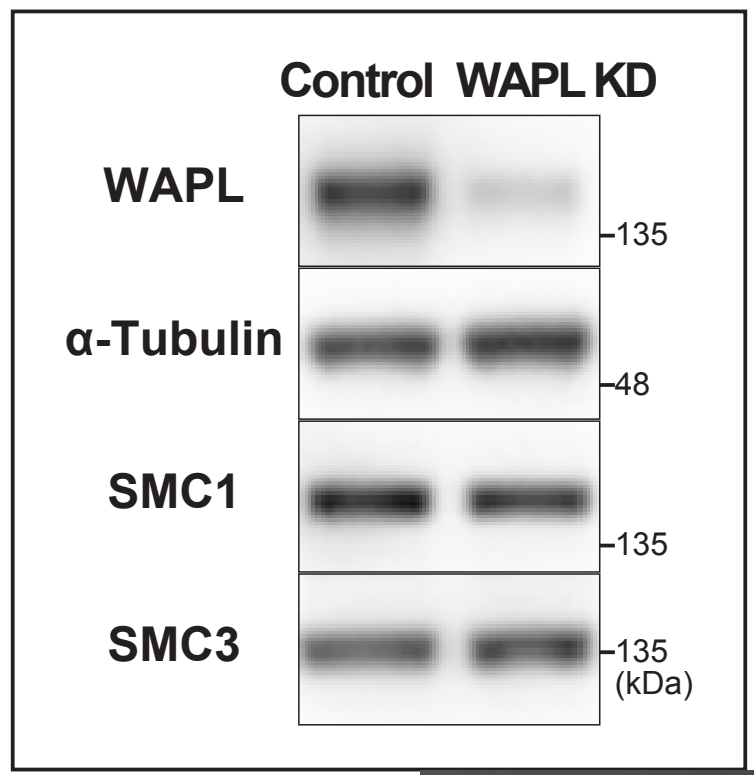

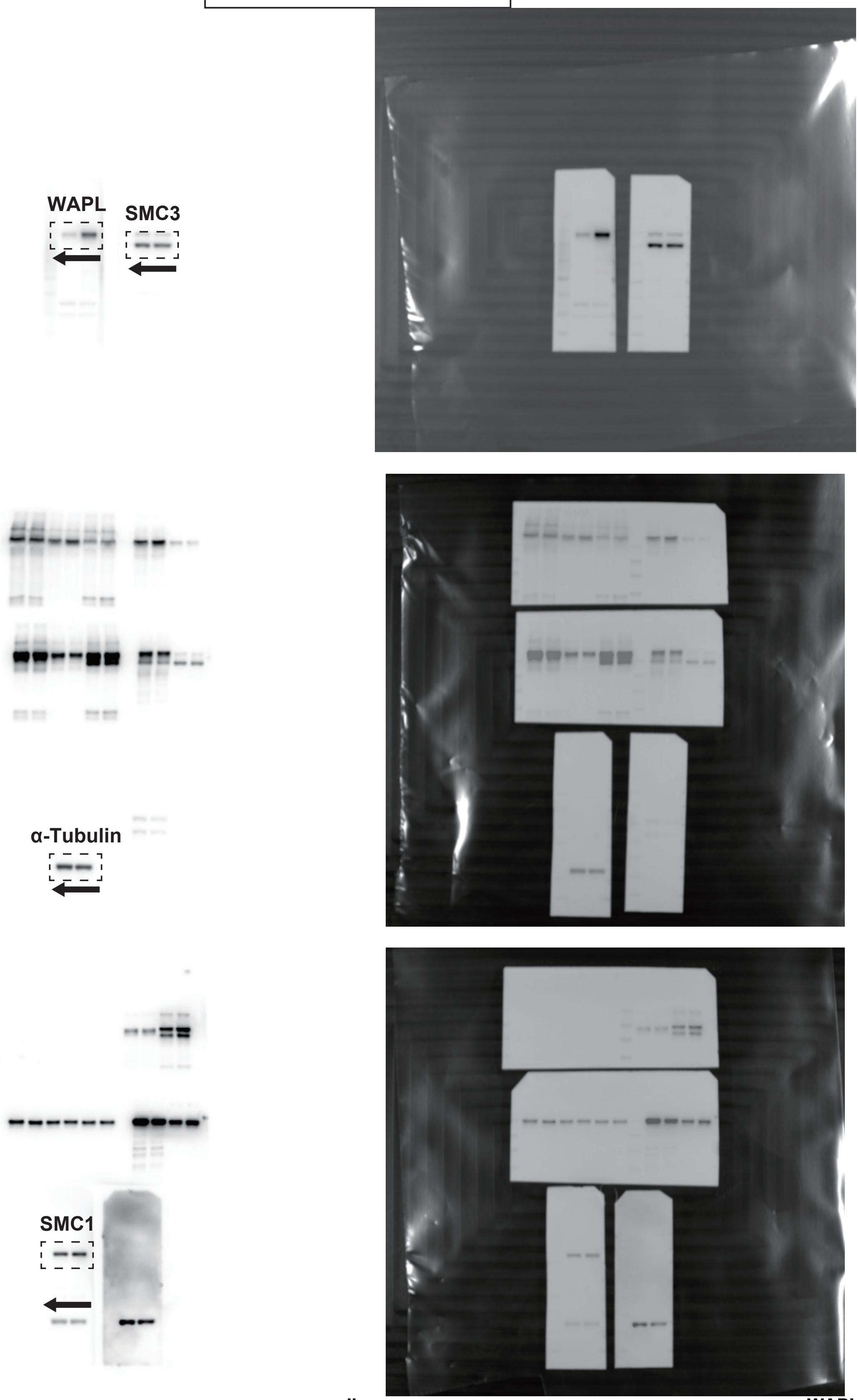

Target	Condition	signal	total	area	mean	median_ background	ROI_x	ROI_y	ROI_w	ROI_h	WAPL / α-Tubulin	Relative WAPL level
WAPL	Control	86117	122417	1452	84.309	25	45	7	44	33	1.131065	1
WAPL	WAPL KD	21601	34669	1452	23.877	9	1	7	44	33	0.252871	0.223569
α-Tubulin	Control	76138	85114	1496	56.894	6	45	7	44	34		
α-Tubulin	WAPL KD	85423	95895	1496	64.101	7	0	6	44	34		

Supplementary Figure 4f

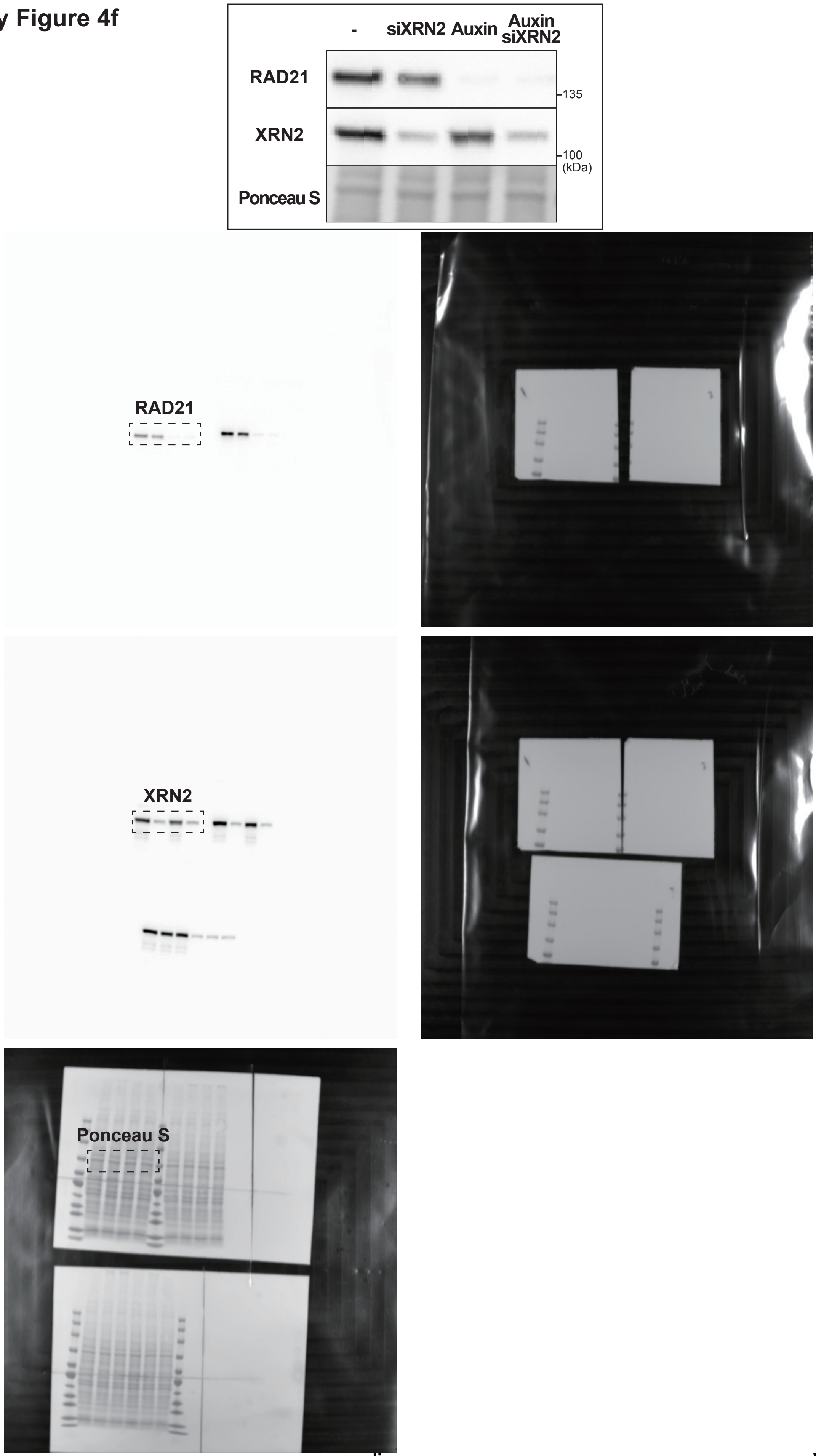

Condition	signal	total	area	mean	median_background	ROI_x	ROI_y	ROI_w	ROI_h	WAPL / α-Tubulin	Relative WAPL level
Control	76756	90556	1380	65.62	10	5	7	46	30	0.262160	1
siXRN2	16851	25131	1380	18.211	6	52	7	46	30	0.058298	0.222376
Auxin	58799	69839	1380	50.608	8	96	7	46	30	0.194299	0.741147
Auxin, siXRN2	17783	24683	1380	17.886	5	142	7	46	30	0.057783	0.220410
Control	17383	292783	4050	72.292	68	7	21	90	45		
siXRN2	90599	289049	4050	71.37	49	102	26	90	45		
Auxin	83921	302621	4050	74.721	54	195	27	90	45		
Auxin, siXRN2	44507	307757	4050	75.989	65	287	28	90	45		

Supplementary Figure 6d

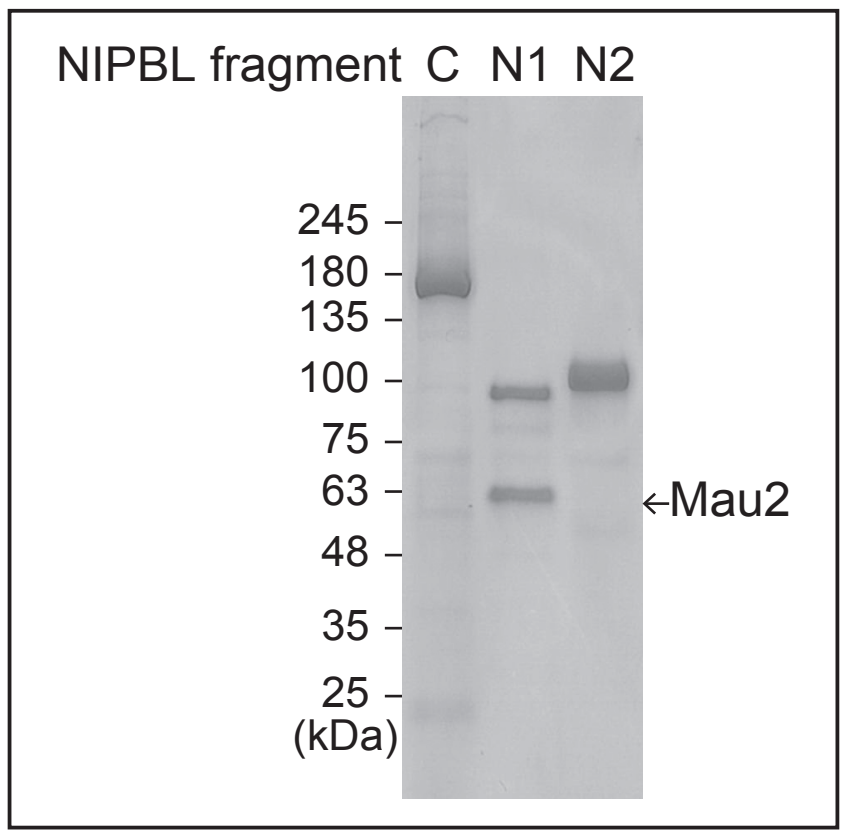

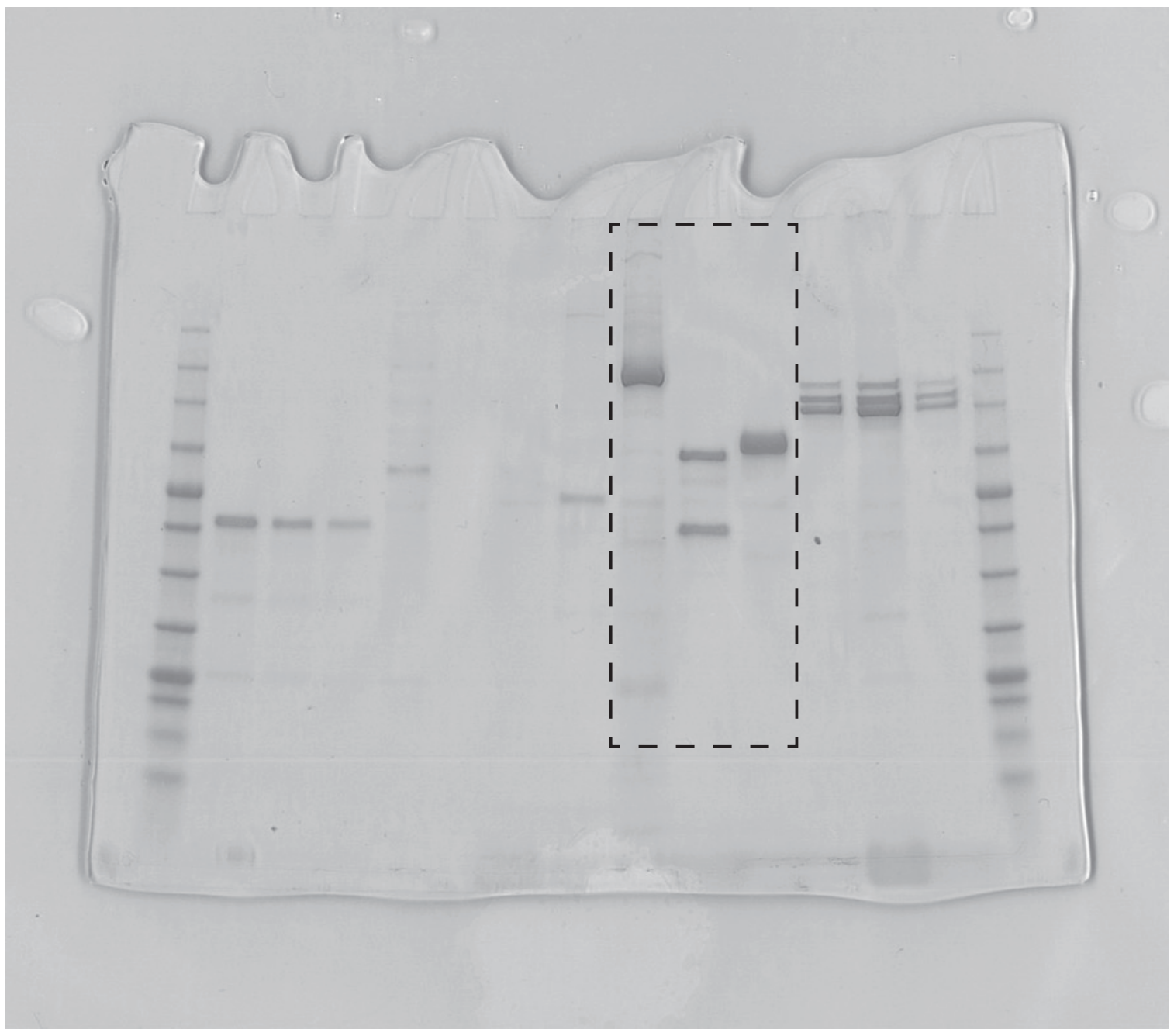

Supplementary Figure 6e

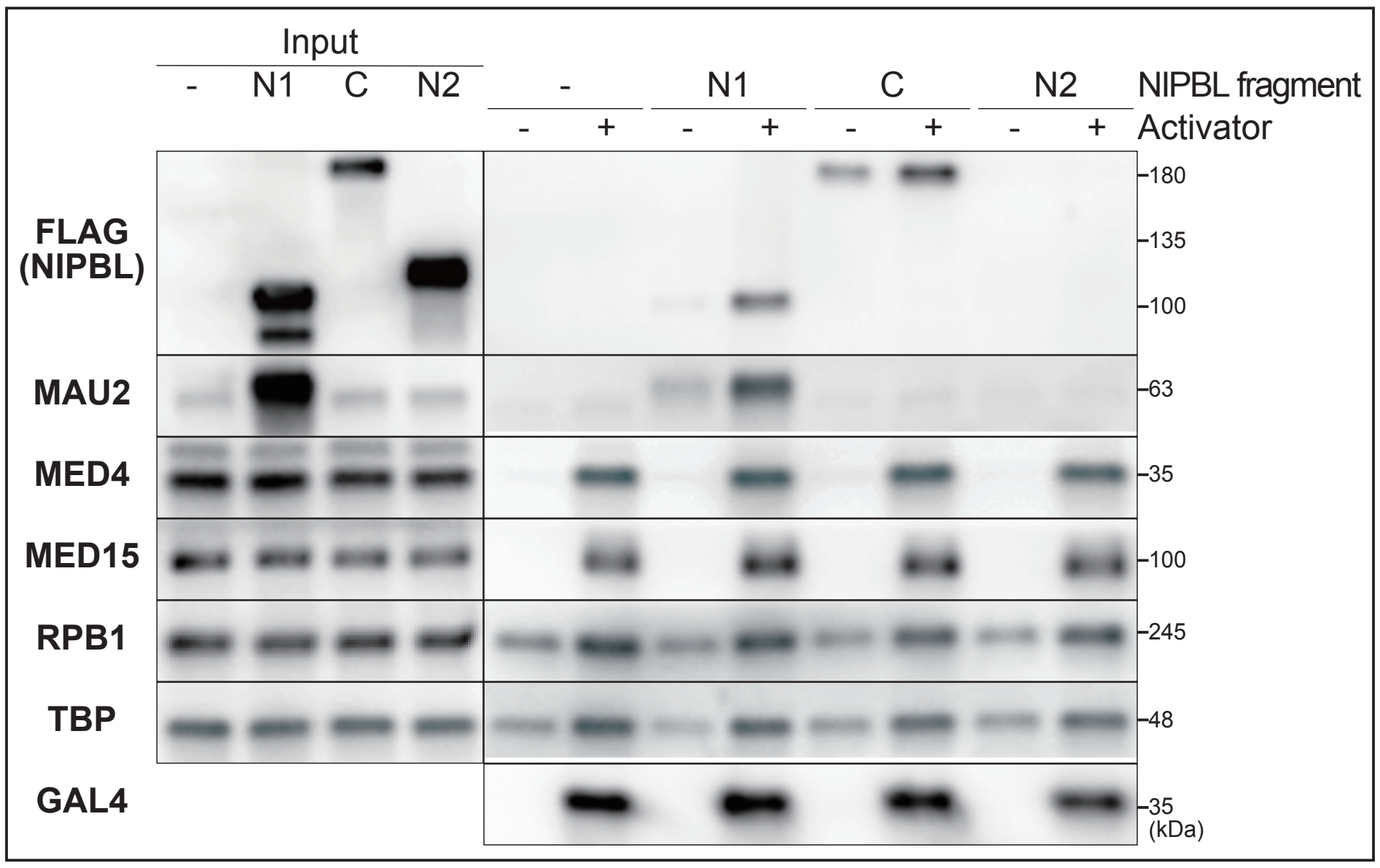

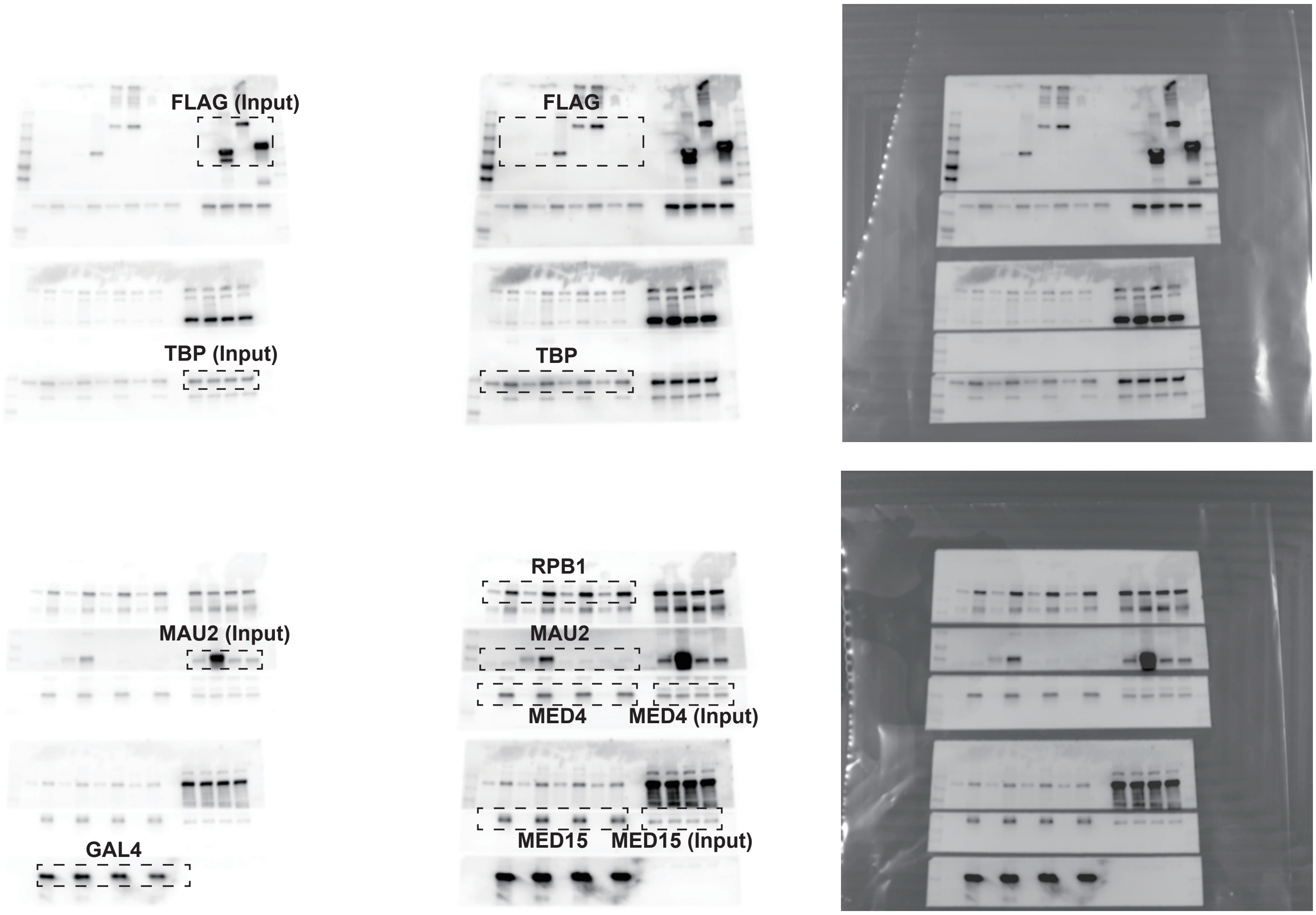

Supplementary Figure 6f

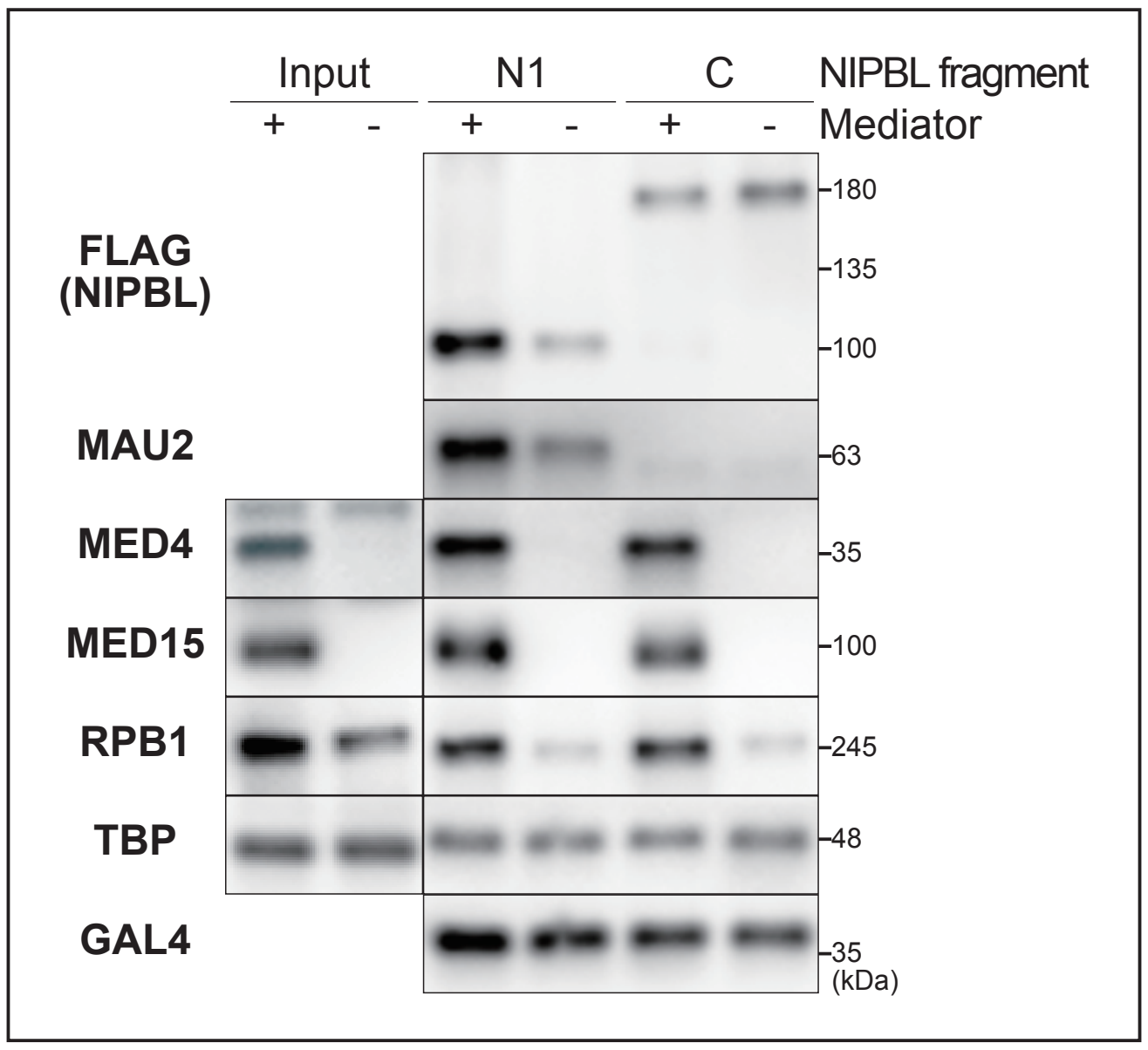

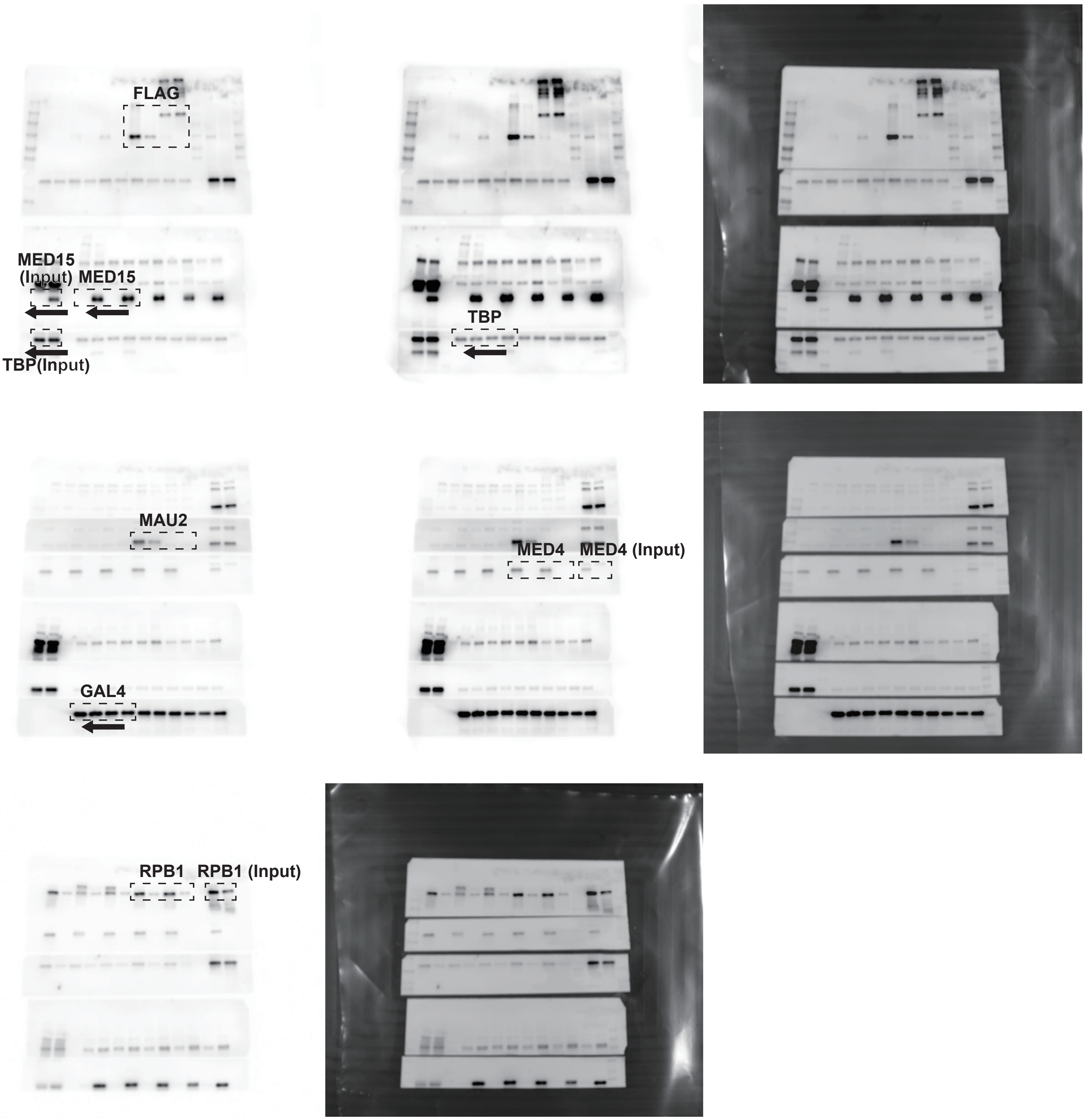

Supplementary Figure 6g

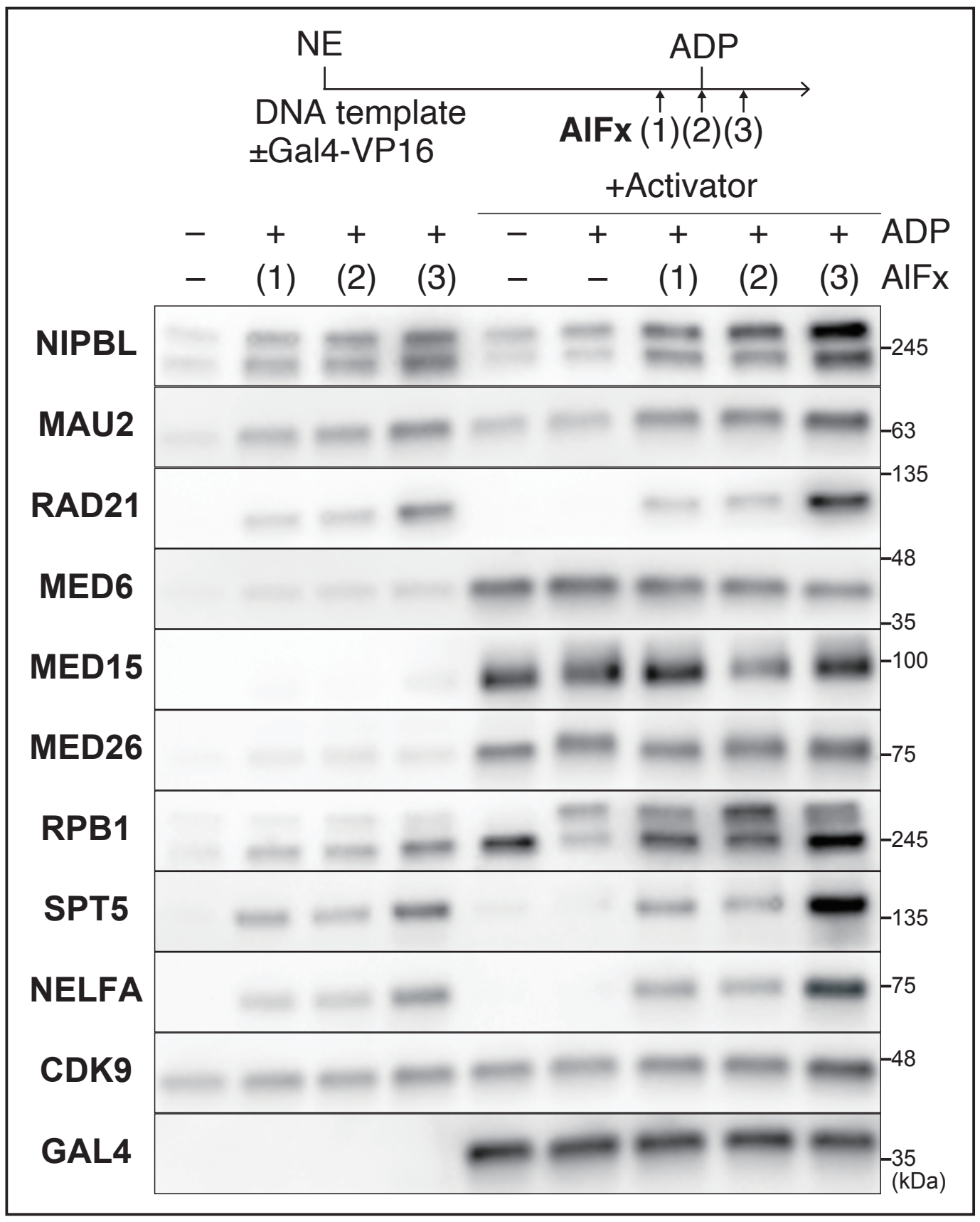

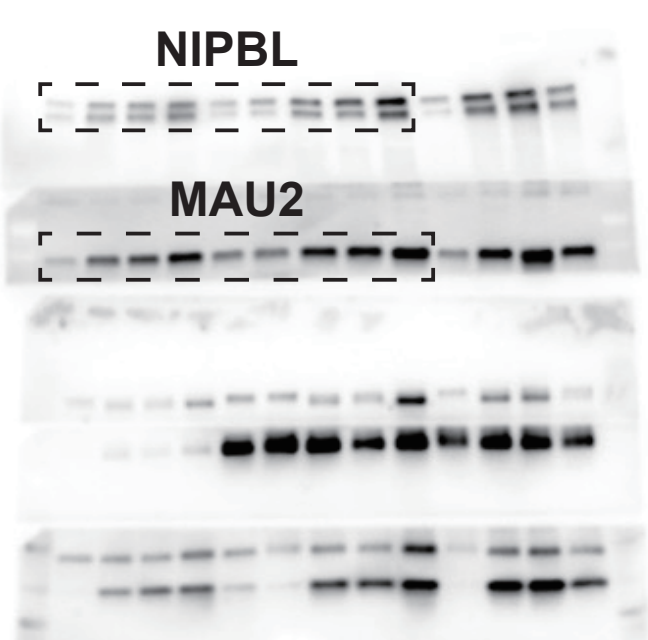

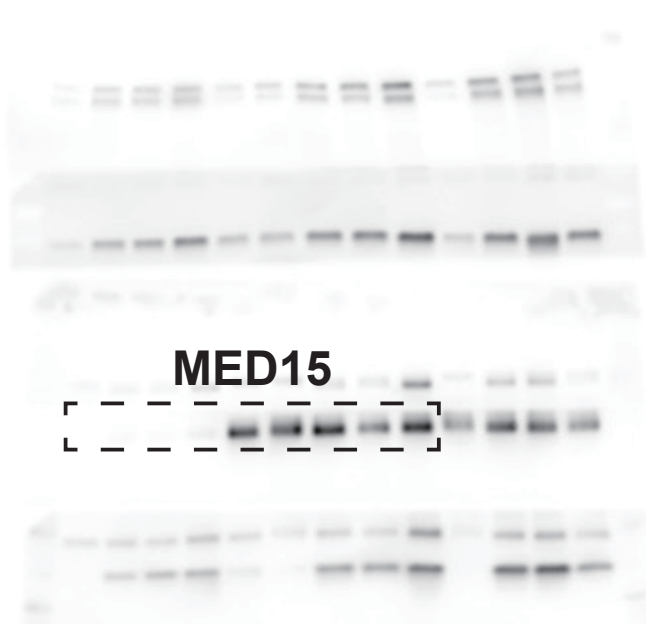

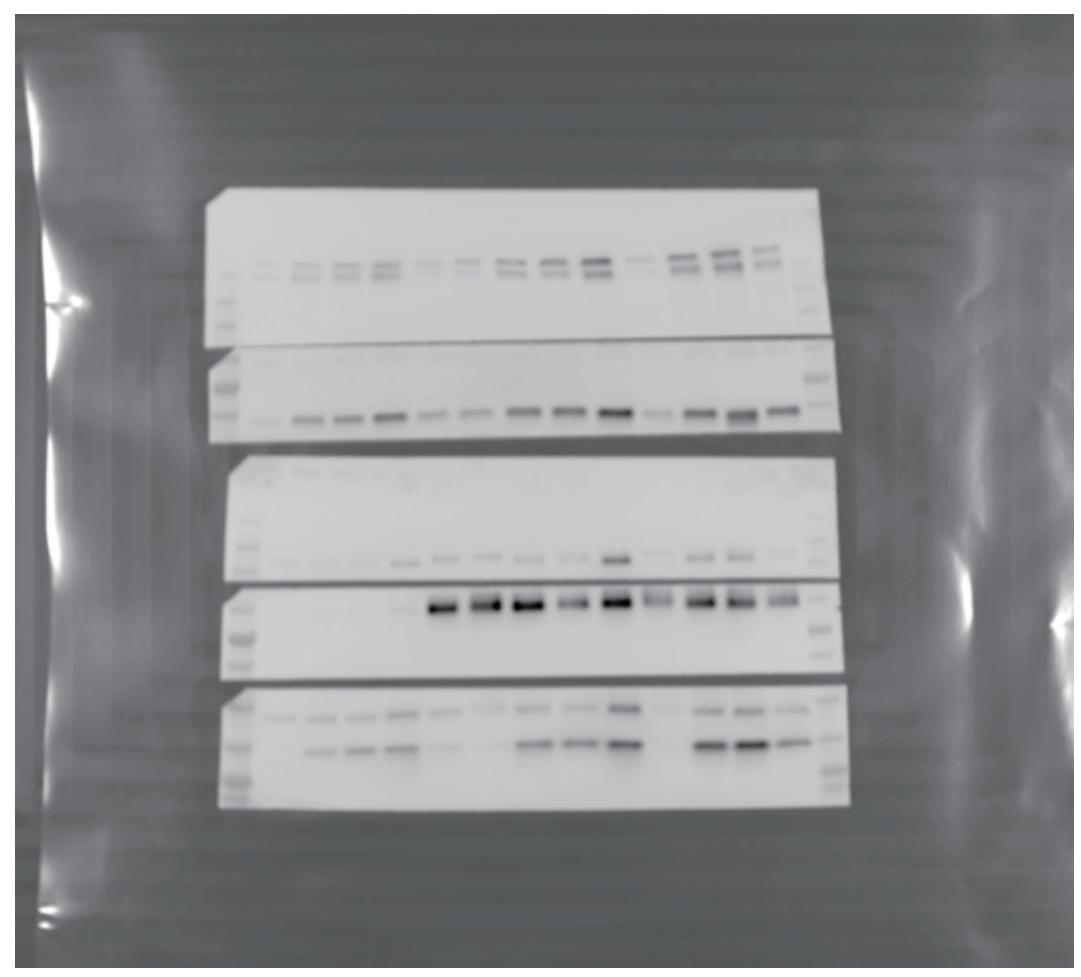

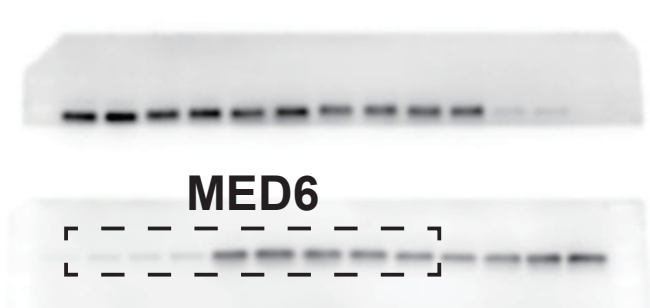

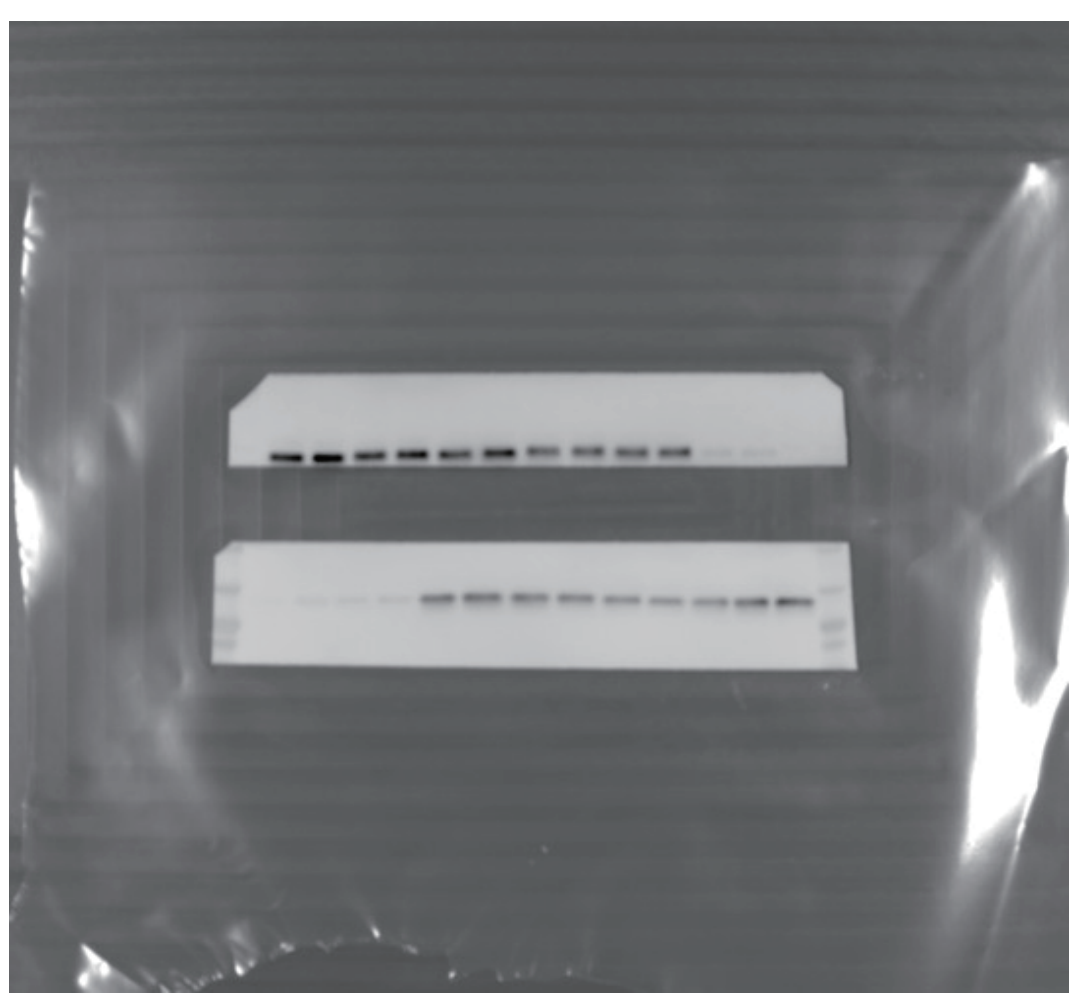

Supplementary Figure 6g

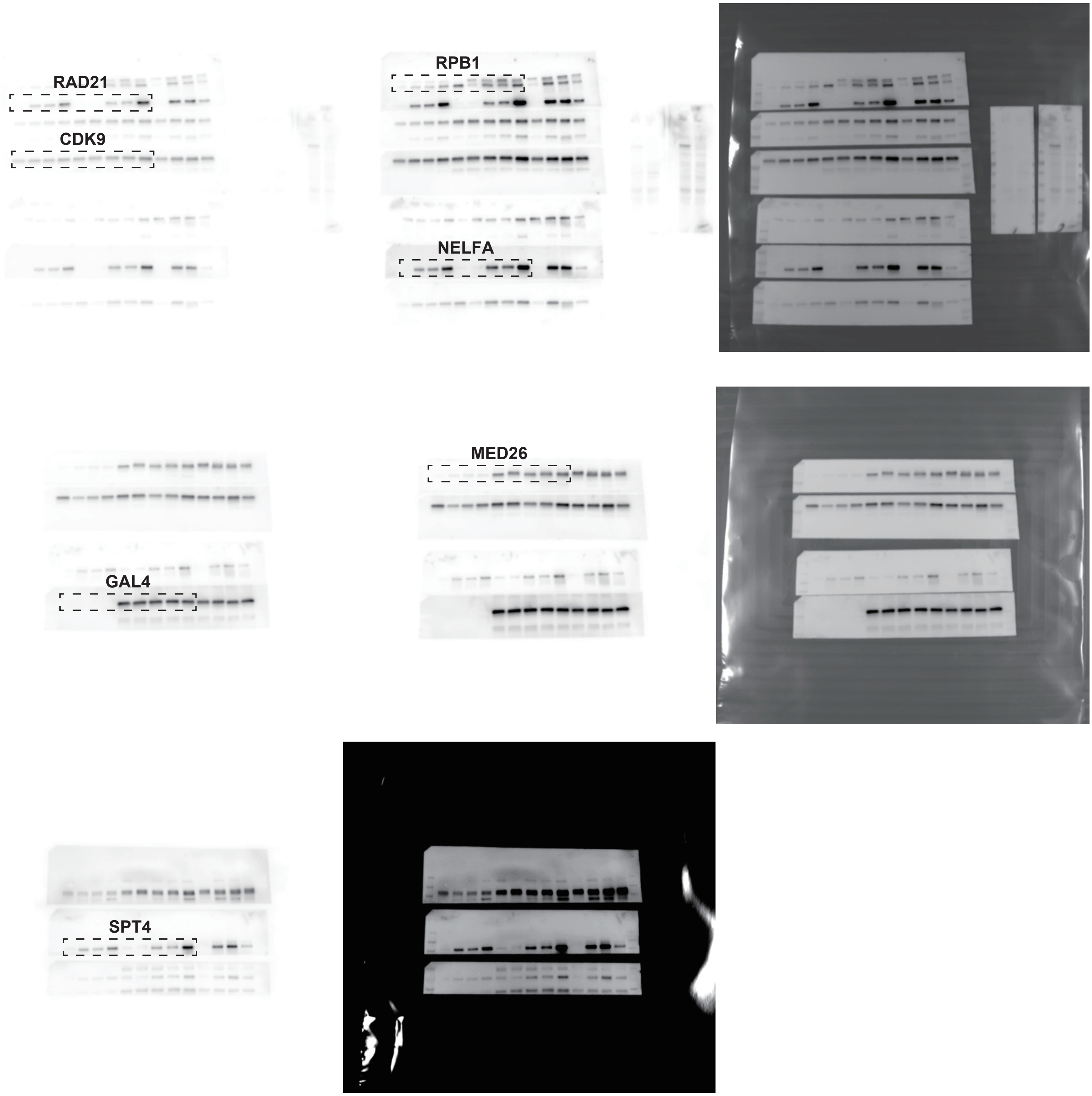

Supplement: Supplementary file 1 [file 41467_2026_76738_MOESM1_ESM.pdf]
