# Supplementary material for: Cohesin acts as a transcriptional gatekeeper by restraining pause–release to promote processive elongation
Source: Nat Commun. 2026 Aug 24;17:8487. doi: 10.1038/s41467-026-76738-3 (PMC13503787; doi:10.1038/s41467-026-76738-3)
Supplement: Supplementary file 2 [file 41467_2026_76738_MOESM2_ESM.pdf]

nature portfolio

Peer Review File

Cohesin acts as a transcriptional gatekeeper by restraining pause–release to promote processive elongation

Corresponding Author: Dr Katsuhiko Shirahige

This file contains all reviewer reports in order by version, followed by all author rebuttals in order by version.

Version 0:

Reviewer comments:

Reviewer #1

(Remarks to the Author)
In this important manuscript, Tei et al. solve one of the conundrums in the transcription regulation field. Cohesin is a key architect of higher order chromatin architecture, and mutations in cohesin and its regulators have stark clinical implications. Yet, acute cohesin depletion changes a cell's net transcriptional output only mildly. How cohesin therefore impacts on transcription remained unclear. The authors address this important question in a series of genomics and biochemical experiments. These are performed to a high experimental standard and the results support most of the authors conclusions (but see below). It emerges that cohesin plays two important roles in transcriptional regulation, promoter-proximal pausing and assembly of elongation-competent RNA pol II complexes. The two roles often cancel each other out, but not always. This insight and the resultant new way of looking at cohesin explains the conundrum why cohesin is so important, yet shows at first sight only subtle effects on net transcriptional output.

Additional noteworthy concepts arise during the course of the authors' study, e.g. that the right length of promoter-proximal pausing is vital for the assembly of a fully elongation-competent transcription machinery. Together I can highly recommend this manuscript for publication, after the authors have addressed the following shortcomings.

In the order of appearance, not importance:

1. Page 3: '…by briefly associating with the transcription machinery', how brief is 'brief'? Depending on the gene and transcriptional program, the pausing state can be extended. Maybe 'transiently', or 'reversibly' is a better term?

2. Page 4: '…decreasing short-range (<1 Mb) and increasing long-range contacts'. How do we know that long-range contacts increase? If cohesin engages in short-range contacts, then loss of these contacts results in a relative, but not an actual, increase in long-range contacts. The quantitative analysis of chromatin interactions remains an important challenge for the chromatin structure field, and it likely goes beyond the scope of the current study to solve this challenge. Until then, however, the authors should be careful with the interpretation of normalised interaction matrices.

3. Page 4: '…50 of which overlapped with those identified in RAD21-depleted cells'. The authors present what may be one of the most thorough analyses of net gene expression changes following cohesin depletion. Is there anything noteworthy about the list of 50 genes?

4. Page 5: 'Cohesin loss reduced this index,…' The authors should investigate whether this loss is statistically significant.

5. Page 6: 'Given the reduced pausing…', this paragraph is hard to read, despite of its importance. Can this paragraph be rewritten, ideally with less use of acronyms?

6. Page 6: '… velocity index – the TT-seq/mNET-seq ratio across gene bodies'. The velocity index appears to be the inverse of the pausing index. Can for simplicity the pausing index be used for this analysis? Or if not, can the relationship between the pausing and the velocity index be made clearer in the text?

7. Page 8: '…NIPBL-MAU2 heterodimer, is required not only for loading cohesin onto DNA but also for cohesin's translocation with loop formation'. I am unsure whether this statement is correct. Guérin et al. 2024 found that once cohesin

is loaded onto DNA and loops are formed, the translocation of cohesin and the growth of loops occurs independently of the cohesin loader.

8. Page 9: '… it is likely that cohesin also interacts with Pol II'. This conclusion is not supported by the data. The authors report that they did not see a direct interaction between cohesin and Pol II, and neither have others. It remains therefore unclear why the authors conclude the opposite? The more genuine conclusion is that the cohesin loader, but not cohesin, directly interact with Pol II. Cohesin in turn likely interacts with this assembly via the loader. The loader-cohesin interaction is known to be stabilized by ADP-AlFx, explaining how cohesin becomes a stable complex component under these conditions.

9. Related to the above, the experiment in Figure 4c suggests that cohesin is recruited to the transcription complex only in the presence of ADP-AlFx. However, might cohesin also be recruited to the transcription-loader complex in the absence of ADP-AlFx, but lost again following loading, either by sliding off the linear DNA template, or by ATP hydrolysis-dependent unloading, stimulated by the action of unloading factors such as Pds5-Wapl? Can the authors explore this possibility? It might be tricky to perform a conclusive experiment, but using a doubly tethered DNA or depletion of Wapl might be approaches that might yield insight.

10. Page 10: '… Cohesin's ability to entrap and translocate along DNA depends on its ATPase activity… (ref 36)'. This statement is likely incorrect (the reference points to a review that is peripheral to this statement). It is well established that, once loaded onto DNA, cohesin translocation requires no further ATPase activity. In vitro evidence can be found in several of the single molecule studies that observe cohesin translocation along stretched DNA following loading (most recently Glaser et al. 2025, Figure S1E and note Figure S7B). In vivo evidence is presented in Ocampo et al. 2016.

Reviewer #2

(Remarks to the Author)
Shoin Tei et al. present an intriguing explanation for the paradoxical observation that cohesin depletion leads to only limited changes in gene expression. By integrating mNET-seq, TT-seq, and in vitro biochemical assays, the authors propose a model wherein cohesin plays a dual role: it facilitates transcription initiation by promoting enhancer-promoter communication while also acting as a gatekeeper to restrain RNA polymerase II (Pol II) pause-release. The counterbalance between reduced initiation and enhanced pause-release following cohesin depletion is proposed to account for the minimal net effect on steady-state transcription. While this narrative is logically compelling and supported by careful investigation, the study would be strengthened by more direct genomic evidence to substantiate the model beyond computational inferences from high-throughput datasets.

Specific points for consideration include:
1. Direct Examination of the Transcription Machinery: The model would be more convincingly supported by direct genomic profiling of distinct Pol II states and key regulatory factors. For instance, ChIP-seq analyses of initiation-associated Pol II (e.g., Ser5P), elongation-associated Pol II (Ser2P), pausing factors (e.g., NELF, DSIF), and elongation factors (e.g., BRD4, CDK9) could provide direct evidence for the proposed mechanistic shifts. While the in vitro reconstitution data are valuable, they may not fully capture the regulatory complexity within cells, where the limited transcriptional changes are primarily observed.
2. Data Presentation and Biological Insight: The study relies heavily on aggregate data representations such as global heatmaps and box plots. Including specific genome browser tracks for key genes discussed in the model would greatly enhance the transparency of data quality and provide concrete examples to support the claims. Furthermore, a gene ontology or functional enrichment analysis of the differentially affected gene clusters (e.g., those with balanced, enhanced, or suppressed expression) could yield valuable biological insights into the potential physiological contexts where this cohesin-mediated regulation is most critical.

Reviewer #3

(Remarks to the Author)
This manuscript by Tei et al. addresses a longstanding phenomenon in transcriptional regulation: although the role of cohesin has been established to organize a significant portion of chromatin architecture and facilitate genomic contacts of critical regulatory elements, its depletion results in surprisingly modest changes in both steady-state mRNA levels as well as nascent transcription. The authors propose that cohesin has dual and opposing roles in transcription, both upregulating initiation via promoter contacts and transiently stabilizing the paused RNAP2 complex to regulated pause release. They suggest that cohesin acts as a quality control checkpoint, ensuring proper elongation and transcriptional fidelity. These effects are suggested to offset one another under cohesin depletion, resulting in minimal changes to global transcriptional output.
While the hypothesis is conceptually novel and interesting, and the topic is of broad relevance, the current data and analysis do not sufficiently support the proposed model. The assumption that cohesin's role in bringing chromatin together strictly promotes initiation at genes fails to consider it also brings repressive elements to promoters. This hypothesis also claims that cohesin depletion promotes premature pause release and increased transcription, which the data may support for shorter genes, but the effect on longer genes is not detectable with the current experimental framework. This manuscript holds several scientifically significant observations, but the main conclusions require stronger experimental validation and key assumptions of cohesin's role in transcriptional regulation remain speculative.
At this stage, we do not recommend acceptance of this manuscript at Nature Communications.
Below, we outline specific strengths and concerns that merit further attention

Major Points of Concern:

1. The author's assertion that upon phosphorylation by CDK9 in SEC, RNAP2 is released from pausing and enters productive elongation should be supported with a reference and they should consider that other complexes like P-TEFb also contain CDK9.

2. The heat map figures are difficult to interpret due to their resolution and lack of explicit information regarding the number and identity of genes that are included. If thousands of genes are being shown in a relatively small heat map the number of pixels showing the data is limiting and signal from multiple gene rows are combined, obscuring changes associated with individual genes. This data would be more compelling if transcriptionally up and down genes were shown in separate heat maps and if a few browser shots were provided to display representative examples of these effects beside this heatmap.

3. As stated in the opening paragraph of this review, a main concern is these two functions outlined by the authors are "opposing." Their argument is that cohesin is forming contacts between enhancers and promoters that increase transcription initiation, and this is acting in opposition to cohesin's ability to stabilizes pausing and thereby reduce transcription. Because of these two "opposing" effects the effect of cohesin depletion on transcription is minimal. In our opinion, this is an over simplified view of transcriptional mechanisms of regulation. First, cohesin's role in forming 3D contacts between enhancers and promoters does not exclusively promote transcription initiation, but in some cases is likely to affect a second step in transcription called pause release. Nonetheless, we agree that depletion of cohesion and its loading proteins would reduce gene body transcription, if it acts on either or both these steps. The second proposed activity of cohesion is in stabilizing the paused complexes and thereby allowing the proper quality control and production of a fully functional elongation complex. The depletion of cohesion may prematurely release RNAP2 and lead to some increased transcription, but the authors show this release in cohesion depleted cells leads to less processivity more transcription termination. Therefore, while very short genes may benefit from the increased transcription caused by premature pause release, longer genes (most) would show decreased expression due to the reduced processivity of RNAP2. [Please see the paper from Adelman a couple of years ago (PMCID: PMC3845087) that shows depleting factors associated with pausing (NELF-A and NELF-E) can lead to increase expression of short genes. RNAP2 of longer genes that is not sufficiently modified during pausing will not proceed to a fully productive elongation mode.] Therefore, the two effects of cohesin depletion would be expected to contribute to decreased transcription.

4. Additionally, the transcriptional effects of cohesin depletion appear minimal. For example, in Figure 1b, the log2 fold change in mNET-seq signal ranges only from +0.4 to –0.4, indicating less than a 1.3-fold difference. This modest shift raises concerns about the strength and significance of the reported pausing effects, so conclusions need to be drawn cautiously.

5. To solidify the claims of the role of cohesin depletion, we suggest referring to the Rao et al paper from the Aiden lab (PMID: 25497547), which has high-quality PRO-seq data. The conclusions made about RNAP2 location in this manuscript could be strengthened by reanalyzing bulk data from Fig 7c in that paper. It would be possible to show effects on paused regions in a super enhancer and the regulated paused promoters. It would be worth reanalyzing this data and comparing it to the data being reported here.

6. Another issue with monitored RNAP2 location by RBP1 ChIP-seq and pause release by measuring the Ser2P/RPB1 ratio at promoters. These two confirmation assays use ChIP-seq that does not have the resolution to distinguish RNAP2 at promoters, which transcribe into the gene, and the upstream divergent RNAP2. This caveat in the measurement and the conclusions drawn should be mentioned. As mentioned above, a reanalysis of the Rao et al, Aiden lab data may provide a better confirmation.

7. While we appreciate the efforts of authors to normalize data, especially the challenging ChIP-seq data, we have concerns that normalization in the RNAP2 immunoprecipitation, as accounting for IP efficiency is inherently challenging (for example, the various antibodies binding to proteins from human cells may differ in affinity and specificity relative to proteins from mouse cells). Although the authors use mouse C2C12 spike-in cells to normalize and conclude that a vast majority of TFIID factors on promoters are decreased, the observed effects (Fig. 5) of cohesion depletion are relatively modest and perhaps should be interpreted more cautiously. The effects of cohesin depletion on TFIID binding being up to 91% of genes is concerningly ubiquitous and could point to normalization issues. Additionally, the calculation of the pausing index using only a 1 kb window downstream and a 2 kb region from the transcription start site to represent gene body occupancy is concerning and doesn't make full use of all the gene body data (some genes are hundreds of kb long and you are throwing away that data). It may oversimplify the complexity of RNAP2 distribution. A broader analysis across the full gene body (excluding ~500 bp around the promoter proximal pause and the cleavage polyadenylation sites, which have high levels of RNAP2) would provide a more accurate measure of pausing. Incorporating orthogonal approaches such as PRO-seq or CUT&RUN, or leveraging existing public datasets for this cell line, could strengthen the interpretation and improve confidence in the mapping of paused and elongating RNAP2.

8. The in vitro ATP blocking experiments raise concerns regarding relevance and specificity. What is the promoter and its sequence used in this in vitro experiment? Is the precise pausing position in vitro the same as seen in vivo by NET-seq or PRO-seq? This data could make a compelling case that the in vitro system really recapitulates the in vivo situation. To evaluate the generalizability of the assay, additional promoters could be tested. Additionally, is there any RNA production of this in vitro set up to prove it is a viable system?

9. Using AlFx and ADP to block cohesin's ATPase cycle needs some further clarification. What is the specificity of AlFx and ADP as an ATP mimic, including whether it targets ATP-binding sites selectively and whether certain sites are inhibited more effectively than others? Our understanding is that AlFx and ADP is a general ATPase and GTPase inhibitor. Are conditions used specific for cohesin ATPase and not any other of the many other ATPases associated with transcription?

10. The claim that cohesin and its loader dynamically co-localize with RNAP2 at promoters (Figs 3&4) is not sufficiently supported by the presented co-immunoprecipitation data. This method does not resolve promoter identity; it cannot confirm that the sites bound by transcriptional machinery are the same as those occupied by cohesin loading complexes. It would be important to specify how many genes are included in the meta-data analysis and whether any were excluded based on promoter features or binding profiles. To substantiate co-binding, the authors should show with the ChIP-seq data they have the individual loci where both complexes are clearly co-localized, how many occurrences of co-localization there are and what are the identities of the genes.

Minor Points of Concern:
1. When capturing the pausing state, the authors used a CDK9 inhibitor. In each case, the specific inhibitor needs to be mentioned in the text or figure legend for readers to assess the specificity of the inhibition.
2. When investigating PAF1 association with RNAP2, DRB treatment was used to inhibit CDK9. Why use DRB, which can also inhibit CDK7 (a component of TFIIH), when other inhibitors like flavopiridol and NVP2 and much more specific?
3. The manuscript mentions that triptolide treatment induces degradation of RNAP2. Yes, a three-hour treatment leads to degradation of significant levels of RNAP2. However, the effect of triptolide, which is more important, more rapid and more complete is that its ability to inhibit initiation by inhibiting a helicase of TFIIH.
4. Regarding experiments suggesting cohesin maintains H3K27ac marks, please show precisely where changes in H3K27ac, H3K4me1-marked enhancers, and H3K4me3-positive promoters experience changes by showing clear browser shots. It is also critical to note that a correlation value of -0.35 does not make a strong case that H3K27ac can serve as an accurate proxy for initiation. This tempers greatly the concluding statement of the paragraph "results support the idea that cohesin promotes transcription initiation by maintaining promoter H3K27ac levels, thereby facilitating initiation complex recruitment"
5. When analyzing the contact distances and showing interactions between increased and decreased elements, it would be useful to show some browser shots. Detailed information between individual pairs of enhancers and promoters is lost in this meta-analysis. Effects are overall small, but perhaps examining individual or subsets of genes would be more compelling.
6. Instead of assuming that H3K27ac changing with cohesin loss implies that enhancers serve as a source of transcriptional cofactors, the authors should also consider that it may change the properties of promoters such that they can recruit factors more efficiently.
7. Fig. 4: “and was abolished by CDK9 inhibition (c).” It should be (d).
8. Fig. 1d is missing. Referenced on page 10.
9. In supplementary Fig. 3, triptolide inhibition of initiation does not produce the expected wave of blocked transcription along SLC25A33. Why might this be? Was sufficient triptolide added to block initiation. See PMID:2483027 for an example of patterns produced by a triptolide time course. Labeling the length of the gene would also be helpful in interpreting the figure.
10. Fig. 3. Why is the loading input sometimes less intense than the bands of pulled down fractions? What fraction of input is used? Is it the same for all experiments.
Strengths of manuscript:
1. The in vitro experimental set up was intriguing (Fig. 4) and a further study on its viability as a model for in vivo paused transcription in transient intermediate state between pause and pause release would be relevant and interesting. Perhaps this system should be removed and be its own separate paper, if the controls and further tests show it is a robust and relevant assay.
2. The use of mNET-seq/TT-seq to assess RNAP2 velocity is a technically rigorous approach. These methods provide moderate-resolution insights into transcriptional dynamics and contribute meaningfully to the study’s mechanistic framework.
3. The EU-seq following TNFα treatment provides a useful readout of transcriptional changes under conditions of strong transcriptional activation. Additionally, the use of triptolide and flavopiridol treatments effectively demonstrates how transcription is altered in the context of cohesin depletion. These perturbations help clarify the functional impact of cohesin on transcriptional regulation.
4. The effects on enhancer-promoter communication in cohesion depletions are interesting and makes sense. A focus on changes in specific enhancer-promoter chromatin capture interactions broken down to quartiles could be useful in assessing effects on target promoter composition and activity.
This manuscript reflects a significant investment in data generation and analysis, and the authors have introduced conceptually intriguing hypotheses. With clearer focus and additional targeted experiments and analyses, the existing datasets could be leveraged more effectively to address the central questions concerning the role of cohesion in transcription.

Reviewer #4

(Remarks to the Author)
I co-reviewed this manuscript with one of the reviewers who provided the listed reports. This is part of the Nature Communications initiative to facilitate training in peer review and to provide appropriate recognition for Early Career Researchers who co-review manuscripts.

Version 1:

Reviewer comments:

Reviewer #2

(Remarks to the Author)
The authors have addressed my concerns, thus I support the publication of this work!

Reviewer #3

(Remarks to the Author)
The authors have made a concerted effort to address all of our concerns and have been successful in most cases. A few minor issues remain as listed below.

1. Page 6: Insert “by” after “stabilized”
2. Minor points of concern #9: I don’t accept the rebuttal explanation for the lack of a clear transcriptional wave in Fig.S3. Waves were seen by 20’ of triptolide treatment on hundreds of genes in the first genome-wide measurement of elongation rates of this type. I apologize for the typo in the original review, where I recommended a paper to check as an example of patterns produced by a triptolide treatment time course. Unfortunately, the PMID had a typo and should have read PMID: 24843027, the Jonkers et al paper from 2014. This reference might be a better primary source for the methodology used than the 2023 paper used in reference 17. You might want to use a gene from your data set where the wave clearing is obvious or drop this analysis.
3. Authors may want to check a Deberardine et al (PMID: 37179384) reference for how effects of a prematurely released RNAP2 in a NELF KD can still produce mostly normal transcription unless Cdk9 is inhibited.

Open Access This Peer Review File is licensed under a Creative Commons Attribution 4.0 International License, which permits use, sharing, adaptation, distribution and reproduction in any medium or format, as long as you give appropriate credit to the original author(s) and the source, provide a link to the Creative Commons license, and indicate if changes were made.
In cases where reviewers are anonymous, credit should be given to 'Anonymous Referee' and the source.
The images or other third party material in this Peer Review File are included in the article’s Creative Commons license, unless indicated otherwise in a credit line to the material. If material is not included in the article’s Creative Commons license and your intended use is not permitted by statutory regulation or exceeds the permitted use, you will need to obtain permission directly from the copyright holder.
To view a copy of this license, visit https://creativecommons.org/licenses/by/4.0/

Response to referees

The authors would like to thank the reviewers for their constructive comments and suggestions that helped improve the quality of this manuscript. The manuscript has undergone a thorough revision according to the reviewer's comments. Please see our response below.

Reviewer #1

1. Page 3: '…by briefly associating with the transcription machinery', how brief is 'brief'? Depending on the gene and transcriptional program, the pausing state can be extended. Maybe 'transiently', or 'reversibly' is a better term?

We thank the reviewer for this helpful suggestion. We agree that the term "briefly" may imply a fixed or universally short residence time, which is not appropriate given that the pausing duration can vary across genes. To avoid this unintended implication, we have revised the sentence to use the term "transiently", which more accurately reflects the dynamic nature of the association. The revised texts now read:

Page 2: '…by transiently associating with the transcriptional machinery during the pause–release transition'

Page 3: ' …it stabilizes Pol II pausing by transiently associating with the transcriptional machinery'

2. Page 4: '…decreasing short-range (<1 Mb) and increasing long-range contacts'. How do we know that long-range contacts increase? If cohesin engages in short-range contacts, then loss of these contacts results in a relative, but not an actual, increase in long-range contacts. The quantitative analysis of chromatin interactions remains an important challenge for the chromatin structure field, and it likely goes beyond the scope of the current study to solve this challenge. Until then, however, the authors should be careful with the interpretation of normalised interaction matrices.

We thank the reviewer for this helpful suggestion. We fully agree that normalized Micro-C matrices do not allow us to conclude that long-range contacts "increase" in an absolute sense. Accordingly, we have revised the text to clarify that our observations represent a relative enrichment of long-range contacts rather than a true quantitative increase. The revised text now reads:

Page 4: '…decreasing short-range (<1 Mb) and relatively enriching long-range contacts'

3. Page 4: '...50 of which overlapped with those identified in RAD21-depleted cells'. The authors present what may be one of the most thorough analyses of net gene expression changes following cohesin depletion. Is there anything noteworthy about the list of 50 genes?

We thank the reviewer for this thoughtful question. We performed additional analyses of the 50 overlapping genes, including Gene Ontology enrichment and cell-type specificity. While we did not identify significant enrichment for specific Gene Ontology terms, we found that these genes tend to exhibit more tissue-restricted expression, consistent with Kim et al. (PMID: 41530636). This latter analysis is now included in the revised manuscript (Supplementary Fig. 2j).

4. Page 5: 'Cohesin loss reduced this index,...' The authors should investigate whether this loss is statistically significant.

We thank the reviewer for this comment. While we previously reported P values for each of the four gene groups stratified by the pausing-duration index (Fig. 1d), the gene-wide statistical significance implied by this statement had not been explicitly reported. We have now performed a gene-wide analysis and confirmed a significant reduction of this index upon cohesin loss. The revised text now reads:

Page 5: 'Cohesin loss reduced this index ($P = 3.67 \times 10^{-167}$, Wilcoxon signed-rank test),...'

5. Page 6: 'Given the reduced pausing...', this paragraph is hard to read, despite of its importance. Can this paragraph be rewritten, ideally with less use of acronyms?

We thank the reviewer for this helpful comment. Although the super elongation complex (SEC) was defined in the Introduction, we have added a brief clarification here to improve readability. In addition, we improved the clarity of this paragraph by restructuring its logical flow. Specifically, the revised text first introduces NELF, followed by CDK9 and AFF4 within the context of the SEC, thereby reducing complexity and improving focus.

6. Page 6: '... velocity index – the TT-seq/mNET-seq ratio across gene bodies'. The velocity index appears to be the inverse of the pausing index. Can for simplicity the pausing index be used for this analysis? Or if not, can the relationship between the pausing and the velocity index be made clearer in the text?

We thank the reviewer for this insightful comment. Analogous to the basic relationship that velocity equals distance divided by time, the velocity index is inversely related to the residence time captured by the pausing-duration index. However, the pausing-duration index specifically reflects Pol II behavior at Pol II pausing sites, whereas the velocity index captures transcriptional dynamics across gene bodies. For this reason, we continue to use the velocity index for this analysis. To clarify this relationship, we have added the following sentence to the text:
Page 7: 'Because elongation rate reflects the distance traveled by Pol II per unit time, this velocity index is the reciprocal of the pausing-duration index.'

7. Page 8: '…NIPBL-MAU2 heterodimer, is required not only for loading cohesin onto DNA but also for cohesin's translocation with loop formation'. I am unsure whether this statement is correct. Guérin et al. 2024 found that once cohesin is loaded onto DNA and loops are formed, the translocation of cohesin and the growth of loops occurs independently of the cohesin loader.

We thank the reviewer for pointing this out. We agree that recent work by Guérin et al. (2024) demonstrates that once cohesin is loaded onto DNA and loop formation is initiated, cohesin translocation and loop growth can proceed independently of the cohesin loader. To reflect this nuance, we have revised the text to avoid implying that NIPBL–MAU2 is required for ongoing cohesin translocation. The revised text now reads:

Page 9:'… the NIPBL–MAU2 heterodimer, is required not only for loading cohesin onto DNA but also for the formation of cohesin-mediated loops.'

8. Page 9: '… it is likely that cohesin also interacts with Pol II'. This conclusion is not supported by the data. The authors report that they did not see a direct interaction between cohesin and Pol II, and neither have others. It remains therefore unclear why the authors conclude the opposite? The more genuine conclusion is that the cohesin loader, but not cohesin, directly interact with Pol II. Cohesin in turn likely interacts with this assembly via the loader. The loader-cohesin interaction is known to be stabilized by ADP-AlFx, explaining how cohesin becomes a stable complex component under these conditions.

We thank the reviewer for this important comment. We agree that our data do not support a direct interaction between cohesin and Pol II. Instead, our co-immunoprecipitation experiments demonstrate that the cohesin loader directly associates with Pol II, whereas cohesin itself does not show a detectable interaction. We have therefore revised the text to

suggest that cohesin associates with Pol II indirectly via its loader, rather than directly. The revised text now reads:

Pase 9:' Given their colocalization at promoters observed by ChIP–seq, cohesin may indirectly interact with Pol II via its loader.'

9. Related to the above, the experiment in Figure 4c suggests that cohesin is recruited to the transcription complex only in the presence of ADP-AlFx. However, might cohesin also be recruited to the transcription-loader complex in the absence of ADP-AlFx, but lost again following loading, either by sliding off the linear DNA template, or by ATP hydrolysis-dependent unloading, stimulated by the action of unloading factors such as Pds5-Wapl? Can the authors explore this possibility? It might be tricky to perform a conclusive experiment, but using a doubly tethered DNA or depletion of Wapl might be approaches that might yield insight.

We thank the reviewer for this constructive suggestion. We agree that the absence of a detectable cohesin–transcription complex interaction in the absence of ADP–AlFx does not necessarily indicate a lack of recruitment. Rather, cohesin may be transiently recruited to the transcription–loader complex but rapidly lost following loading. To test whether unloading factors such as Pds5 and Wapl are involved in recruitment, we attempted to deplete them from the nuclear extract, as we did for cohesin and Mediator. However, we were unable to achieve efficient depletion under conditions compatible with the in vitro transcription system.

We also explored the use of doubly tethered DNA as suggested by the reviewer. However, under our current experimental conditions, we did not observe a substantial difference compared with singly tethered DNA, and cohesin was not robustly detected in the absence of ADP–AlFx. These results do not provide conclusive evidence for or against cohesin recruitment under these conditions, as further optimization may be required. Accordingly, we have revised the text to explicitly acknowledge the possibility that cohesin loading can occur without ADP–AlFx:

Page 10: 'Notably, while the loader was present, cohesin itself was not clearly detected on the template.'

10. Page 10: '… Cohesin's ability to entrap and translocate along DNA depends on its ATPase activity… (ref 36)'. This statement is likely incorrect (the reference points to a review that is peripheral to this statement). It is well established that, once loaded

onto DNA, cohesin translocation requires no further ATPase activity. In vitro evidence can be found in several of the single molecule studies that observe cohesin translocation along stretched DNA following loading (most recently Glaser et al. 2025, Figure S1E and note Figure S7B). In vivo evidence is presented in Ocampo et al. 2016.

We thank the reviewer for pointing this out. We agree that the original statement overstated the requirement for ATPase activity during cohesin translocation and that the cited reference did not directly support this claim. Because the role of cohesin ATPase activity is already described in the Introduction in the appropriate context, we have removed this statement from the relevant section.

Reviewer #2

1. Direct Examination of the Transcription Machinery: The model would be more convincingly supported by direct genomic profiling of distinct Pol II states and key regulatory factors. For instance, ChIP-seq analyses of initiation-associated Pol II (e.g., Ser5P), elongation-associated Pol II (Ser2P), pausing factors (e.g., NELF, DSIF), and elongation factors (e.g., BRD4, CDK9) could provide direct evidence for the proposed mechanistic shifts. While the in vitro reconstitution data are valuable, they may not fully capture the regulatory complexity within cells, where the limited transcriptional changes are primarily observed.

We thank the reviewer for this constructive suggestion. Among the factors suggested, we had already performed ChIP-seq analyses for elongation-associated Pol II (Ser2P) and key regulatory factors, including NELF, BRD4, and CDK9, and these data are presented in the original manuscript. In response to the reviewer's suggestion, we performed additional ChIP-seq experiments for initiation-associated Pol II (Ser5P) and DSIF (SPT4), which are included as Supplementary Fig. 7g and Supplementary Fig. 4e. Together with Pol II ChIP-seq data newly obtained in the presence of a CDK7 inhibitor, the Ser5P results indicate that cohesin depletion leads to impaired Pol II recruitment at promoters, rather than decreased initiation efficiency. Accordingly, we have revised the manuscript to replace statements referring to reduced "initiation efficiency" with a more precise description of reduced Pol II recruitment. Furthermore, DSIF ChIP-seq analysis reinforced our conclusion that cohesin depletion shortens Pol II pausing and destabilizes productive elongation.

In addition, to complement the in vitro findings, we performed additional cellular experiments (Fig. 4f and Supplementary Fig. 6h, i). Specifically, upon ADP–AlFx treatment, CUT&Tag analysis revealed increased co-occupancy of Pol II, cohesin, and the cohesin loader at shared promoter regions, consistent with the Pol II–cohesin–loader complex

observed in the reconstituted system. Importantly, this ADP–AlFx–dependent enrichment was abolished upon cohesin depletion in both the reconstituted system and cellular assays, further supporting that the Pol II–cohesin–loader complex formation requires cohesin. Together, these additional analyses strengthen the mechanistic framework proposed in this study.

2. Data Presentation and Biological Insight: The study relies heavily on aggregate data representations such as global heatmaps and box plots. Including specific genome browser tracks for key genes discussed in the model would greatly enhance the transparency of data quality and provide concrete examples to support the claims. Furthermore, a gene ontology or functional enrichment analysis of the differentially affected gene clusters (e.g., those with balanced, enhanced, or suppressed expression) could yield valuable biological insights into the potential physiological contexts where this cohesin-mediated regulation is most critical.

We thank the reviewer for this helpful comment. To enhance data transparency and provide concrete examples supporting our model, we have added representative genome browser tracks. These tracks are now included in Supplementary Fig. 3c, Supplementary Fig. 4g, Supplementary Fig. 5a, Supplementary Fig. 6h, Supplementary Fig. 7a, and Fig. 6a, d.

In addition, to explore the biological characteristics of differentially affected gene clusters, we performed Gene Ontology enrichment analyses and examined cell-type specificity for genes in Clusters 1–3. Gene Ontology analysis identified multiple significantly enriched terms in each cluster (Supplementary Fig. 9f). Notably, analysis of cell-type specificity revealed that genes in the enhanced and suppressed clusters exhibit more cell-type–restricted expression patterns compared with genes in the balanced cluster (Supplementary Fig. 9e).

Reviewer #3

1. The author's assertion that upon phosphorylation by CDK9 in SEC, RNAP2 is released from pausing and enters productive elongation should be supported with a reference and they should consider that other complexes like P-TEFb also contain CDK9.

We thank the reviewer for this important comment. We agree that CDK9 is not exclusive to the super elongation complex (SEC) and is also present in other P-TEFb–containing complexes that promote pause release. To avoid over-specifying the complex involved, we

have revised the text to generalize this statement. We note that the references cited in the preceding sentence already describe the role of CDK9-mediated phosphorylation in Pol II pause release. The revised text now reads:

Page 3:' CDK9 functions within multiple P-TEFb–containing complexes, including SEC.'

2. The heat map figures are difficult to interpret due to their resolution and lack of explicit information regarding the number and identity of genes that are included. If thousands of genes are being shown in a relatively small heat map the number of pixels showing the data is limiting and signal from multiple gene rows are combined, obscuring changes associated with individual genes. This data would be more compelling if transcriptionally up and down genes were shown in separate heat maps and if a few browser shots were provided to display representative examples of these effects beside this heatmap.

We thank the reviewer for this helpful comment. We agree that presenting a large number of genes in a compact heat map can limit interpretability and obscure gene-specific changes. To improve clarity, we have revised the heatmaps in Fig. 7a and Supplementary Fig. 9b by introducing visual separation between gene clusters, allowing clearer distinction among gene groups.

In addition, to provide concrete examples at the level of individual genes or regulatory elements, we have added representative genome browser tracks to complement the heatmap–based analyses. The genome browser tracks are shown in Supplementary Fig. 3c, Supplementary Fig. 4g, Supplementary Fig. 5a, Supplementary Fig. 6h, Supplementary Fig. 7a, and Fig. 6a, d.

Finally, to further enhance transparency, we have updated the figure legends for heatmaps and aggregate plots to explicitly indicate the number of genes or elements included in each analysis.

3. As stated in the opening paragraph of this review, a main concern is these two functions outlined by the authors are "opposing." Their argument is that cohesin is forming contacts between enhancers and promoters that increase transcription initiation, and this is acting in opposition to cohesin's ability to stabilizes pausing and thereby reduce transcription. Because of these two "opposing" effects the effect of cohesin depletion on transcription is minimal. In our opinion, this is an over simplified view of transcriptional mechanisms of regulation. First, cohesin's role in forming 3D

contacts between enhancers and promoters does not exclusively promote transcription initiation, but in some cases is likely to affect a second step in transcription called pause release. Nonetheless, we agree that depletion of cohesion and its loading proteins would reduce gene body transcription, if it acts on either or both these steps. The second proposed activity of cohesion is in stabilizing the paused complexes and thereby allowing the proper quality control and production of a fully functional elongation complex. The depletion of cohesion may prematurely release RNAP2 and lead to some increased transcription, but the authors show this release in cohesion depleted cells leads to less processivity more transcription termination. Therefore, while very short genes may benefit from the increased transcription caused by premature pause release, longer genes (most) would show decreased expression due to the reduced processivity of RNAP2. [Please see the paper from Adelman a couple of years ago (PMCID: PMC3845087) that shows depleting factors associated with pausing (NELF-A and NELF-E) can lead to increase expression of short genes. RNAP2 of longer genes that is not sufficiently modified during pausing will not proceed to a fully productive elongation mode.] Therefore, the two effects of cohesin depletion would be expected to contribute to decreased transcription.

We thank the reviewer for this insightful comment. We agree that enhancer–promoter communication may influence not only transcription initiation but also subsequent steps such as pause release. Accordingly, we have revised the Discussion to include this possibility when considering how cohesin contributes to Pol II pausing. The revised text now reads:

Page 17: ‘Importantly, while our findings suggest that promoter-associated cohesin contributes to pausing regulation, it remains unclear whether these cohesin molecules also participate in promoter–enhancer loops. Therefore, we cannot exclude the possibility that P–E loops themselves influence pausing, a question that remains to be elucidated.’

To investigate whether reduced processivity upon cohesin depletion preferentially affects longer genes, as suggested by the reviewer, we analyzed gene expression changes according to gene length. This analysis did not reveal a preferential reduction in gene expression among longer genes (Response Fig. 1). In fact, longer genes exhibited a slightly milder reduction in expression compared to shorter genes. We speculate that this overall reduction in gene expression across all gene-length groups likely reflects the impact of reduced Pol II promoter recruitment upon cohesin depletion. One possible explanation for the milder effect in longer genes is that cohesin depletion preferentially shortens pausing duration at highly paused genes (Fig. 1d), which tend to be longer (Response Fig. 2). These findings raise the possibility that the reduction in processivity caused by cohesin depletion

may be insufficient to override the potential increase in expression mediated by reduced Pol II pausing. Supporting this interpretation, genes with increased expression upon cohesin depletion tended to show a stronger reduction in Pol II pausing (Cluster 2 in Fig. 7a), and our transcription cycle simulations suggest that shortening pausing can lead to increased gene expression (Fig. 7c). Together, these observations are consistent with the idea that reduced pausing (or enhanced pause release) may contribute to increased gene expression in this context.

In comparison, the Adelman study demonstrated that depletion of NELF, which destabilizes Pol II pausing, does not generally lead to increased gene expression. They suggested that this modest transcriptional effect may result from a combination of reduced pausing and concurrent changes in initiation and elongation. In our analysis, by contrast, reduced pausing is more narrowly defined as a decrease in the dwell time of Pol II at the pause site. In addition, our data indicate that the reduction in pausing observed upon cohesin depletion is unlikely to be mediated by NELF. Instead, our data suggest enhanced activity of pause-release–associated factors, such as CDK9 and AFF4 (Supplementary Fig. 3p, q). We believe that these conceptual and mechanistic differences may help explain the distinct transcriptional outcomes observed in our study compared to theirs.

However, even in the balanced gene cluster (Cluster 1 in Fig. 7a), we observed a slight overall decrease in gene expression (Fig. 7b). Moreover, we observed a modest enhancement in elongation velocity in this cluster (Supplementary Fig. 9c). Together, these observations indicate that transcriptional output cannot be understood as a simple cancellation between initiation and pause release. Accordingly, we have revised the manuscript to avoid implying that a counterbalance between reduced initiation and enhanced pause release results in a net zero change in gene expression.

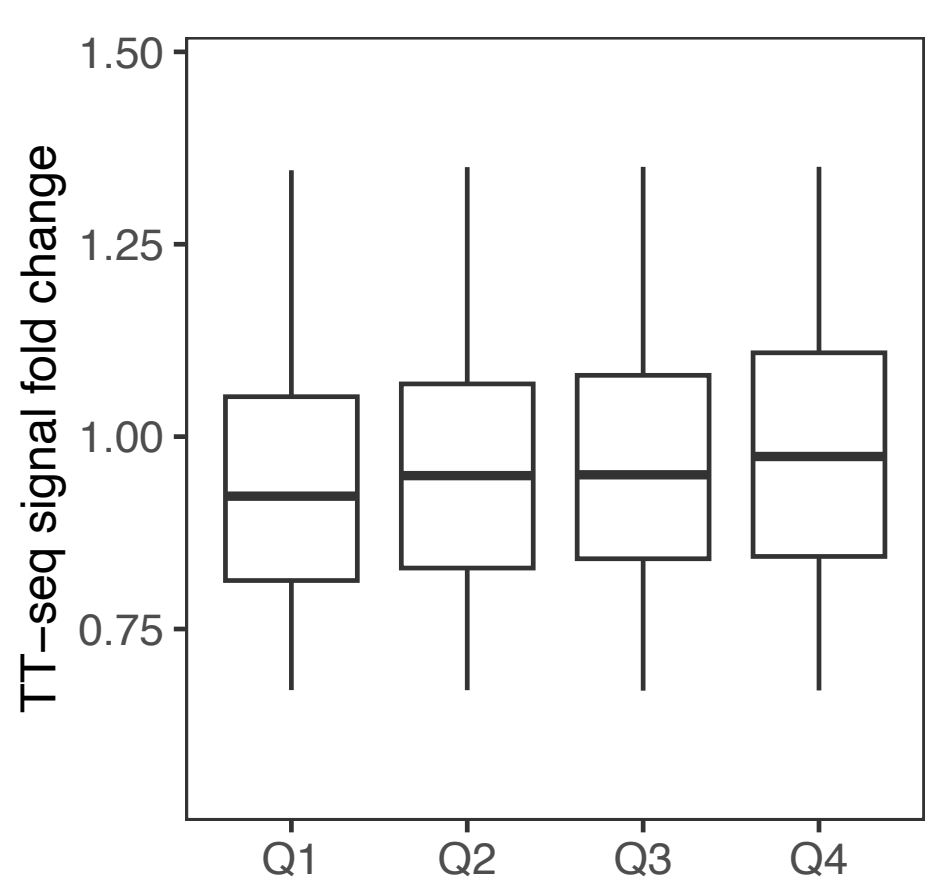

Response Fig. 1 | Box plot showing fold changes (auxin / control) in TT-seq gene expression. The 3,597 genes were evenly divided into four groups based on their length.

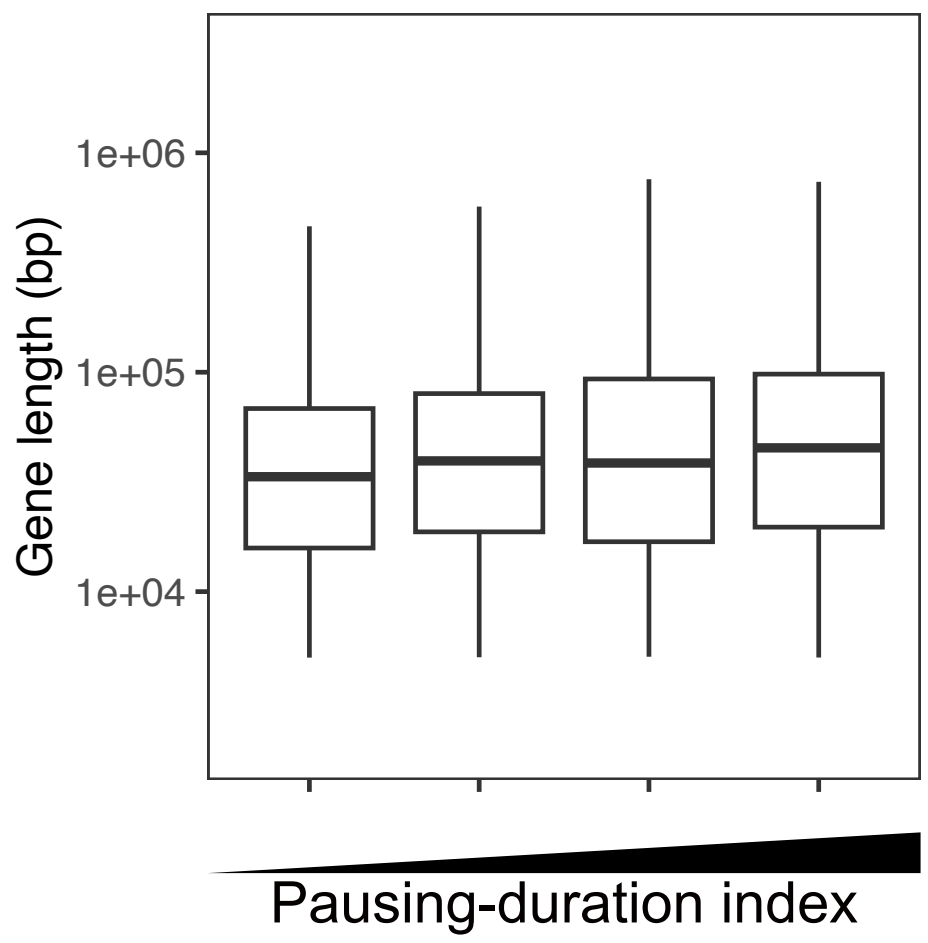

Response Fig. 2 | Box plot showing gene length. The 3,597 genes were evenly divided into four groups based on their pausing-duration index in control cells.

4. Additionally, the transcriptional effects of cohesin depletion appear minimal. For example, in Figure 1b, the log2 fold change in mNET-seq signal ranges only from +0.4 to –0.4, indicating less than a 1.3-fold difference. This modest shift raises concerns about the strength and significance of the reported pausing effects, so conclusions need to be drawn cautiously.

We thank the reviewer for this comment. We agree that the magnitude of transcriptional changes upon cohesin depletion is modest, as reflected by the relatively small dynamic range of mNET-seq signals in Fig. 1b. We therefore interpret these effects cautiously.

Importantly, however, the conclusion that cohesin depletion reduces Pol II pausing is supported by multiple independent and complementary approaches, including mNET-seq/TT-seq–based pausing-duration indices, Ser2P/RPB1 ratios, kinetic measurements using triptolide, Pol II ChIP-seq pausing indices, and PRO-seq analyses (Fig. 1d, Supplementary Fig 3e-k, o). The consistent direction of the effect across these assays indicates that the reduction in pausing is robust despite its modest magnitude.

Moreover, k-means clustering revealed that genes showing a stronger reduction in pausing tend to exhibit increased transcriptional output (Fig. 7a), suggesting that even relatively small changes in pausing can have biologically meaningful consequences for gene regulation. This conclusion is further supported by our transcription cycle simulations, which predict increased transcriptional output upon reduced pausing (Fig. 7c).

5. To solidify the claims of the role of cohesin depletion, we suggest referring to the Rao et al paper from the Aiden lab (PMID: 25497547), which has high-quality PRO-seq

data. The conclusions made about RNAP2 location in this manuscript could be strengthened by reanalyzing bulk data from Fig 7c in that paper. It would be possible to show effects on paused regions in a super enhancer and the regulated paused promoters. It would be worth reanalyzing this data and comparing it to the data being reported here.

We thank the reviewer for this valuable suggestion. In response, we reanalyzed publicly available datasets from Rao et al. (PMID: 25497547) to directly assess whether our conclusions are supported by their data.

First, using the PRO-seq data from Rao et al., we calculated a pausing index and found that cohesin depletion reduced Pol II pausing, consistent with our observations in this study (Supplementary Fig. 3o).

Second, reanalysis of the H3K27ac ChIP-seq data from Rao et al. recapitulated our findings that cohesin depletion redistributes H3K27ac signal at promoters and enhancers (Supplementary Fig. 8b).

Third, we examined the genomic distribution of downregulated genes, as Rao et al. reported that these genes are preferentially located near super-enhancers. Importantly, this pattern was independently reproduced using downregulated genes defined from our TT-seq data (Supplementary Fig. 2h). Moreover, genes in the suppressed gene cluster were similarly enriched near super-enhancers (Supplementary Fig. 9d).

Together, these independent reanalyses of the Rao et al. datasets provide strong external support for our conclusions.

6. Another issue with monitored RNAP2 location by RBP1 ChIP-seq and pause release by measuring the Ser2P/RPB1 ratio at promoters. These two confirmation assays use ChIP-seq that does not have the resolution to distinguish RNAP2 at promoters, which transcribe into the gene, and the upstream divergent RNAP2. This caveat in the measurement and the conclusions drawn should be mentioned. As mentioned above, a reanalysis of the Rao et al, Aiden lab data may provide a better confirmation.

We thank the reviewer for raising this important point. We agree that ChIP-seq–based measurements of RPB1 occupancy and Ser2P/RPB1 ratios at promoters cannot distinguish gene-directed Pol II from upstream divergent transcription, and we have explicitly noted this limitation in the revised manuscript.

Page 6: 'Although ChIP-seq–based measurements cannot distinguish strand-specific Pol II, we combined these analyses with strand-specific approaches.'

Importantly, our conclusion that cohesin depletion reduces Pol II pausing does not rely solely on ChIP-seq–based assays. In addition to RPB1 ChIP-seq and Ser2P/RPB1 measurements, we assessed pausing using multiple independent and complementary approaches, including strand-specific mNET-seq/TT-seq–based pausing-duration indices at pausing sites, which provide higher resolution and transcriptional directionality.

Furthermore, in line with the reviewer's suggestion, we reanalyzed publicly available PRO-seq data from Rao et al. (PMID: 25497547). This independent dataset similarly revealed reduced pausing upon cohesin depletion, providing additional confirmation using a strand-specific assay (Supplementary Fig. 3o). Together, these analyses support the robustness of our conclusions while appropriately accounting for the limitations of ChIP-seq–based measurements.

7. While we appreciate the efforts of authors to normalize data, especially the challenging ChIP-seq data, we have concerns that normalization in the RNAP2 immunoprecipitation, as accounting for IP efficiency is inherently challenging (for example, the various antibodies binding to proteins from human cells may differ in affinity and specificity relative to proteins from mouse cells). Although the authors use mouse C2C12 spike-in cells to normalize and conclude that a vast majority of TFIID factors on promoters are decreased, the observed effects (Fig. 5) of cohesion depletion are relatively modest and perhaps should be interpreted more cautiously. The effects of cohesin depletion on TFIID binding being up to 91% of genes is concerningly ubiquitous and could point to normalization issues. Additionally, the calculation of the pausing index using only a 1 kb window downstream and a 2 kb region from the transcription start site to represent gene body occupancy is concerning and doesn't make full use of all the gene body data (some genes are hundreds of kb long and you are throwing away that data). It may oversimplify the complexity of RNAP2 distribution. A broader analysis across the full gene body (excluding ~500 bp around the promoter proximal pause and the cleavage polyadenylation sites, which have high levels of RNAP2) would provide a more accurate measure of pausing. Incorporating orthogonal approaches such as PRO-seq or CUT&RUN, or leveraging existing public datasets for this cell line, could strengthen the interpretation and improve confidence in the mapping of paused and elongating RNAP2.

We thank the reviewer for this careful assessment of our normalization strategy and data interpretation.

First, the purpose of using mouse C2C12 cells as a spike-in is not to equalize absolute immunoprecipitation efficiencies between human and mouse proteins. Rather, the spike-in serves as an internal reference to control for sample-to-sample variation in chromatin recovery, immunoprecipitation efficiency, and library preparation when the same antibody is used across conditions (see Orlando et al., PMID: 25437568). This approach enables reliable relative comparisons between control and cohesin-depleted samples in our study.

In addition, when both promoters and enhancers are considered, we observed that TAF1 and TBP increase at enhancers showing elevated H3K27ac upon cohesin depletion (see Response Fig. 3 below). Conversely, at promoters, the reduction of TAF1 and TBP is not uniform. Promoters that display a pronounced loss of H3K27ac show a correspondingly stronger decrease in TAF1 and TBP, whereas promoters with smaller changes in H3K27ac exhibit more modest reductions (Fig. 5e and Supplementary Fig. 7d). These results argue against a global normalization bias, which would be expected to affect all loci similarly. Instead, these patterns support our conclusion that cohesin depletion leads to a genuine reduction of TFIID components at promoters.

We also revised the calculation of the Pol II ChIP-seq pausing index by redefining the gene body as the region from TSS + 2 kb to the transcription end site (TES), thereby incorporating Pol II occupancy across the full gene body. Using this broader gene-body definition, we confirmed that cohesin depletion still significantly reduced the pausing index, consistent with our original conclusions (Supplementary Fig. 3i-k). In addition, in line with the reviewer's suggestion to incorporate orthogonal approaches, we calculated pausing indices using strand-specific PRO-seq data from Rao et al. (2017). This independent analysis similarly revealed reduced pausing upon cohesin depletion (Supplementary Fig. 3o), providing additional support for our interpretation that cohesin stabilizes Pol II pausing.

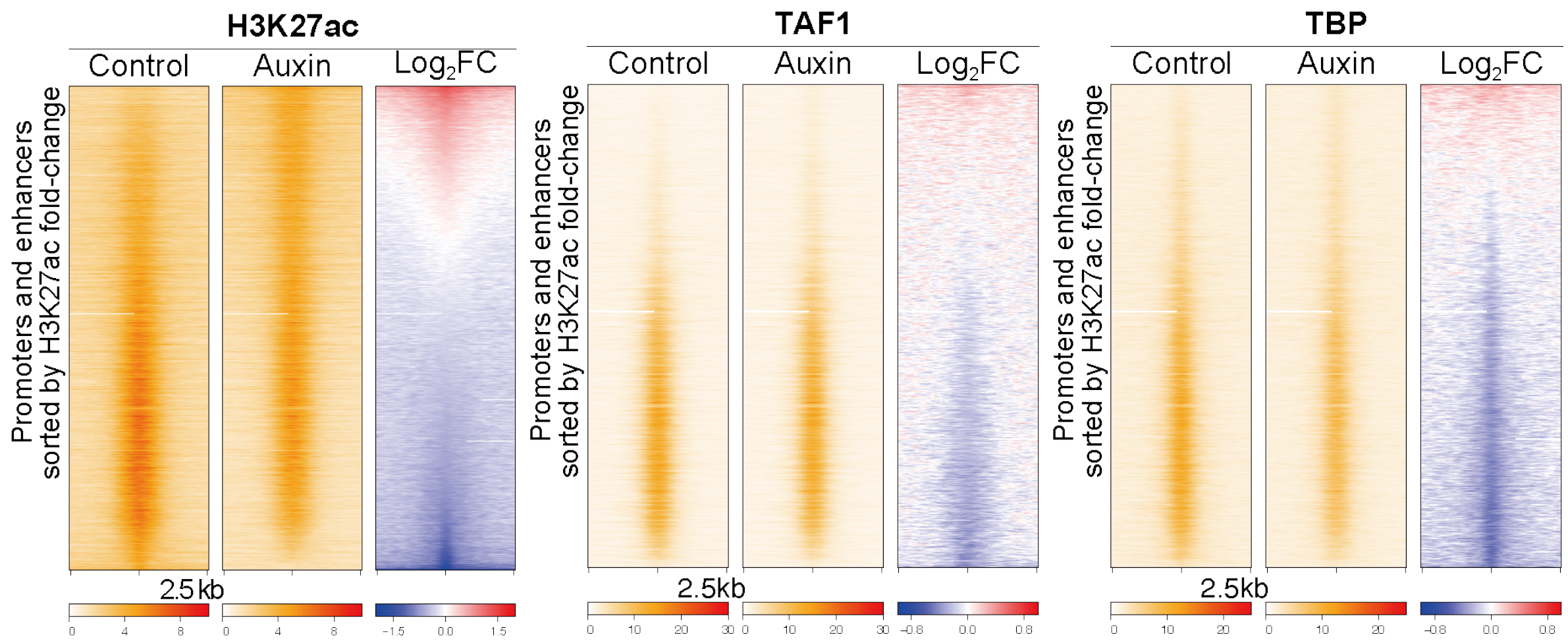

Response Fig. 3 | Heatmaps of H3K27ac, TAF1, and TBP ChIP-seq signals in control and auxin-treated cells at regulatory elements. Regulatory elements are shown in the same order as in Fig. 6a.

8. The in vitro ATP blocking experiments raise concerns regarding relevance and specificity. What is the promoter and its sequence used in this in vitro experiment? Is the precise pausing position in vitro the same as seen in vivo by NET-seq or PRO-seq? This data could make a compelling case that the in vitro system really recapitulates the in vivo situation. To evaluate the generalizability of the assay, additional promoters could be tested. Additionally, is there any RNA production of this in vitro set up to prove it is a viable system?

We thank the reviewer for this important comment. We agree that assessing the physiological relevance and specificity of the in vitro ATP-blocking experiments is essential. The promoter used in this in vitro experiment is an 81-bp minimal promoter region containing a TATA box. This promoter is part of a synthetic DNA that also includes five tandem GAL4 binding sites and a 245-bp fragment from the first intron of the *slc16a1* gene. The precise Pol II pausing position in vitro has not been determined. However, the activator-dependent recruitment of factors and the expected responses to transcriptional inhibitors suggest that key mechanistic aspects of pausing are preserved in this system. To clarify this point, The revised texts now reads:

Page 10: 'Although the precise position of Pol II pausing on this template remains undefined, ...'

Page 16: 'Although the precise Pol II pausing position in vitro has not been determined, ...'

Furthermore, we interpret the ADP–AlFx–stabilized state as a pause–release intermediate based on its composition, which includes pausing- and release-associated factors in addition to Pol II, the cohesin loader, and cohesin, as well as its dependence on CDK9 activity.

To bridge the gap between biochemical and cellular assays, we chose to test whether the key conclusion drawn from the ATP-blocking experiments could also be supported in a cellular context, rather than expanding the in vitro system to additional promoters. To this end, we treated cells with ADP–AlFx to block ATP hydrolysis and performed CUT&Tag analysis (see Fig. 4f and Supplementary Fig. 6h, i). Notably, this cell-based approach partially recapitulated the in vitro observations, revealing enhanced promoter occupancy of Pol II, cohesin, and the cohesin loader under ATP-blocking conditions, which was lost upon cohesin depletion. These results indicate that the ATP-dependent stabilization of the Pol II–cohesin–loader complex observed in vitro reflects a mechanism that can also operate at endogenous promoters in cells.

With respect to RNA production, we directly assessed transcriptional output in the in vitro system by quantitative PCR (Supplementary Fig. 6b). This analysis confirmed that RNA is produced when GAL4–VP16 and ATP are present, demonstrating that the in vitro setup is transcriptionally active.

9. Using AlFx and ADP to block cohesin's ATPase cycle needs some further clarification. What is the specificity of AlFx and ADP as an ATP mimic, including whether it targets ATP-binding sites selectively and whether certain sites are inhibited more effectively than others? Our understanding is that AlFx and ADP is a general ATPase and GTPase inhibitor. Are conditions used specific for cohesin ATPase and not any other of the many other ATPases associated with transcription?

We thank the reviewer for raising this important point regarding the specificity of ADP–AlFx. We agree that ADP–AlFx is not a selective inhibitor of a single ATPase, but rather acts as a transition-state analog that broadly stabilizes ATPase or GTPase active sites in an ADP-bound, hydrolysis-incompetent state.

In our study, ADP–AlFx was used as a biochemical tool to stabilize ATP-bound or pre-hydrolysis conformations of cohesin, rather than to selectively inhibit the cohesin ATPase. Importantly, depletion of cohesin abolished the ADP–AlFx–induced state both in vitro and in vivo. This indicates that the observed effect is cohesin-dependent rather than due to nonspecific ATPase inhibition. Accordingly, we have clarified this interpretation in the revised manuscript. The revised text now reads:

Page 17: 'We note that ADP–AlFx is a general transition-state analog and does not selectively inhibit the cohesin ATPase. However, the observed effect is cohesin-dependent rather than a consequence of nonspecific ATPase inhibition, as depletion of cohesin abolished this complex both in vitro and in vivo.'

10. The claim that cohesin and its loader dynamically co-localize with RNAP2 at promoters (Figs 3&4) is not sufficiently supported by the presented co-immunoprecipitation data. This method does not resolve promoter identity; it cannot confirm that the sites bound by transcriptional machinery are the same as those occupied by cohesin loading complexes. It would be important to specify how many genes are included in the meta-data analysis and whether any were excluded based on promoter features or binding profiles. To substantiate co-binding, the authors should show with the ChIP-seq data they have the individual loci where both complexes are clearly co-localized, how many occurrences of co-localization there are and what are the identities of the genes.

We thank the reviewer for this important comment. We agree that co-immunoprecipitation alone cannot resolve promoter identity and that our original meta-analysis does not, by itself, demonstrate co-localization of cohesin, its loader, and Pol II at the same promoters.

Regarding the meta-analysis, the promoter set used for the average profiles corresponds to the same gene list used throughout the study, comprising 3,597 highly expressed, nonoverlapping genes longer than 5 kb, without additional filtering based on promoter features or binding profiles.

To directly address the reviewer's request for locus-resolved evidence of co-binding, we have now added heatmaps displaying ChIP-seq signals for Pol II, cohesin, and the cohesin loader across this promoter set (Fig. 3a). These heatmaps illustrate the co-occurrence of these factors at promoters. In addition, representative genome browser tracks for individual genes showing clear co-localization are provided to complement the aggregate analyses (Supplementary Fig. 5a).

Minor Points of Concern:

1. When capturing the pausing state, the authors used a CDK9 inhibitor. In each case, the specific inhibitor needs to be mentioned in the text or figure legend for readers to assess the specificity of the inhibition.

We have now specified the CDK9 inhibitor used in each experiment in the main text and corresponding figure legends.

2. When investigating PAF1 association with RNAP2, DRB treatment was used to inhibit CDK9. Why use DRB, which can also inhibit CDK7 (a component of TFIIH), when other inhibitors like flavopiridol and NVP2 and much more specific?

We used DRB because, based on prior experience, it allows rapid and efficient washout, which facilitates assessment of dynamic changes in PAF1 association with Pol II.

3. The manuscript mentions that triptolide treatment induces degradation of RNAP2. Yes, a three-hour treatment leads to degradation of significant levels of RNAP2. However, the effect of triptolide, which is more important, more rapid and more complete is that its ability to inhibit initiation by inhibiting a helicase of TFIIH.

We agree with the reviewer and have revised the text to clarify that the primary effect of triptolide is inhibition of transcription initiation via TFIIH (XPB), with Pol II degradation occurring as a downstream consequence. The revised text now reads:

Page 6: '… we estimated the half-life of paused Pol II by tracking the decay of Pol II promoter binding after triptolide treatment, which blocks new Pol II loading by inhibiting the helicase activity of TFIIH'

4. Regarding experiments suggesting cohesin maintains H3K27ac marks, please show precisely where changes in H3K27ac, H3K4me1-marked enhancers, and H3K4me3-positive promoters experience changes by showing clear browser shots. It is also critical to note that a correlation value of -0.35 does not make a strong case that H3K27ac can serve as an accurate proxy for initiation. This tempers greatly the concluding statement of the paragraph "results support the idea that cohesin promotes transcription initiation by maintaining promoter H3K27ac levels, thereby facilitating initiation complex recruitment"

To clarify where changes in histone modifications occur, we have added representative genome browser tracks showing H3K27ac, H3K4me1-marked enhancers, and H3K4me3-positive promoters (Fig. 6a).

We have also revised the text to avoid interpreting promoter H3K27ac as a direct proxy for transcription initiation efficiency and instead describe it as a marker associated with Pol II recruitment, based on the additional analyses.

The previous correlation analysis was originally included to examine whether the inverse relationship between initiation and pausing reported in earlier studies could be reproduced

in our system. However, given that our revised interpretation indicates that cohesin depletion primarily impacts Pol II promoter recruitment rather than initiation efficiency itself, this analysis is no longer directly relevant to our main conclusion and has therefore been removed.

5. When analyzing the contact distances and showing interactions between increased and decreased elements, it would be useful to show some browser shots. Detailed information between individual pairs of enhancers and promoters is lost in this meta-analysis. Effects are overall small, but perhaps examining individual or subsets of genes would be more compelling.

To address this point, we have added representative genome browser tracks in Fig. 6d, including both Micro-C contact maps and ChIP-seq tracks.

6. Instead of assuming that H3K27ac changing with cohesin loss implies that enhancers serve as a source of transcriptional cofactors, the authors should also consider that it may change the properties of promoters such that they can recruit factors more efficiently.

Based on the simultaneous decrease and increase in H3K27ac at promoters and enhancers, respectively, together with the weakened interactions between these regions, we concluded that H3K27ac redistribution occurs via enhancer-to-promoter communication. However, our analysis does not clarify whether enhancers or promoters play the more active role in this regulatory process. To avoid implying that enhancers function as active suppliers, we have revised the text as follows:

Page 13: ‘Moreover, the observed redistribution of H3K27ac between enhancers and promoters suggests that cohesin-dependent contacts enable functional communication between these regulatory elements. In our model, cohesin depletion disrupts this communication, so that enhancers no longer sustain promoter H3K27ac, resulting in reduced H3K27ac at promoters and increased levels at enhancers (Fig. 6g).’

Page 51: ‘Cohesin-dependent contacts enable functional communication between enhancers and promoters. Cohesin depletion disrupts this communication, so that enhancers no longer sustain promoter H3K27ac, resulting in reduced H3K27ac at promoters and increased levels at enhancers.’

7. Fig. 4: “and was abolished by CDK9 inhibition (c).” It should be (d).

This has been corrected.

8. Fig. 1d is missing. Referenced on page 10.

This has been corrected.

9. In supplementary Fig. 3, triptolide inhibition of initiation does not produce the expected wave of blocked transcription along SLC25A33. Why might this be? Was sufficient triptolide added to block initiation. See PMID:2483027 for an example of patterns produced by a triptolide time course. Labeling the length of the gene would also be helpful in interpreting the figure.

The apparent lack of a clear transcriptional wave along SLC25A33 is due to the very strong RPB1 signal at the promoter, which compresses the dynamic range of the browser view and makes the decay of Pol II signal across the gene body less visually apparent. Triptolide was used at concentrations sufficient to block initiation, as evidenced by the reduction in promoter Pol II.

10. Fig. 3. Why is the loading input sometimes less intense than the bands of pulled-down fractions? What fraction of input is used? Is it the same for all experiments.

The apparent difference in signal intensity between input and immunoprecipitated fractions reflects technical aspects of detection rather than relative abundance. In some experiments, input and IP fractions were detected on separate membranes, and signal contrast was adjusted independently to optimize visualization. Therefore, band intensities between input and IP fractions are not directly comparable. The same fraction of input material was used consistently across experiments, and this has now been clarified in the figure legend.

Response to referees

The authors would like to thank the reviewers for their constructive comments and suggestions, which have helped improve the quality of our manuscript. In this second revision, we have carefully addressed the additional comments raised by Reviewer #3 and revised the manuscript accordingly. Our point-by-point responses are provided below.

Reviewer #3

1. Page 6: Insert “by” after “stabilized”

Thank you very much for pointing this out. We have corrected the text accordingly.

2. Minor points of concern #9: I don’t accept the rebuttal explanation for the lack of a clear transcriptional wave in Fig.S3. Waves were seen by 20’ of triptolide treatment on hundreds of genes in the first genome-wide measurement of elongation rates of this type. I apologize for the typo in the original review, where I recommended a paper to check as an example of patterns produced by a triptolide treatment time course. Unfortunately, the PMID had a typo and should have read PMID: 24843027, the Jonkers et al paper from 2014. This reference might be a better primary source for the methodology used than the 2023 paper used in reference 17. You might want to use a gene from your data set where the wave clearing is obvious or drop this analysis.

We appreciate the reviewer’s suggestion. We have revised Supplementary Figure 3f so that the changes in Pol II distribution across gene bodies upon triptolide treatment can be more clearly visualized, consistent with Figure 1c in the study suggested by the reviewer. We have also replaced reference 17 with this study.

3. Authors may want to check a Deberardine et al (PMID: 37179384) reference for how effects of a prematurely released RNAP2 in a NELF KD can still produce mostly normal transcription unless Cdk9 is inhibited.

We thank the reviewer for this helpful comment. We have considered the DeBerardine et al. study in relation to our data, and tested whether cohesin depletion induces premature Pol II release under CDK9 inhibition, as observed upon NELF depletion. Specifically, we treated cells with flavopiridol to inhibit CDK9 in combination with cohesin depletion. Unlike NELF depletion, cohesin depletion did not lead to premature Pol II release under these

conditions. Thus, our data suggest that cohesin controls pausing duration and/or the timing of pause release through a mechanism distinct from NELF.
