# Supplementary material for: Cohesin acts as a transcriptional gatekeeper by restraining pause–release to promote processive elongation
Source: Nat Commun. 2026 Aug 24;17:8487. doi: 10.1038/s41467-026-76738-3 (PMC13503787; doi:10.1038/s41467-026-76738-3)
Supplement: Supplementary file 3 [file 41467_2026_76738_MOESM3_ESM.pdf]

Corresponding author(s): *Double-anonymous peer review submissions: write DAPR and your manuscript number here instead of author names.*

Last updated by author(s): *YYYY-MM-DD*

Reporting Summary

Nature Portfolio wishes to improve the reproducibility of the work that we publish. This form provides structure for consistency and transparency in reporting. For further information on Nature Portfolio policies, see our Editorial Policies and the Editorial Policy Checklist.

Statistics

For all statistical analyses, confirm that the following items are present in the figure legend, table legend, main text, or Methods section.

n/a	Confirmed	
☐	☒	The exact sample size (*n*) for each experimental group/condition, given as a discrete number and unit of measurement
☐	☒	A statement on whether measurements were taken from distinct samples or whether the same sample was measured repeatedly
☐	☒	The statistical test(s) used AND whether they are one- or two-sided<br>*Only common tests should be described solely by name; describe more complex techniques in the Methods section.*
☒	☐	A description of all covariates tested
☐	☒	A description of any assumptions or corrections, such as tests of normality and adjustment for multiple comparisons
☐	☒	A full description of the statistical parameters including central tendency (e.g. means) or other basic estimates (e.g. regression coefficient) AND variation (e.g. standard deviation) or associated estimates of uncertainty (e.g. confidence intervals)
☐	☒	For null hypothesis testing, the test statistic (e.g. *F*, *t*, *r*) with confidence intervals, effect sizes, degrees of freedom and *P* value noted<br>*Give P values as exact values whenever suitable.*
☒	☐	For Bayesian analysis, information on the choice of priors and Markov chain Monte Carlo settings
☒	☐	For hierarchical and complex designs, identification of the appropriate level for tests and full reporting of outcomes
☐	☒	Estimates of effect sizes (e.g. Cohen's *d*, Pearson's *r*), indicating how they were calculated

Our web collection on statistics for biologists contains articles on many of the points above.

Software and code

Policy information about availability of computer code

Data collection	Flow cytometry was analysed using BD Accuri C6 plus flow cytometer with BD Accuri C6 Plus Software v0.34.1.<br>qPCR date were collected using StepOnePlus real-time PCR systems with StepOne Software v2.3.<br>Sequencing libraries were run on HiSeq 2500, NextSeq 2000, NextSeq 550, NovaSeq 6000, or NextSeq 1000.<br>Western blot images were acquired using FUSION FX7 Edge with Fusion FX7 Edge v18.03.
Data analysis	Sequencing data were analysed using Bowtie v1.2.3, Bowtie2 v2.4.5 and v2.5.3, STAR v2.7.10a, RSEM v1.3.3, edgeR v4.2.2, DESeq2 v1.46.0, SAMtools v1.15.1, htseq-count v1.99.2, Trimmomatic v0.39, BWA-MEM v0.75, parse2wig v3.7.2, drompa_peakcall v3.7.2, drompa+ PC_SHARP v1.17.2, drompa3 v3.7.2, deepTools v3.5.1, Pairtools v0.3.0, Juicer v1.22.01, Mustache v1.2.0, Chromosight v1.6.1, HiCExplorer v3.7.2, coolpup.py v1.0.0, Churros v1.5.0, DANPOS3 v2.2.2 and ChromHMM v1.25.<br>Western blot band intensities were quantified using ImageJ v1.53c.<br>Flow cytometry data were analysed using FlowJo v9.0.1.

For manuscripts utilizing custom algorithms or software that are central to the research but not yet described in published literature, software must be made available to editors and reviewers. We strongly encourage code deposition in a community repository (e.g. GitHub). See the Nature Portfolio guidelines for submitting code & software for further information.

Data

Policy information about availability of data

All manuscripts must include a data availability statement. This statement should provide the following information, where applicable:

- Accession codes, unique identifiers, or web links for publicly available datasets
- A description of any restrictions on data availability
- For clinical datasets or third party data, please ensure that the statement adheres to our policy

The sequencing data generated in this study have been deposited in the Gene Expression Omnibus (GEO) under accession codes GSE260854 [https://www.ncbi.nlm.nih.gov/geo/query/acc.cgi?acc=GSE260854] (Micro-C), GSE260852 [https://www.ncbi.nlm.nih.gov/geo/query/acc.cgi?acc=GSE260852] (ChIP-seq), GSE300575 [https://www.ncbi.nlm.nih.gov/geo/query/acc.cgi?acc=GSE300575] (ChIP-seq), GSE300574 [https://www.ncbi.nlm.nih.gov/geo/query/acc.cgi?acc=GSE300574] (TT-seq), GSE300576 [https://www.ncbi.nlm.nih.gov/geo/query/acc.cgi?acc=GSE300576] (mNET-seq), GSE300577 [https://www.ncbi.nlm.nih.gov/geo/query/acc.cgi?acc=GSE300577] (RNA-seq), GSE260853 [https://www.ncbi.nlm.nih.gov/geo/query/acc.cgi?acc=GSE260853] (EU-seq), and GSE336388 [https://www.ncbi.nlm.nih.gov/geo/query/acc.cgi?acc=GSE336388] (CUT&Tag). Western blot and qPCR data are provided in the Source Data file.

Research involving human participants, their data, or biological material

Policy information about studies with human participants or human data. See also policy information about sex, gender (identity/presentation), and sexual orientation and race, ethnicity and racism.

Reporting on sex and gender	n/a
Reporting on race, ethnicity, or other socially relevant groupings	n/a
Population characteristics	n/a
Recruitment	n/a
Ethics oversight	n/a

Note that full information on the approval of the study protocol must also be provided in the manuscript.

Field-specific reporting

Please select the one below that is the best fit for your research. If you are not sure, read the appropriate sections before making your selection.

[x] Life sciences [ ] Behavioural & social sciences [ ] Ecological, evolutionary & environmental sciences

For a reference copy of the document with all sections, see nature.com/documents/nr-reporting-summary-flat.pdf

Life sciences study design

All studies must disclose on these points even when the disclosure is negative.

Sample size	No statistical method was used to predetermine sample size. Sample sizes were based on previous studies in the field and are consistent with standard practice for genome-wide assays. All experiments were performed with at least two or three independent biological replicates as indicated in the figure legends.
Data exclusions	No data were excluded from the analyses.
Replication	All experiments were replicated independently with similar results. The number of biological replicates is indicated in the figure legends. Reproducibility between replicates was confirmed by correlation analysis (Supplementary Tables 1 and 2).
Randomization	Randomization was not relevant to this study, as experiments were performed in cultured cell lines under controlled laboratory conditions.
Blinding	Blinding was not relevant to this study, as outcome assessment was based on automated computational analyses.

Reporting for specific materials, systems and methods

We require information from authors about some types of materials, experimental systems and methods used in many studies. Here, indicate whether each material, system or method listed is relevant to your study. If you are not sure if a list item applies to your research, read the appropriate section before selecting a response.

Materials & experimental systems

n/a	Involved in the study
[ ]	[x] Antibodies
[ ]	[x] Eukaryotic cell lines
[x]	[ ] Palaeontology and archaeology
[x]	[ ] Animals and other organisms
[x]	[ ] Clinical data
[x]	[ ] Dual use research of concern
[x]	[ ] Plants

Methods

n/a	Involved in the study
[ ]	[x] ChIP-seq
[ ]	[x] Flow cytometry
[x]	[ ] MRI-based neuroimaging

Antibodies

Antibodies used	Rpb1 NTD (D8L4Y) (Cell Signaling Technology, #14958);<br>Rabbit polyclonal anti-RAD21 (Sakata et al.);<br>Mouse monoclonal anti-MAU2 (Sakata et al.);<br>Purified Rabbit Anti-GFP (Torrey Pines Biolabs, TP401);<br>Anti-Phospho RNA Polymerase II CTD (Ser2) (Monoclonal research institute Inc., MABI0602);<br>anti-acetyl Histone H3 (Lys27) (MBL International, MABI0309);<br>anti-trimethyl Histone H3 (Lys4) (MBL International, MABI0304);<br>anti-monomethyl Histone H3 (Lys4) (MBL International, MABI0302);<br>Rabbit anti-MCEF (Bethyl, A302-539A);<br>TH1L (D5G6W) (Cell Signaling Technology, #12265);<br>Anti-Cdk9 (Abcam, #ab239364);<br>Rabbit anti-TAF1 (Bethyl, #A303-505A);<br>Anti-TBP (Abcam, #ab300656);<br>Rabbit Anti-GFP antibody (abcam, #ab290);<br>Rpb1 CTD (4H8) Mouse Monoclonal Antibody (Cell Signaling Technology, #2629);<br>Anti-Rabbit Secondary Antibody for CUTANA™ CUT&Tag Workflows (EpiCypher, #1047);<br>Rabbit Anti-Mouse IgG H&L (abcam, #ab46540)<br>Rabbit Anti-GFP (Abcam, #ab290);<br>Rpb1 CTD (4H8) (Cell Signaling Technology, #2629);<br>Anti-RNA Polymerase II CTD (Fujifilm, #384-09271);<br>Anti-Phospho RNA Polymerase II CTD (Ser2) (Fujifilm, #381-09281);<br>Anti-DYKDDDDK tag magnetic beads (Fujifilm, #017-25151);<br>Nipbl (Short form) Rat monoclonal antibody (ORIGENE, #AM20797PU-N).<br>Secondary antibodies included Anti-Rabbit Secondary Antibody for CUTANA™ CUT&Tag workflows (EpiCypher, #1047) and Rabbit Anti-Mouse IgG H&L (Abcam, #ab46540)
Validation	The specificity of antibodies against RAD21, SMC1, WAPL, GFP, RPB1, Ser2-phosphorylated RPB1, NIPBL, MAU2, and H3K27ac was validated in this study by depletion or pharmacological inhibition of the corresponding targets, which resulted in loss or marked reduction of signal.<br>For all other antibodies, validation was supported by manufacturer documentation and prior publications.

Eukaryotic cell lines

Policy information about cell lines and Sex and Gender in Research

Cell line source(s)	HCT116 cells were originally obtained from ATCC. Auxin-inducible degron cell lines were generated in-house as described in the Methods.
Authentication	The cell lines were not re-authenticated in this study but were obtained from reputable sources and maintained under standard laboratory conditions.
Mycoplasma contamination	All cell lines tested negative for mycoplasma contamination.
Commonly misidentified lines<br>(See ICLAC register)	The cell lines used in this study are not listed in the ICLAC database of commonly misidentified cell lines.

Plants

Seed stocks: n/a

Novel plant genotypes: n/a

Authentication: n/a

ChIP-seq

Data deposition

☒ Confirm that both raw and final processed data have been deposited in a public database such as GEO.

☐ Confirm that you have deposited or provided access to graph files (e.g. BED files) for the called peaks.

Data access links
May remain private before publication.

GSE260854 [https://www.ncbi.nlm.nih.gov/geo/query/acc.cgi?acc=GSE260854] (Micro-C)
GSE260852 [https://www.ncbi.nlm.nih.gov/geo/query/acc.cgi?acc=GSE260852] (ChIP-seq)
GSE300575 [https://www.ncbi.nlm.nih.gov/geo/query/acc.cgi?acc=GSE300575] (ChIP-seq)
GSE300574 [https://www.ncbi.nlm.nih.gov/geo/query/acc.cgi?acc=GSE300574] (TT-seq)
GSE300576 [https://www.ncbi.nlm.nih.gov/geo/query/acc.cgi?acc=GSE300576] (mNET-seq)
GSE300577 [https://www.ncbi.nlm.nih.gov/geo/query/acc.cgi?acc=GSE300577] (RNA-seq)
GSE260853 [https://www.ncbi.nlm.nih.gov/geo/query/acc.cgi?acc=GSE260853] (EU-seq)
GSE336388 [https://www.ncbi.nlm.nih.gov/geo/query/acc.cgi?acc=GSE336388] (CUT&Tag).

Files in database submission

GSM8125645 ChIPseq, RPB1, ctrl, rep1
GSM8125646 ChIPseq, RPB1, aux, rep1
GSM8125647 ChIPseq, Input, RPB1, ctrl, rep1
GSM8125648 ChIPseq, Input, RPB1, aux, rep1
GSM8125649 ChIPseq, RPB1, ctrl, rep2
GSM8125650 ChIPseq, RPB1, aux, rep2
GSM8125651 ChIPseq, Input, RPB1, ctrl, rep2
GSM8125652 ChIPseq, Input, RPB1, aux, rep2
GSM8125653 ChIPseq, RPB1, ctrl, flav, 0
GSM8125654 ChIPseq, RPB1, aux, flav, 0
GSM8125655 ChIPseq, Input, RPB1, ctrl, flav, 0
GSM8125656 ChIPseq, Input, RPB1, aux, flav, 0
GSM8125657 ChIPseq, RPB1, ctrl, flav, 10
GSM8125658 ChIPseq, RPB1, aux, flav, 10
GSM8125659 ChIPseq, Input, RPB1, ctrl, flav, 10
GSM8125660 ChIPseq, Input, RPB1, aux, flav, 10
GSM8125661 ChIPseq, RPB1, ctrl, flav, 15
GSM8125662 ChIPseq, RPB1, aux, flav, 15
GSM8125663 ChIPseq, Input, RPB1, ctrl, flav, 15
GSM8125664 ChIPseq, Input, RPB1, aux, flav, 15
GSM8125665 ChIPseq, Ser2P, ctrl
GSM8125666 ChIPseq, Ser2P, aux
GSM8125667 ChIPseq, H3K27ac, ctrl, rep1
GSM8125668 ChIPseq, H3K27ac, aux, rep1
GSM8125669 ChIPseq, Input, H3K27ac, ctrl, rep1
GSM8125670 ChIPseq, Input, H3K27ac, aux, rep1
GSM8125671 ChIPseq, H3K27ac, ctrl, rep2
GSM8125672 ChIPseq, H3K27ac, aux, rep2
GSM8125673 ChIPseq, Input, H3K27ac, ctrl, rep2
GSM8125674 ChIPseq, Input, H3K27ac, aux, rep2
GSM8125675 ChIPseq, H3K4me1, ctrl
GSM8125676 ChIPseq, H3K4me1, aux
GSM8125677 ChIPseq, Input, H3K4me1, ctrl
GSM8125678 ChIPseq, Input, H3K4me1, aux
GSM8125679 ChIPseq, H3K4me3, ctrl
GSM8125680 ChIPseq, H3K4me3, aux
GSM8125681 ChIPseq, Input, H3K4me3, ctrl

GSM8125682 ChIPseq, Input, H3K4me3, aux
GSM8125683 ChIPseq, NELFCD, ctrl, rep1
GSM8125684 ChIPseq, NELFCD, aux, rep1
GSM8125685 ChIPseq, Input, Ser2P, NELFCD, ctrl
GSM8125686 ChIPseq, Input, Ser2P, NELFCD, aux
GSM8125687 ChIPseq, AFF4, ctrl
GSM8125688 ChIPseq, AFF4, aux
GSM8125689 ChIPseq, Input, AFF4, ctrl
GSM8125690 ChIPseq, Input, AFF4, aux
GSM8125691 ChIPseq, RPB1, ctrl, SMC1
GSM8125692 ChIPseq, RPB1, aux, SMC1
GSM8125693 ChIPseq, Input, RPB1, ctrl, SMC1
GSM8125694 ChIPseq, Input, RPB1, aux, SMC1
GSM8125695 ChIPseq, AFF4, DRB, ctrl
GSM8125696 ChIPseq, AFF4, DRB, aux
GSM8125697 ChIPseq, Input, AFF4, DRB, ctrl
GSM8125698 ChIPseq, Input, AFF4, DRB, aux
GSM8125699 ChIPseq, RPB1, KL1, ctrl
GSM8125700 ChIPseq, RPB1, KL1, aux
GSM8125701 ChIPseq, Input, RPB1, KL1, ctrl
GSM8125702 ChIPseq, Input, RPB1, KL1, aux
GSM8125703 ChIPseq, RPB1, ctrlKD
GSM8125704 ChIPseq, RPB1, WaplKD
GSM8125705 ChIPseq, GFP, ctrlKD
GSM8125706 ChIPseq, GFP, WaplKD
GSM8125707 ChIPseq, Input, RPB1, GFP, ctrlKD
GSM8125708 ChIPseq, Input, RPB1, GFP, WaplKD
GSM8125709 ChIPseq, NELFCD, flav, ctrl
GSM8125710 ChIPseq, NELFCD, flav, aux
GSM8125711 ChIPseq, Input, NELFCD, flav
GSM8125712 ChIPseq, Input, NELFCD, flav, aux
GSM8125713 EUseq, ctrl, rep1
GSM8125714 EUseq, aux, rep1
GSM8125715 EUseq, ctrl, rep2
GSM8125716 EUseq, aux, rep2
GSM8125717 MicroC, control
GSM8125718 MicroC, IAA
GSM9064729 TTseq, ctrl, rep1
GSM9064730 TTseq, aux, rep1
GSM9064731 TTseq, ctrl, rep2
GSM9064732 TTseq, aux, rep2
GSM9064733 ChIPseq, BRD4, ctrl
GSM9064734 ChIPseq, BRD4, aux
GSM9064735 ChIPseq, MED1, ctrl
GSM9064736 ChIPseq, MED1, aux
GSM9064737 ChIPseq, Input, RPB1, control
GSM9064738 ChIPseq, Input, RPB1, DRB
GSM9064739 ChIPseq, Input, RPB1, release
GSM9064740 ChIPseq, RPB1, control
GSM9064741 ChIPseq, RPB1, DRB
GSM9064742 ChIPseq, RPB1, release
GSM9064743 ChIPseq, Input, control
GSM9064744 ChIPseq, Input, DRB
GSM9064745 ChIPseq, Input, release
GSM9064746 ChIPseq, RAD21, control
GSM9064747 ChIPseq, RAD21, DRB
GSM9064748 ChIPseq, RAD21, release
GSM9064749 ChIPseq, NIPBL, control
GSM9064750 ChIPseq, NIPBL, DRB
GSM9064751 ChIPseq, NIPBL, release
GSM9064752 ChIPseq, MAU2, control
GSM9064753 ChIPseq, MAU2, DRB
GSM9064754 ChIPseq, MAU2, release
GSM9064755 mNETseq, ctrl, rep1
GSM9064756 mNETseq, aux, rep1
GSM9064757 mNETseq, ctrl, rep2
GSM9064758 mNETseq, aux, rep2
GSM9064759 mNETseq, ctrl, rep3
GSM9064760 mNETseq, aux, rep3

GSM9064761 RNAseq, ctrl, rep1
GSM9064762 RNAseq, aux, rep1
GSM9064763 RNAseq, ctrl, rep2
GSM9064764 RNAseq, aux, rep2
GSM9064765 RNAseq, ctrl, rep3
GSM9064766 RNAseq, aux, rep3
GSM9064767 RNAseq, XRN2, KD, rep1
GSM9064768 RNAseq, aux, XRN2, KD, rep1
GSM9064769 RNAseq, XRN2, KD, rep2
GSM9064770 RNAseq, aux, XRN2, KD, rep2
GSM9064771 RNAseq, XRN2, KD, rep3
GSM9064772 RNAseq, aux, XRN2, KD, rep3
GSM9064773 RNAseq, SMC1, ctrl, rep1
GSM9064774 RNAseq, SMC1, aux, rep1
GSM9064775 RNAseq, SMC1, ctrl, rep2
GSM9064776 RNAseq, SMC1, aux, rep2
GSM9064777 RNAseq, SMC1, ctrl, rep3
GSM9064778 RNAseq, SMC1, aux, rep3
GSM9834056 CUTandTAG, RAD21, ctrl
GSM9834057 CUTandTAG, RAD21, ctrl, ATP
GSM9834058 CUTandTAG, RAD21, ctrl, AlFx
GSM9834059 CUTandTAG, RAD21, aux2
GSM9834060 CUTandTAG, RAD21, aux2, ATP
GSM9834061 CUTandTAG, RAD21, aux2, AlFx
GSM9834062 CUTandTAG, RPB1, ctrl
GSM9834063 CUTandTAG, RPB1, ctrl, ATP
GSM9834064 CUTandTAG, RPB1, ctrl, AlFx
GSM9834065 CUTandTAG, RPB1, aux2
GSM9834066 CUTandTAG, RPB1, aux2, ATP
GSM9834067 CUTandTAG, RPB1, aux2, AlFx
GSM9834068 CUTandTAG, NIPBL, ctrl
GSM9834069 CUTandTAG, NIPBL, ctrl, ATP
GSM9834070 CUTandTAG, NIPBL, ctrl, AlFx

Genome browser session (e.g. UCSC)

Not applicable.

Methodology

Replicates

All ChIP-seq experiments were performed with at least two independent biological replicates. Reproducibility between replicates was confirmed by genome-wide correlation analysis.

Sequencing depth

see Methods

Antibodies

Rpb1 NTD (D8L4Y) (Cell Signaling Technology, #14958);
Rabbit polyclonal anti-RAD21 (Sakata et al.);
Mouse monoclonal anti-MAU2 (Sakata et al.);
Purified Rabbit Anti-GFP (Torrey Pines Biolabs, TP401);
Anti-Phospho RNA Polymerase II CTD (Ser2) (Monoclonal research institute Inc., MABI0602);
anti-acetyl Histone H3 (Lys27) (MBL International, MABI0309);
anti-trimethyl Histone H3 (Lys4) (MBL International, MABI0304);
anti-monomethyl Histone H3 (Lys4) (MBL International, MABI0302);
Rabbit anti-MCEF (Bethyl, A302-539A);
TH1L (D5G6W) (Cell Signaling Technology, #12265);
Anti-Cdk9 (Abcam, #ab239364);
Rabbit anti-TAF1 (Bethyl, #A303-505A);
Anti-TBP (Abcam, #ab300656)

Peak calling parameters

Peak calling was performed using drompa_peakcall (v3.7.2) in “PC_SHARP” mode with default parameters unless otherwise specified. Spike-in normalization was applied prior to peak calling.

Data quality

Data quality was assessed by replicate correlation analysis.

Software

Data were processed using Bowtie (v1.2.3), drompa_peakcall (v3.7.2), drompa+ (v1.17.2), and deepTools (v3.5.1), as described in the Methods.
The analysis code used in this study has been deposited in Zenodo under DOI 10.5281/zenodo.21257761 [https://zenodo.org/records/21257761].

Flow Cytometry

Plots

Confirm that:

- [x] The axis labels state the marker and fluorochrome used (e.g. CD4-FITC).
- [x] The axis scales are clearly visible. Include numbers along axes only for bottom left plot of group (a 'group' is an analysis of identical markers).
- [x] All plots are contour plots with outliers or pseudocolor plots.
- [x] A numerical value for number of cells or percentage (with statistics) is provided.

Methodology

Sample preparation	Cells were fixed in 70% ethanol, washed with PBS, treated with RNase A (50 µg/mL), and stained with propidium iodide (50 µg/mL).
Instrument	Flow cytometry data were collected using a BD Accuri C6 plus flow cytometer.
Software	Flow cytometry data were collected using BD Accuri C6 Plus Software v1.0.34.1 and analyzed using FlowJo v9.0.1.
Cell population abundance	Population abundance and purity were determined as the percentage of events within each gate based on the flow cytometry gating statistics. Detailed cell numbers and percentages for each individual sample are provided in the Source Data.
Gating strategy	Cells were first gated to exclude debris and low-quality events using FSC-H and PE-H parameters. The main cell population was selected as P1. Doublets and aggregates were excluded using PE-A and PE-H, and the resulting P2 single-cell population was used for DNA-content analysis. Gate boundaries were defined using the control sample and applied consistently across all samples.

- [x] Tick this box to confirm that a figure exemplifying the gating strategy is provided in the Supplementary Information.
