# Supplementary material for: Cohesin acts as a transcriptional gatekeeper by restraining pause–release to promote processive elongation
Source: Nat Commun. 2026 Aug 24;17:8487. doi: 10.1038/s41467-026-76738-3 (PMC13503787; doi:10.1038/s41467-026-76738-3)
Supplement: Supplementary file 4 [file 41467_2026_76738_MOESM4_ESM.pdf]

Description of Additional Supplementary Files

File Name: Supplementary Data 1
Description: Spearman correlation between biological replicates

File Name: Supplementary Data 2

Description: QuASAR-Rep reproducibility scores for Micro-C replicates
